# Prevalence and Predictors of Nonresponse to Psychological Treatment for PTSD: A Meta-Analysis

**DOI:** 10.1155/2024/9899034

**Published:** 2024-07-26

**Authors:** Verena Semmlinger, Cosima Leithner, Lea Maria Klöck, Lena Ranftl, Thomas Ehring, Monika Schreckenbach

**Affiliations:** ^1^Department of Psychology, Ludwig-Maximilians-University Munich, Munich 80802, Germany; ^2^German Center for Mental Health (DZPG), Munich 80802, Germany

## Abstract

**Background:**

Although highly efficacious psychological treatments for posttraumatic stress disorder (PTSD) exist, there is evidence that first-line psychological treatment approaches leave a substantial subgroup of patients still suffering from clinically relevant PTSD symptoms posttreatment.

**Aims:**

We aimed to meta-analytically establish the prevalence and predictors of nonresponse to first-line guideline-recommended psychological treatments for PTSD.

**Materials and Methods:**

This meta-analysis was preregistered (CRD42023368766). We searched the PTSD Trials Standardized Data Repository, Embase, Medline, PsychINFO, and PTSDpubs. We included randomized controlled trials (RCT), reporting data on nonresponse operationalized by (lack of) symptom reduction in PTSD symptoms at posttreatment of first-line guideline-recommended PTSD treatments for adult patients meeting criteria for a PTSD diagnosis. All studies published by October 10, 2023, were included. Data were extracted by two independent reviewers. We estimated the pooled average nonresponse rates and ORs. Subgroup and metaregression analyses targeting the nonresponse rates served to identify significant predictors. All analyses were conducted using three-level multilevel models. Study quality was assessed using Cochrane's RoB 2 tool.

**Results:**

Eighty six studies with 117 active treatment conditions and 7,894 participants were included in the meta-analysis. The weighted average nonresponse rate was 39.23%, 95% CI (35.08%, 43.53%). Nonresponse was less frequent in the treatment condition compared to the control condition (*OR* = 0.22). Subgroup analyses and metaregression revealed the type of analysis, population, type of intervention, treatment format, year of publication, age, sex, PTSD symptom severity, comorbid depression, and baseline depression score as significant predictors. The heterogeneity between studies was substantial to considerable (*I*^2^ = 83.12%). Half of the studies had a high risk of bias.

**Conclusions:**

This meta-analysis found that a substantial subgroup of patients suffering from PTSD still showed clinically significant symptoms after having received treatment. Treatment modifications should be considered for specific subgroups of PTSD patients based on predictors found to be associated with nonresponse.

## 1. Introduction

In recent decades, highly efficacious psychological treatments for posttraumatic stress disorder (PTSD) have been developed, with trauma-focused interventions as first-line guideline-recommended treatments for PTSD [1, 2]. However, researchers have recently raised serious concerns about methodological issues as well as reporting standards of PTSD trials [3, 4]. Importantly, as effect sizes were typically reported on the group level, the number of trial participants not responding to treatment or even showing symptom worsening has been left largely unreported [5, 6]. For instance, between 2010 and 2020, operational definitions for treatment nonresponse were provided in only 60% of PTSD trials [6]. In addition, there is a lack of established guidelines in defining and documenting nonresponse and treatment failures in general [6, 7]. The treatment of PTSD presents unique challenges that can lead to negative treatment outcomes, such as nonresponse. In particular, complex posttraumatic symptom patterns, comorbid disorders, or various treatment-related or social factors can impede treatment success and lead to nonresponse to trauma-focused treatments [8, 9]. Treatment nonresponse can lead to several severe consequences for the patients, the therapists, and the healthcare system in general. It has been associated with persistent functional impairment and a risk of future relapse for patients [10] and a sense of uncertainty, rejection, and failure for therapists [11, 12]. Nonresponse to treatment can also be a significant financial burden due to prolonged loss of productivity and ongoing healthcare costs [13, 14].

Despite the prevalence of nonresponse and its far-reaching consequences, there are currently no meta-analyses examining the prevalence and predictors of nonresponse to PTSD treatment. Current research suggests that evidence-based first-line psychological treatment approaches leave a substantial subgroup of PTSD patient still suffering from clinically relevant PTSD symptoms posttreatment [6, 15]. Bradley et al. [5] meta-analyzed 26 studies investigating cognitive behavioral therapy (CBT) or eye movement desensitization and reprocessing (EMDR) and found that, across all treatments, 44% of included patients still met criteria for PTSD at posttreatment. Similarly, Schottenbauer et al. [7] found a nonresponse rate of 50% across 55 reviewed studies, whereas Steenkamp et al. [16] revealed even higher rates in military populations, ranging from 50% to 72%. Finally, a more recent meta-analysis across 28 studies on manualized first-line psychological treatment for PTSD found that 41% of participants still met criteria for PTSD at posttreatment, with military populations having higher rates of nonresponse (50%) than civilian populations (35%) [17]. Several variables have been discussed as predictors of nonresponse to PTSD treatment. Regarding demographic variables, older age and male sex have been associated with nonresponse; however, findings remain inconsistent [11, 18, 19]. In addition, PTSD-specific variables, such as PTSD symptom severity, trauma type, and the presence of comorbid disorders, in particular depression, anxiety disorder, and substance use disorders, may influence treatment response. Besides patient variables, certain treatment characteristics may predict treatment nonresponse. Concerning the tolerability of trauma-focused treatment, Dewar et al. [18] reported higher nonresponse rates in studies investigating exposure therapy. However, comparative evidence on the influence of different types of trauma-focused treatment on nonresponse is still lacking. In addition, recent meta-analyses have found no effect of the number of treatment sessions on treatment outcome [19, 20]. Further, current evidence suggests higher efficacy in individual treatment formats and emphasizes the importance of homework adherence for treatment outcomes in trauma-focused treatments [21].

Comprehensive knowledge on the prevalence and predictors of nonresponse is crucial for clinicians' decisions on when to increase the treatment dose [22] or switch to a different treatment approach [23] as well as the development of add-on interventions that could be applied at earlier stages of the treatment [8]. However, there is a significant lack of research investigating nonresponse from first-line guideline-recommended psychological treatments for PTSD. Therefore, the first aim of this study was to determine the prevalence of nonresponse from first-line guideline-recommended psychological treatments for PTSD while considering different operational definitions of the phenomenon. Our second aim was to identify predictors for treatment nonresponse across studies, focusing on study-, patient-, treatment-, and therapist-related variables.

## 2. Method

The protocol was registered in PROSPERO (CRD42023368766), and the meta-analysis is reported according to the Preferred Reporting Items for Systematic Reviews and Meta-Analysis (PRISMA [24]) guideline (for PRISMA checklist, see Supplementary Material A, Table A1, and Table A2).

### 2.1. Identification and Selection of Studies

The inclusion criteria were (a) studies published in English; (b) studies published in a peer-reviewed journal; (c) randomized controlled trials (RCT); (d) studies with participants ≥16 years who (e) all met criteria for a PTSD diagnosis according to DSM-IV, DSM-5, or ICD-10 established with a structured clinical interview; (f) treatment under investigation was a first-line guideline-recommended PTSD treatment [25] and (g) consisted of at least two sessions; (h) data on (non)response (either clinician or self-report) in terms of change in PTSD symptoms were reported separately for each treatment condition; and (i) postassessments were conducted no later than 6 weeks after end of treatment. Studies focusing on patients with severe cognitive impairment, medication trials, placebo-controlled trials, and trials using virtual reality were excluded. These restrictions were applied to reduce the potential heterogeneity among the included studies. The limit of 6 weeks posttreatment was set in order to ensure the inclusion of studies that provided a posttreatment measure and to reduce the variance between postassessment time points. This focus on a postassessment is consistent with the definition of nonresponse provided by Varker et al. [6]. Samples with severe cognitive impairment were excluded due to their difficulties to meet the cognitive demands especially for trauma-focused guideline-recommended PTSD treatment, such as verbal memory or adapting maladaptive patterns. Additionally, psychological treatments delivered exclusively in virtual reality were excluded due to the lack comparability to guideline-recommended treatments delivered in person.

The literature search was conducted using the PTSD Trials Standardized Data Repository [26] (PTSD Repository) containing studies published before July 30, 2021 [27, 28]. The Repository was last updated [29] in the final phase of our work in September 2023, adding studies published before March 3, 2023. In addition, we conducted a database search in Embase, Medline, PsychINFO, and PTSDpubs, using an adapted version of the search string from the PTSD Repository [28]. This was done in order to retrieve all studies published after July 30, 2021, respectively, after March 3, 2023, as well as additional studies not reported in the Repository (see Supplementary Material B for details on both search strategies). The current meta-analysis therefore includes all studies published to October 10, 2023. To determine eligibility, studies were initially examined by two independent reviewers on a title and abstract level (Lea Maria Klöck and Cosima Leithner) and then on a full-text level (Monika Schreckenbach and Lena Ranftl). Any discrepancies were resolved in the whole team.

### 2.2. Data Extraction

Data extraction was independently performed by two researchers (Lea Maria Klöck and Cosima Leithner) using a predefined coding manual. Data extraction was started on January 15, 2023. The mean agreement rate was 99.04% (SD = 1.1%; range, 94.00%–100%). The interrater reliability was calculated as the mean agreement rate across all study agreement scores. The agreement score per study represents the percentage of agreement across all coded items. Any discrepancies were discussed with all members of the team (Verena Semmlinger, Cosima Leithner, Lea Maria Klöck, Lena Ranftl, Thomas Ehring, and Monika Schreckenbach) until a consensus could be reached. First, we extracted data on the number of nonresponders at postassessment for each condition. When results of multiple operationalizations of nonresponse were reported within a study, the operationalization with the highest rank in the following hierarchy was selected: (1) retention of PTSD diagnosis; (2) failure to achieve a predefined symptom reduction (e.g., 10 points or a 30% reduction on the CAPS); (3) failure to achieve significant change according to a statistical formula (e.g., Jacobson and Truax's [30] RCI or clinically significant change); and (4) failure to achieve a predefined cutoff score (e.g., a total score of 20 or less on the CAPS) (for details, see Supplementary Material C). Secondly, we coded available data on study, sample, treatment, and therapist characteristics (see Supplementary Material C, Table C1 for a detailed list of moderators).

### 2.3. Quality Assessment

The risk of bias (RoB) was assessed using Cochrane's RoB 2 tool (for details see Supplementary Material J) [31]. The RoB rating was based on the rating provided in the PTSD Repository, or, for studies not included in the Repository, an additional rating was performed. The assessment included an evaluation of different biases represented by five different domains: randomization process, deviation from intended intervention, missing outcome data, measurement of outcome, and selection of reported results. The overall RoB rating was derived from the ratings within each domain.

### 2.4. Statistical Analysis

#### 2.4.1. Effect Sizes

The primary outcomes were the nonresponse rate and the OR. The nonresponse rate was defined as the proportion of the number of patients who did not respond in a condition out of the total number of patients in that condition. The nonresponse rate was computed separately for each included treatment condition. The OR was calculated as the relative nonresponse rate of a treatment condition compared to a control condition.

We used the Grading of Recommendations, Assessment, Development and Evaluation (GRADE) approach to evaluate the quality of evidence [32]. The *summary of findings* table, generated by using the GRADEpro GDT software [33], provides an overview of the main findings, including an assessment of their quality (see Supplementary Material I for summary of findings). Note that the GRADE approach was developed to assess the quality of evidence regarding effect sizes derived from a comparison between treatments or against a control condition. Due to the specific research question of the current meta-analysis, some GRADE criteria could only be answered with limitations [34].

#### 2.4.2. Multilevel Model

We used multilevel models to estimate average nonresponse rates and ORs as log-transformed proportions or ratios. This was based on the assumption that the true effect size would vary between studies due to the variability between studies. In addition, three-level multilevel models were used due to the nested structure of the data (i.e., several active treatment conditions within a study). The three-level model provided a better fit, i.e., lower AIC and BIC, in comparison to the two-level model (without the study level), AIC = 290.24 versus 304.95, BIC = 298.50 versus 310.46. The R metafor package [35] was used to estimate all multilevel models using the restricted maximum likelihood (REML) estimation. Given the heterogeneity in operationalizations of nonresponse, we conducted an exploratory meta-analysis on a subgroup of studies that operationalized nonresponse as retention of PTSD diagnosis (see Supplementary Material D, with Table D1, Table D2, Figure D1, and Figure D2).

#### 2.4.3. Test of Homogeneity

Cochran's *Q* and *I*^2^ statistics were used to examine heterogeneity in nonresponse rates and ORs. *I*^2^ between 0% and 40% was interpreted as potentially not important, 30%–60% as moderate, 50%–75% as substantial, 75%–90% as substantial to considerable, and >90% as considerable [36, 37].

#### 2.4.4. Subgroup and Metaregression Analyses

The subgroup and metaregression analyses targeted only nonresponse rates, not ORs, as we aimed to identify specific predictors of nonresponse in the treatment conditions. For subgroup analyses, *Q*-statistics served as an omnibus test to identify significant categorical predictors of nonresponse. Metaregression analyses were conducted on continuous predictors (see Supplementary Material C, Table C1). Given the high heterogeneity in included studies, we conducted subgroup and metaregression analyses separately for each predictor. We applied *α*-level corrections using the Benjamini–Hochberg procedure [38] to control for multiple statistical tests.

## 3. Results

### 3.1. Study Characteristics

**Figure 1:**
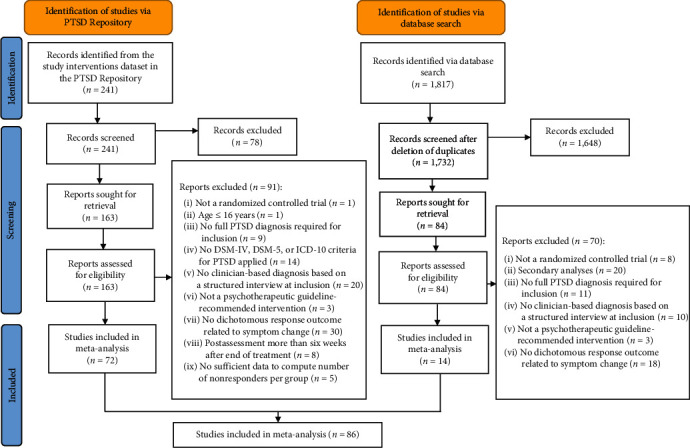
PRISMA flow diagram. *n*, number of studies; PTSD, posttraumatic stress disorder; DSM-IV, Diagnostic and Statistical Manual of Mental Disorders, fourth edition; DSM-5, Diagnostic and Statistical Manual of Mental Disorders, fifth edition; ICD-10, International Classification of Diseases, 10th revision. PRISMA, Preferred Reporting Items for Systematic Reviews and Meta-Analysis. Adapted from the PRISMA 2020 statement: an updated guideline for reporting systematic reviews, 2020 [24].

A total of 86 studies (*k*_*s*_) reporting data of 117 active treatment conditions (*k*_*t*_) and 7,894 participants were included in the meta-analysis (see Figure 1 for the PRISMA flow diagram and Supplementary Material E and Supplementary Material F for included and excluded studies). The majority of studies included were conducted in the USA (*k*_*s*_ = 48) and used retention of PTSD diagnosis to operationalize nonresponse (*k*_*s*_ = 66). Treatment was mostly provided in an individual format (*k_t_ =* 104) and comprised on average 11.3 sessions (SD = 4.6), with *k*_*t*_ *=* 48 treatments delivered by trainee therapists. The weighted mean age of participants was 41.02 years (SD = 6.28) and on average 42.4% (SD = 34.0) of them were female. Most studies used the CAPS (CAPS-IV *k*_*s*_ *=* 44; CAPS-5 *k*_*s*_ *=* 13) for PTSD assessment. Comorbidity was reported in *k*_*t*_ *=* 67 studies, and 61.0% (SD = 15.8%) of participants suffered from comorbid depression (for characteristics of all included studies see Supplementary Material G).

### 3.2. Nonresponse Rate

**Figure 2:**
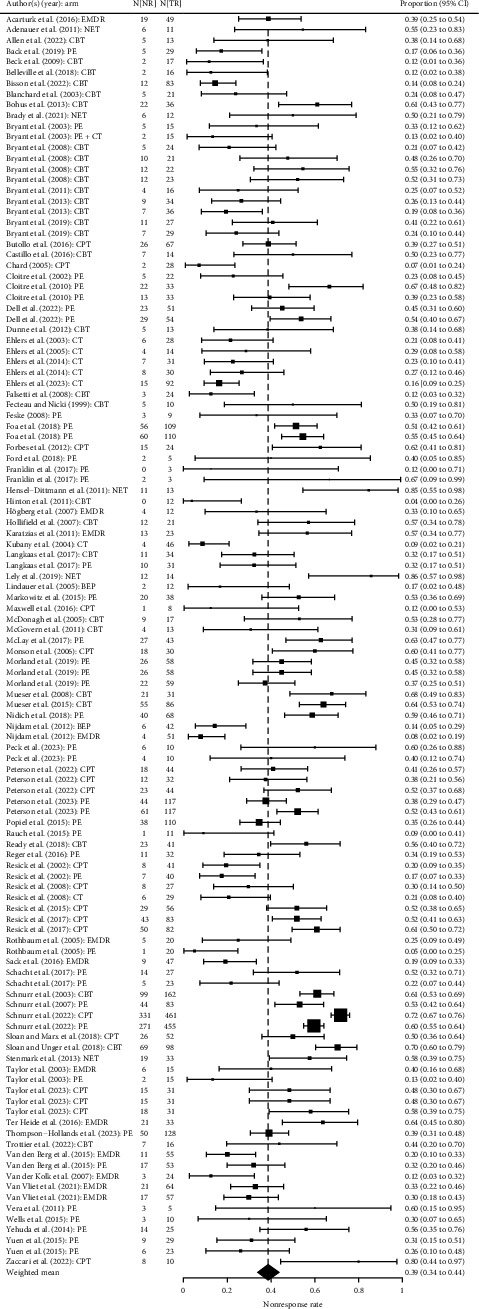
Forest plot of nonresponse rate. N[NR], number of nonresponders; N[TR], number in treatment group; CI, confidence interval; CBT, cognitive behavioral therapy; CPT, cognitive processing therapy; CT, cognitive therapy; PE, prolonged exposure therapy; BEP, brief eclectic therapy; EMDR, eye movement desensitization and reprocessing; NET, narrative exposure therapy. Square size indicates study weight. The zero frequency has been trimmed by adding a small constant for computation purposes.

The weighted average nonresponse rate across all studies and active treatment conditions was 39.23%, 95% CI (35.08%, 43.53%), ranging from 0% to 85.71%. The heterogeneity between studies was rated as *substantial to considerable*, *Q* (116) = 623.30, *p* < 0.0001, *I*^2^ = 83.12%, and 95% CI (81.17, 84.78) (see Figure 2). The pooled OR was 0.22, 95% CI (0.17, 0.26), indicating that nonresponse was less frequent in the treatment condition compared to the control condition. The heterogeneity was *substantial* [36], *Q*(77) = 215.04, *P* < 0.0001, *I*^2^ = 69.80%, and 95% CI (63.74, 74.46) (see Supplementary Material H for a forest plot).

### 3.3. Subgroup Analyses

The nonresponse rate was significantly predicted by the type of analysis, *Q* (1) = 11.21, *p* < 0.001, with lower nonresponse rates in studies reporting per-protocol (PP) analysis (Table 1). The study population also proved to be a significant predictor, *Q* (3) = 29.73, *p* < 0.001, with the lowest nonresponse rates coming from civilian samples. For treatment-related variables, we found the type of intervention to be predictive influence, *Q* (7) = 28.54, *p* < 0.001, with the lowest nonresponse rate for a combination of PE and CT and the highest rate for NET. Treatment format was a further predictor of nonresponse, *Q* (2) = 7.90, *p*=0.019, with the lowest nonresponse for treatments combining individual and group treatment (Table 1). Nonresponse rates were not related to country, in which the study was conducted, method used to operationalize nonresponse, time limit of treatment, homework, and therapist experience.

### 3.4. Metaregression Analyses

Metaregression analyses revealed a significant effect for the year of publication (*p*=.022), with higher nonresponse rates in more recently published studies (Table 2). Furthermore, higher nonresponse rates were found in older samples (*p*=0.002) and in samples with low percentage of female participants (*p*=0.002). In addition, nonresponse was related to PTSD symptom severity (*p*=0.012), such that higher nonresponse rates were associated with higher PTSD symptom severity at baseline. Further, the nonresponse rate was higher in studies with samples where a higher percentage of comorbid depression (*p*=0.005) and higher baseline depression scores (*p* < 0.001) were present. No association with nonresponse was found for marital status, employment status, education level, anxiety score, number of sessions, duration of sessions, or duration of treatment (see Table 2).

### 3.5. Risk of Bias

The overall risk of bias was rated as low for 11 studies (12.8%), 32 studies showed some concerns (37.2%), and the rating was high for 43 studies (50.0%) (for details see Supplementary Material J).

## 4. Discussion

This is, to the best of our knowledge, the first comprehensive meta-analysis on the prevalence and predictors of nonresponse to psychological treatment for PTSD. Across 86 studies investigating first-line guideline-recommended psychological PTSD interventions in a total of 117 active treatment arms, approximately 40% of patients were classified as nonresponders, with a large range from 0% to 85.7%. The OR comparing active treatments with control conditions showed that active interventions considerably reduced the risk for nonresponse compared to control conditions. The prevalence of nonresponse to PTSD treatment found in our meta-analysis is comparable with previous meta-analytic results [5, 17]. These findings show that although guideline-recommended interventions in PTSD are highly efficacious, there is still considerable room for improvement as a substantial subgroup of patients does not respond to treatment.

We identified four groups of significant predictors of treatment nonresponse. First, some demographic and sample characteristics, namely, male sex, older age, and being a refugee or a veteran, were found to be associated with higher nonresponse rates. These findings are in line with previous research; however, note that previous findings have been inconsistent in this regard [11, 18, 39]. Possible explanations for the associations with nonresponse include underlying mechanisms, such as reduced cognitive flexibility in older patients [18], but could also partly be due to confounding variables, such as type of trauma [5, 40]. In particular, combat-related trauma, which is more prevalent among men and refugees and veterans, has been associated with higher nonresponse rates in previous studies [11]. Therefore, future research is needed to examine underlying mechanisms and the unique effect of trauma type on nonresponse. Second, two aspects of baseline psychopathology were found to be significant predictors of nonresponse, including high PTSD symptom severity. Further research is needed to replicate our findings regarding the potential impact of high PTSD symptom severity on treatment outcomes, as previous findings have been inconsistent [18]. Within these new approaches, it is important to consider the potential underlying interference of reduced engagement due to high avoidance tendencies in patients with severe PTSD [19, 41]. In addition to PTSD severity, having a comorbid depressive disorder or elevated depressive symptoms at baseline was also found to be predictive of treatment nonresponse. It is conceivable that the reduced emotional activation prevalent in patients with depression may interfere with traumatic memory modification in trauma-focused PTSD treatment [42, 43]. In addition, other possible mechanisms, such as rumination, avoidance, numbing, anhedonia, or diminished reward processing, may explain the interference of comorbid depression with trauma processing [44, 45, 46]. Therefore, more research is needed to identify possible mechanisms.

Third, certain treatment characteristics were predictive of nonresponse. Treatment type significantly predicted nonresponse, with the most frequently studied psychological interventions for PTSD, such as CBT, CPT, and PE all showing comparable nonresponse rates (CBT, 40.73%; CPT, 47.52%; and PE, 39.60%), with slightly lower rates reported in EMDR studies (31.71%). The lowest nonresponse was found in studies combining PE and CT (14.98%) and the highest in studies evaluating NET (65.85%). These findings need to be treated with caution since we were able to include only one study with PE + CT and only five investigating NET. Furthermore, possible confounding variables (e.g., sample characteristics) cannot be ruled out, requiring closer investigation with a larger number of studies. Besides replicating our findings on treatment type in a larger sample, future research should focus on comparing trauma-focused treatment with nontrauma focused approaches. In addition, we found the combination of individual and group therapy to have a considerably lower rate (7.14%) of nonresponse when compared to individual (38.28%) or group therapy (49.13%) alone. However, only one study investigated the combined treatment category. Therefore, future research is needed to determine, whether the findings can be replicated in a larger sample. Fourth, we found that nonresponse was significantly higher in studies reporting intention-to-treat (ITT) (44.90%) than per-protocol (PP) (31.06%) [5]. This was to be expected as higher rates of nonresponse are more likely to occur with patients who do not complete the whole course of treatment.

Interestingly, the prevalence of nonresponse was not associated with the type of operationalizing nonresponse. This implies that the classification of a patient as (non)responder was unlikely to change when applying different operationalizations. Nevertheless, the present findings indicate the need to develop guidelines on defining and operationalizing nonresponse. Future research should focus on the empirical validation of different operationalization methods and the adaptation of these criteria to different assessment tools and different versions of one assessment tool [6, 47]. In addition, it is important to develop a comparable operationalization method that can be applied across assessment tools. This could be achieved by defining a failure to achieve a reduction of a certain percentage on a scale's range as indicative of nonresponse, whereby the percentage has been validated across scales [47]. Furthermore, research should consider the combination of different criteria. For example, when nonresponse is operationalized as retaining the diagnosis, patients with higher baseline symptom severities are more likely to be classified as nonresponders at posttreatment, even in cases where they show the largest symptom reduction. Therefore, combining retention of diagnosis with indicators for magnitude of the treatment effect appears informative. Relatedly, it appears recommendable to include not only indicators of nonresponse regarding PTSD symptomology but additionally functional outcomes and individual patient goals. Patients often perceive quality of life and functioning as more crucial and meaningful than symptom relief [48]. Recent research has shown that the best improvement of functional outcomes and quality of life can in fact be reached when patients are treated to remission [49]. Further, it is important to consider the long-term effects of treatment and therefore the time point of assessment when operationalizing nonresponse [50]. In line with the recommendations of Varker et al. [6], we included only studies providing a postassessment within 6 weeks of treatment completion. While a pre–post comparison is recommended, it may be promising to additionally consider follow-up measures when conceptualizing nonresponse, especially in the light of the current evidence indicating ongoing improvement following the termination of treatment [51, 52].

In addition to the implications for further research that can be derived directly from the findings of our study, it is important to consider additional factors that could influence treatment response in future research. Besides cognitive factors, verbal memory could be investigated as a means of expanding the understanding of underlying mechanisms [53]. Additional variables of interest include social support [54], physical health [55], and other comorbid disorders such as sleep disorders [56], alcohol and substance use disorder [57, 58], and borderline personality disorder [59].

Our findings have potentially important implications for clinical practice. To increase treatment efficacy, it seems necessary to modify first-line guideline-recommended treatment approaches for different subgroups of PTSD patients, characterized by one or more of the identified baseline predictor variables [25, 60]. Extending treatment for a longer period of time has previously been discussed as a promising approach for patients being at higher risk for nonresponse due to the high PTSD symptom severity [61, 62]. This approach is often implemented in routine clinical practice [25]. However, our analysis did not find therapy dose (e.g., number of sessions, treatment duration, and session duration) to be a significant predictor of nonresponse. Therefore, additional measures should be considered, including starting treatment immediately without considerable waiting time [63] or offering a higher session frequency early on [64, 65]. In addition, our findings suggest the importance of considering comorbidity, particularly comorbid depression, in PTSD treatment. Although evidence shows that depressive symptoms improve with successful PTSD treatment [66], our results suggest that high levels of depressive symptoms may need more attention in treatment planning. Specifically, targeting excessively high depressive symptoms before engaging in trauma-focused interventions may be recommendable [60]. Further, adjuvant and second-line therapies may offer an alternative for patients at high risk of nonresponse [8]. These include novel psychotherapeutic approaches, such as imaginal rehearsal therapy, as well as pharmacological interventions or neuromodulatory approaches [8, 67].

### 4.1. Strengths and Limitations

Our meta-analysis had several strengths. We applied strict inclusion criteria, e.g., exclusively focusing on RCTs investigating first-line evidence-based guideline-recommended psychological interventions. Furthermore, patients were diagnosed with PTSD using structured clinician-administrated interviews to minimize sample heterogeneity. In addition, a large number of studies were included, enhancing the reliability and generalizability of our findings. Finally, the interrater reliability was very high at all stages of study selection and coding. A number of limitations are noteworthy. Firstly, the operationalization of predictors varied widely between studies. This forced us to test variables separately, as simultaneously entering multiple predictors into a metaregression model would have reduced the number of studies in the analysis. Secondly, some potentially important predictors, such as trauma history, the use of psychotropic medication, the number of trauma-focused sessions [68], the duration of exposure periods [8], and therapeutic alliance [69], could not be coded and included in the analyses due to a lack of data reported in the original studies and poor data quality. Thirdly, none of the studies investigated complex PTSD (cPTSD) as defined by ICD-11 [70]. Since it is estimated that around 30%–50% of PTSD patients fulfill criteria for cPTSD [71, 72], the samples included in the current analysis most likely also included certain numbers of cPTSD patients. Future research should focus on nonresponse with respect to cPTSD since childhood-onset of trauma has been found to be reliably associated with both cPTSD and poorer treatment outcomes [73]. Finally, the generalizability of the findings to a wider context and cultural setting needs to be considered. The majority of included studies were conducted in high-income countries. Although the sample of studies is not representative of the global population, it provides a valid representation of the current state of research on PTSD treatment. Therefore, our results highlight the lack of research in diverse cultural contexts. It is evident that further research is needed to derive specific implications for the clinical practice.

### 4.2. Conclusions

In this comprehensive meta-analysis, we found that a substantial subgroup of patients suffering from PTSD still showed clinically significant symptoms after having received a first-line guideline-recommended treatment for PTSD. Thus, although these interventions are very efficacious on the group level, a considerable number of patients do not sufficiently benefit. Investigating predictors of nonresponse may help to understand and prevent these high rates. In our meta-analysis, males, older individuals, and veterans and refugees were at greater risk of treatment nonresponse. Furthermore, symptom severity at baseline, specifically higher PTSD symptom severity as well as comorbid depression, was associated with nonresponse. In addition, certain treatment characteristics were found to be predictive of nonresponse, namely, treatment type and treatment format. Future research is needed to replicate our findings, identify underlying mechanisms and potential confounding variables, and examine the influence of additional predictor variables on nonresponse. In conclusion, our findings have important implications for clinical practice. To reduce nonresponse, it seems necessary to modify guideline-recommended treatment approaches for patients at high risk of nonresponse based on identified baseline predictors. Additionally, second-line treatment options may be advisable for this specific subgroup of patients.

## Figures and Tables

**Table 1:** Results from subgroup analyses on the nonresponse rate.

Moderator (*k*_*t*_)	NR (%)	95% CI	*Q*	*p*	Adj. *α*
Study characteristics					
Country of study (117)	—	—	9.36	0.589	0.044
USA (66)	41.81	(36.21, 47.63)			
Australia (15)	38.54	(26.47, 52.20)
Netherlands (9)	34.00	(20.95, 50.02)
Germany (5)	48.09	(30.44, 66.23)
Canada (5)	32.67	(16.49, 54.38)
England (8)	23.94	(14.36, 37.14)
Norway (4)	41.02	(21.53, 63.81)
Poland (1)	34.55	(10.84, 69.61)
Puerto Rico (1)	60.00	(13.29, 93.62)
Scotland (1)	56.52	(20.19, 86.98)
Thailand (1)	25.00	(5.17, 67.10)
Turkey (1)	38.78	(12.10, 74.46)
Type of analysis (117)	—	—	11.21	<0.001*⁣*^*∗∗*^	0.017
Per protocol (49)	31.06	(25.55, 37.16)			
Intention to treat (68)	44.90	(39.65, 50.26)
Operationalization of NR (117)	—	—	2.81	0.422	0.039
Retention of diagnosis (93)	37.45	(32.89, 42.24)			
Symptom reduction (12)	47.77	(35.41, 60.40)
Significant change (9)	41.59	(27.52, 57.18)
Cutoff score (3)	46.85	(24.97, 70.01)
Sample characteristics					
Population (117)	—	—	29.73	<0.001*⁣*^*∗∗*^	0.006
Civil (71)	31.42	(27.28, 35.87)			
Veterans and Military Personnel (40)	50.66	(44.35, 56.94)
Refugee (5)	57.84	(41.60, 72.54)
Mixed (1)	50.00	(21.76, 78.24)
Treatment characteristics					
Type of intervention (117)	—	—	28.54	<0.001*⁣*^*∗∗*^	0.011
PE (41)	39.60	(33.88, 47.95)			
CBT (30)	40.73	(33.54, 47.99)
CPT (19)	47.52	(39.50, 55.67)
EMDR (12)	31.71	(23.48, 41.27)
CT (7)	20.99	(13.01, 32.06)
NET (5)	65.85	(47.68, 80.31)
BEP (2)	24.82	(10.37, 48.52)
PE + CT (1)	14.98	(3.00, 50.06)
Treatment format (116)	—	—	7.90	0.019^*∗*^	0.022
Individual (104)	38.28	(34.11, 42.63)			
Group (11)	49.13	(38.12, 60.23)
Combined (1)	7.14	(1.06, 35.65)
Time limit (115)	—	—	1.34	0.247	0.033
Low (≤12 sessions) (76)	37.94	(33.00, 43.14)			
High (>12 sessions) (39)	42.99	(36.00, 50.27)
Homework given (117)	—	—	0.07	0.795	0.05
Yes (77)	39.60	(34.58, 44.84)			
No (40)	38.47	(31.75, 45.67)
Therapist characteristics					
Therapist experience level (92)	—	—	6.05	0.109	0.028
Trainee (48)	37.29	(31.24, 43.77)			
Experienced (16)	27.42	(18.81, 38.11)			
Mixed (18)	43.18	(32.73, 54.27)			
Nonprofessionals^a^ (10)	46.47	(32.60, 60.90)			

*k*_*t*_, number of treatment conditions; *Q*, Cochrane's *Q*; CI, confidence interval; adj. *α*, adjusted *α* level after Benjamini–Hochberg approach; NR, nonresponse rate; symptom reduction, nonachievement of predefined symptom reduction; significant change, nonachievement of significant change per statistical formula; cutoff score, nonachievement of predefined cutoff score; PTSD, posttraumatic stress disorder; CBT, cognitive behavioral therapy; CPT, cognitive processing therapy; CT, cognitive therapy; PE, prolonged exposure therapy; BEP, brief eclectic therapy; EMDR, eye movement desensitization and reprocessing; NET, narrative exposure therapy. ^a^Nonprofessional refers to treatment provided not by therapists, e.g., social worker. *⁣*^*∗*^Benjamini–Hochbergcorrected *p* < 0.05. *⁣*^*∗∗*^Benjamini–Hochberg corrected *p* < 0.01.

**Table 2:** Results from metaregression analyses on the nonresponse rate (log-transformed).

Moderator (*k*_*t*_)	*β*	95% CI	*p*	Adj. *α*
Study characteristics				
Year of study publication (117)	0.03	(0.01, 0.06)	0.022^*∗*^	0.023
Sample characteristics				
Age (80)	0.05	(0.02, 0.07)	0.002^*∗∗*^	0.008
Sex (85): female (%)	−0.87	(−1.43, −0.31)	0.002^*∗∗*^	0.012
Marital (53): committed relationship (%)	0.97	(−0.12, 2.06)	0.080	0.027
Employment (42): employed (%)	−0.36	(−1.40, 0.67)	0.492	0.042
Education (40): college level (%)	0.42	(−0.76, 1.60)	0.485	0.046
PTSD symptom severity score (88)^a^	0.24	(0.05, 0.43)	0.012^*∗*^	0.019
Comorbid depression (40): diagnosis (%)	2.87	(0.88, 4.86)	0.005^*∗∗*^	0.015
Depression score (87)^a^	0.39	(0.19, 0.59)	<0.001*⁣*^*∗∗*^	0.004
Anxiety score (48)^a^	0.21	(−0.07, 0.48)	0.145	0.031
Treatment characteristics				
Number of sessions (116)	0.02	(−0.01, 0.05)	0.238	0.035
Duration of session in minutes (103)	0.00	(−0.01, 0.01)	0.438	0.038
Duration of treatment in weeks (102)	0.01	(−0.02, 0.04)	0.573	0.05

*k*_*t*_, number of treatment conditions; CI, confidence interval, adj. *α*, adjusted *α* level after Benjamini–Hochberg approach; regression models were estimated separately for each predictor; ^a^*z*-standardized. *⁣*^*∗*^Benjamini-Hochberg corrected *p* < 0.05. *⁣*^*∗∗*^Benjamini–Hochberg corrected *p* < 0.01.

## Data Availability

All presented data are publicly accessible. The data and analytic code that support the findings of this study are openly available on OSF: https://osf.io/kvxbw/?view_only=9fb34187caff4c8a81549fc9ac197625. Data will be made available immediately following publication with no end date. There are no access limits.

## Supplementary Materials

Supplementary Materials Additional information can be found in the Supplementary Material. The Supplementary Material includes detailed information on the search strategy and the data extraction process. Results of additional analyses and a full reference list of included and excluded studies are provided. In addition, the Supplementary Material contains a full table of included variables, the forest plot for the OR, and detailed results of the GRADE and risk of bias assessments.
