# Supplementary material for: Prevalence and Predictors of Nonresponse to Psychological Treatment for PTSD: A Meta-Analysis
Source: Depress Anxiety. 2024 Jul 26;2024:9899034. doi: 10.1155/2024/9899034 (PMC11918500; doi:10.1155/2024/9899034)
Supplement: Supplementary Materials — Additional information can be found in the Supplementary Material. The Supplementary Material includes detailed information on the search strategy and the data extraction process. Results of additional analyses and a full reference list of included and excluded studies are provided. In addition, the Supplementary Material contains a full table of included variables, the forest plot for the OR, and detailed results of the GRADE and risk of bias assessments. [file 9899034.f1.pdf]

Supplementary Material

A. PRISMA Checklists 2
B. Search Strategy 5
C. Data Extraction – Variables 6
D. Results of Sub Meta-analysis 7
E. References included in the meta-analysis 12
F. List of excluded studies 18
G. Full Table of included studies and variables 31
H. Forest Plot of *OR* 51
I. GRADE: Summary of Findings 52
J. Risk of Bias Assessment 54

A. PRISMA Checklists

Table A1 PRISMA 2020 Checklist

Section and Topic	Item #	Checklist item	Location where item is reported
**TITLE**			
Title	1	Identify the report as a meta-analysis	Page 1
**ABSTRACT**			
Abstract	2	See the PRISMA 2020 for Abstracts checklist.	See Abstract Checklist
**INTRODUCTION**			
Rationale	3	Describe the rationale for the review in the context of existing knowledge.	Page 1-2
Objectives	4	Provide an explicit statement of the objective(s) or question(s) the review addresses.	Page 2; Abstract
**METHODS**			
Eligibility criteria	5	Specify the inclusion and exclusion criteria for the review and how studies were grouped for the syntheses.	Page 2-3
Information sources	6	Specify all databases, registers, websites, organisations, reference lists and other sources searched or consulted to identify studies. Specify the date when each source was last searched or consulted.	Page 2; 4; B
Search strategy	7	Present the full search strategies for all databases, registers and websites, including any filters and limits used.	Page 2; B
Selection process	8	Specify the methods used to decide whether a study met the inclusion criteria of the review, including how many reviewers screened each record and each report retrieved, whether they worked independently, and if applicable, details of automation tools used in the process.	Page 2-3
Data collection process	9	Specify the methods used to collect data from reports, including how many reviewers collected data from each report, whether they worked independently, any processes for obtaining or confirming data from study investigators, and if applicable, details of automation tools used in the process.	Page 2-3; C
Data items	10a	List and define all outcomes for which data were sought. Specify whether all results that were compatible with each outcome domain in each study were sought (e.g. for all measures, time points, analyses), and if not, the methods used to decide which results to collect.	Page 3; C; G
	10b	List and define all other variables for which data were sought (e.g. participant and intervention characteristics, funding sources). Describe any assumptions made about any missing or unclear information.	Page 3; C; G
Study risk of bias assessment	11	Specify the methods used to assess risk of bias in the included studies, including details of the tool(s) used, how many reviewers assessed each study and whether they worked independently, and if applicable, details of automation tools used in the process.	Page 3, J
Effect measures	12	Specify for each outcome the effect measure(s) (e.g. risk ratio, mean difference) used in the synthesis or presentation of results.	Page 3
Synthesis methods	13a	Describe the processes used to decide which studies were eligible for each synthesis (e.g. tabulating the study intervention characteristics and comparing against the planned groups for each synthesis (item #5)).	Page 3
	13b	Describe any methods required to prepare the data for presentation or synthesis, such as handling of missing summary statistics, or data conversions.	Page 3
	13c	Describe any methods used to tabulate or visually display results of individual studies and syntheses.	Page 3
	13d	Describe any methods used to synthesize results and provide a rationale for the choice(s). If meta-analysis was performed, describe the model(s), method(s) to identify the presence and extent of statistical heterogeneity, and software package(s) used.	Page 3
	13e	Describe any methods used to explore possible causes of heterogeneity among study results (e.g. subgroup analysis, meta-regression).	Page 3
	13f	Describe any sensitivity analyses conducted to assess robustness of the synthesized results.	/
Reporting bias assessment	14	Describe any methods used to assess risk of bias due to missing results in a synthesis (arising from reporting biases).	Page 3, J
Certainty assessment	15	Describe any methods used to assess certainty (or confidence) in the body of evidence for an outcome.	Page 3, I

Section and Topic	Item #	Checklist item	Location where item is reported
**RESULTS**			
Study selection	16a	Describe the results of the search and selection process, from the number of records identified in the search to the number of studies included in the review, ideally using a flow diagram.	PRISMA flow diagram; Page 4
	16b	Cite studies that might appear to meet the inclusion criteria, but which were excluded, and explain why they were excluded.	F
Study characteristics	17	Cite each included study and present its characteristics.	Page 3-4; E; G
Risk of bias in studies	18	Present assessments of risk of bias for each included study.	Page 4; 7, J
Results of individual studies	19	For all outcomes, present, for each study: (a) summary statistics for each group (where appropriate) and (b) an effect estimate and its precision (e.g. confidence/credible interval), ideally using structured tables or plots.	Page 4-7; Table 1, Table 2; Fig. 2; D, J
Results of syntheses	20a	For each synthesis, briefly summarise the characteristics and risk of bias among contributing studies.	Page 4-7; Table 1; Table 2; J
	20b	Present results of all statistical syntheses conducted. If meta-analysis was done, present for each the summary estimate and its precision (e.g. confidence/credible interval) and measures of statistical heterogeneity. If comparing groups, describe the direction of the effect.	Page 4-7; Table 1; Table 2; Fig. 2; D; J
	20c	Present results of all investigations of possible causes of heterogeneity among study results.	Page 4-7; Table 1; Table 2
	20d	Present results of all sensitivity analyses conducted to assess the robustness of the synthesized results.	/
Reporting biases	21	Present assessments of risk of bias due to missing results (arising from reporting biases) for each synthesis assessed.	Page 4; 7, J
Certainty of evidence	22	Present assessments of certainty (or confidence) in the body of evidence for each outcome assessed.	Page 4-7; Table 1; Table 2; Fig. 2; J
**DISCUSSION**			
Discussion	23a	Provide a general interpretation of the results in the context of other evidence.	Page 7-9
	23b	Discuss any limitations of the evidence included in the review.	Page 9
	23c	Discuss any limitations of the review processes used.	Page 9
	23d	Discuss implications of the results for practice, policy, and future research.	Page 8-9
**OTHER INFORMATION**			
Registration and protocol	24a	Provide registration information for the review, including register name and registration number, or state that the review was not registered.	Page 1; 2
	24b	Indicate where the review protocol can be accessed, or state that a protocol was not prepared.	Page 1; 2
	24c	Describe and explain any amendments to information provided at registration or in the protocol.	n.a.
Support	25	Describe sources of financial or non-financial support for the review, and the role of the funders or sponsors in the review.	Page 9
Competing interests	26	Declare any competing interests of review authors.	Page 9
Availability of data, code and other materials	27	Report which of the following are publicly available and where they can be found: template data collection forms; data extracted from included studies; data used for all analyses; analytic code; any other materials used in the review.	Page 9

From: Page MJ, McKenzie JE, Bossuyt PM, Boutron I, Hoffmann TC, Mulrow CD, et al. The PRISMA 2020 statement: an updated guideline for reporting systematic reviews. BMJ 2021;372:n71. doi: 10.1136/bmj.n71 For more information, visit: http://www.prisma-statement.org/

Table A2 PRISMA 2020 for Abstracts Checklist

Section and Topic	Item #	Checklist item	Reported (Yes/No)
**TITLE**			
Title	1	Identify the report as a meta-analysis.	Yes
**BACKGROUND**			
Objectives	2	Provide an explicit statement of the main objective(s) or question(s) the review addresses.	Yes
**METHODS**			
Eligibility criteria	3	Specify the inclusion and exclusion criteria for the review.	Yes
Information sources	4	Specify the information sources (e.g. databases, registers) used to identify studies and the date when each was last searched.	Yes
Risk of bias	5	Specify the methods used to assess risk of bias in the included studies.	Yes
Synthesis of results	6	Specify the methods used to present and synthesise results.	Yes
**RESULTS**			
Included studies	7	Give the total number of included studies and participants and summarise relevant characteristics of studies.	Yes
Synthesis of results	8	Present results for main outcomes, preferably indicating the number of included studies and participants for each. If meta-analysis was done, report the summary estimate and confidence/credible interval. If comparing groups, indicate the direction of the effect (i.e. which group is favoured).	Yes
**DISCUSSION**			
Limitations of evidence	9	Provide a brief summary of the limitations of the evidence included in the review (e.g. study risk of bias, inconsistency and imprecision).	No
Interpretation	10	Provide a general interpretation of the results and important implications.	Yes
**OTHER**			
Funding	11	Specify the primary source of funding for the review.	No
Registration	12	Provide the register name and registration number.	Yes

From: Page MJ, McKenzie JE, Bossuyt PM, Boutron I, Hoffmann TC, Mulrow CD, et al. The PRISMA 2020 statement: an updated guideline for reporting systematic reviews. BMJ 2021;372:n71. doi: 10.1136/bmj.n71 For more information, visit: http://www.prisma-statement.org/

B. Search Strategy

The literature search consisted of two independent search strategies covering the full scope of the published studies.

First Search Strategy: PTSD Trials Standardized Data Repository (PTSD Repository)

The PTSD Repository is a large database currently comprising 437 RCTs studying treatment for PTSD published between 1988 and March 03, 2023. We searched the *Study Interventions* data set for relevant studies, filtering the *Study Class* column for *Psychotherapy*. All studies were retrieved using the *Citation* column, and saved for further selection and screening. The PTSD Repository was only used to identify relevant studies. We did not use the data extracted from the relevant studies and provided in the PTSD Repository, but extracted the relevant information from the selected studies on our own.

Second Search Strategy: Database Search

The database search was conducted to retrieve all studies not included in the PTSD Repository. At the beginning of our work, the PTSD Repository contained all studies published up to July, 30, 2021 (update of the PTSD Repository published September, 19, 2022). At the time of finalization of our work, there was a second update published on September, 2023 containing all studies published up to March 03, 2023. We used an adapted version of the search string used in the PTSD-Repository. We searched the following electronic databases: Ovid Embase, Ovid Medline, PsycINFO (via Ebscohost), PTSDpubs. The following adaptions to the search sting from PTSD Repository had to be made: (1) Embase retrieved via Ovid, translation of search string for the provider; (2) PsycINFO retrieved via Ebscohost, translation of search string for the provider, (3) Cochrane CENTRAL, CINAHL, and SCOPUS were not included. The initial database search was conducted on April 12,2023 and included all studies published after June 01, 2021 (overlap with the PTSD Repository database). To updates were realized on July 20, 2023 and October 10,2023.

C. Data Extraction – Variables

Non-Response

The non-response rate for each condition was calculated from (a) the number of non-responders at post-assessment for each condition (as a numerator) and (b) the number of participants who were randomized to that respective condition (as a denominator).
The extraction of the number of non-responders followed specific conditions:

(1) data were extracted at the respective post-assessment time point (≤ 6 weeks after treatment);
(2) the number of non-responders was directly extracted from the respective study (when reported), otherwise the number of non-responders was calculated by subtracting the reported number of responders in a condition from the total number of participants in that condition;
(3) non-response information based on per-protocol (PP) data was preferred, otherwise information was extracted from the intention-to-treat (ITT) sample
(4) a hierarchical structure was applied in studies, that reported different operationalization methods of non-response. Non-response data on the highest rank reported were extracted). If multiple data out of the same category were reported, we extracted the non-response information on the least stringent operationalization method given.
    a. retention of PTSD diagnosis
    b. failure to achieve a predefined symptom reduction as defined by the authors (e.g., 10-point/ 30% reduction on CAPS)
    c. failure to reach a significant change (e.g., RCI or clinically significant change)
    d. failure to achieve a predefined cut-off score (e.g., CAPS total score below 20)

Table C1 List of Variables and Operationalization

Domain	Variable	Operationalization
Study characteristics	Year of publication	Years
	Country of study	Country in which the study was conducted
	Sample size	*N*
	Type of control condition	Waiting list / Treatment as usual (TAU) / active control
	Type of analysis	Per protocol (PP) / intention to treat (ITT)
	Operationalization of non-response	Retention of PTSD-diagnosis / non-achievement of predefined symptom reduction / non-achievement of significant change / non-achievement of predefined cut-off score
Sample characteristics	Age	Mean age
	Sex	Percent female
	Marital Status	Percent married or in committed relationships
	Employment status	Percent employed full-time or part-time or student
	Education	Percent with college-level education
	Population	Civil / refugees/ Veterans & Military Personnel / mixed
	PTSD symptom severity	Z-standardized mean (CAPS, PSS-I, PCL)
	Comorbid depression	Percent with comorbid depression diagnosis
	Depression severity	Z-standardized mean (BDI, PHQ-9. HDRS, HRSD HAM-D, HADS, DASS, HSCL-25)
	Anxiety severity	Z-standardized mean (STAI, BAI, HSCL-25, DASS, HADS, SCL, STAX-trait)
Treatment-related variables	Treatment orientation	PE / CPT / CBT / EMDR / CT / BEP / NET
	Treatment format	Individual / group / combined
	Predefined time limit of treatment[1]	≤12 sessions / >12 sessions
	Number of sessions	Mean
	Duration of treatment in weeks	Mean
	Planned duration of sessions in minutes	Mean
	Homework given	Yes / no
Therapist characteristics	Experience level	Trainee / experienced/ mixed/ non-professionals

[1] For the time limit, 12 sessions were set as cut-off, as this reflects the average of the suggested number of sessions for the seven from the APA recommended guideline-recommended PTSD treatments.

D. Results of Sub Meta-analysis

Non-Response Rate

The weighted average non-response in the sub meta-analysis was 37.37%, 95% CI [32.54%, 42.46%], range 0% to 85.71%. The heterogeneity between studies was *substantial to considerable*, $Q(92) = 562.53$, $p < .0001$, $I^2 = 84.93\%$, 95% CI [83.07, 86.49]. The pooled *OR* in the sub meta-analysis was *OR* = 0.19, 95% CI [0.15, 0.25], with a *substantial* heterogeneity $Q(61) = 165.89$, $p < .0001$, $I^2 = 68.52\%$, 95% CI [61.10, 74.00].
The non-response rate and OR of the sub-meta-analysis were comparable to the full meta-analysis.

Subgroup analyses

All results are comparable to the full meta-analysis, expect for the predictor treatment format that no longer was a significant predictor for non-response in the sub meta-analysis.

Table D1 Results from Subgroup Analyses on the Non-Response Rate (Sub Meta-Analysis)

Moderator (k_t)	NR rate (%)	95% CI	*Q*	*p*	Adj. *α*
Study characteristics					
Country of study (93)			7.00	.638	.044
USA (48)	39.86	[32.91–47.25]			
Australia (15)	38.53	[25.74–53.13]			
Netherlands (9)	34.27	[20.43–51.43]			
Germany (4)	44.69	[24.62–66.64]			
Canada (5)	32.58	[15.80–55.45]			
England (7)	21.25	[11.68–35.52]			
Norway (2)	46.98	[19.92–75.94]			
Poland (1)	34.55	[9.81–71.91]			
Puerto Rico (1)	60.00	[12.47–94.04]			
Turkey (1)	38.78	[11.00–76.44]			
Type of analysis (93)			5.91	.015**	.019
Per protocol (38)	30.26	[23.78, 37.64]			
Intention to treat (55)	42.26	[36.02, 48.75]			
Sample characteristics					
Population (93)			28.42	< .001**	.006
Civil (58)	29.12	[24.66, 34.03]			
Veterans & Military Personnel (29)	50.94	[42.99, 58.84]			
Refugee (5)	57.97	[41.34, 72.94]			
Mixed (1)	50.00	[21.13, 78.87]			
Treatment characteristics					
Type of intervention (93)			27.96	<.001**	.013
PE (31)	38.00	[31.39, 45.08]			
CBT (23)	37.91	[29.80, 46.76]			
CPT (14)	47.26	[38.34, 56.37]			
EMDR (11)	29.60	[21.25, 39.59]			
CT (7)	21.15	[12.93, 32.62]			
NET (4)	69.75	[49.30, 84.54]			
BEP (2)	24.85	[10.26, 48.89]			
PE + CT (1)	14.63	[2.90, 49.59]			
Treatment format (92)			5.40	.067	.025
Individual (85)	36.90	[32.04, 42.03]			
Group (6)	46.37	[32.15, 61.21]			
Combined (1)	7.14	[0.98, 37.52]			
Time limit (10)			0.68	.410	.038
Low (≤ 12 sessions) (60)	36.35	[30.51, 42.63]			
High (> 12 sessions) (31)	40.51	[32.53, 49.01]			
Homework given (93)			0.06	.804	.05
Yes (60)	36.59	[29.13, 44.74]			
No (33)	37.81	[31.90, 44.10]			
Therapist characteristics					
Therapist experience level (78)			6.83	.078	.031
Trainee (40)	35.22	[28.52, 42.57]			
Experienced (15)	25.11	[16.61, 36.09]			
Mixed (16)	41.80	[30.53, 54.00]			
Non-professionals (7)	48.35	[31.81, 65.25]			

k_t = number of treatment conditions; *Q* = Cochrane's *Q*; CI = confidence interval; adj. α = adjusted α level after Benjamini–Hochberg approach; NR = non-response; PTSD = posttraumatic stress disorder; CBT = cognitive behavioral therapy; CPT = cognitive processing therapy; CT = cognitive therapy; PE = prolonged exposure therapy; BEP = brief eclectic therapy; EMDR = eye movement desensitization and reprocessing; NET = narrative exposure therapy.
*Benjamini–Hochberg corrected $p < .05$. **Benjamini–Hochberg corrected $p < .01$.

Meta-regression analyses

All results are comparable to the full meta-analysis.

Table D2. Results from Meta-Regression Analyses on the Non-Response Rate (Sub Meta-Analysis; Log-Transformed)

**Moderator (k_t)**	**β**	**95% CI**	***p***	**Adj. α**
Study characteristics				
Year of study publication (93)	0.04	[0.01, 0.07]	.018*	.023
Sample characteristics				
Age (63)	0.07	[0.03, 0.10]	<.001**	.004
Sex (66): % female	-0.95	[-1.68, -0.22]	.011*	.015
Marital (40): % committed relationship	1.27	[-0.23, 2.76]	.097	.027
Employment (32): % employed	-0.72	[-2.09, 0.66]	.306	.035
Education (32): % college-level	0.16	[-1.27, 1.59]	.829	.05
PTSD symptom severity score (70) [a]	0.27	[0.06, 0.48]	.013*	.019
Comorbid depression (35): % diagnosis	3.10	[1.07, 5.12]	.003**	.012
Depression score (75) [a]	0.42	[0.19, 0.65]	< .001**	.008
Anxiety score (43) [a]	0.25	[-0.05, 0.55]	.106	.031
Treatment characteristics				
Number of sessions (92)	0.01	[-0.04, 0.06]	.741	.046
Duration of session in minutes (79)	0.00	[-0.01, 0.01]	.732	.042
Duration of treatment in weeks (81)	-0.01	[-0.05, 0.03]	.581	.038

k_t = number of treatment conditions, CI = confidence interval, adj. α = adjusted α level after Benjamini–Hochberg approach, regression models were estimated separately for each predictor; [a] *z*-standardized
*Benjamini-Hochberg corrected $p < .05$. **Benjamini-Hochberg corrected $p < .01$.

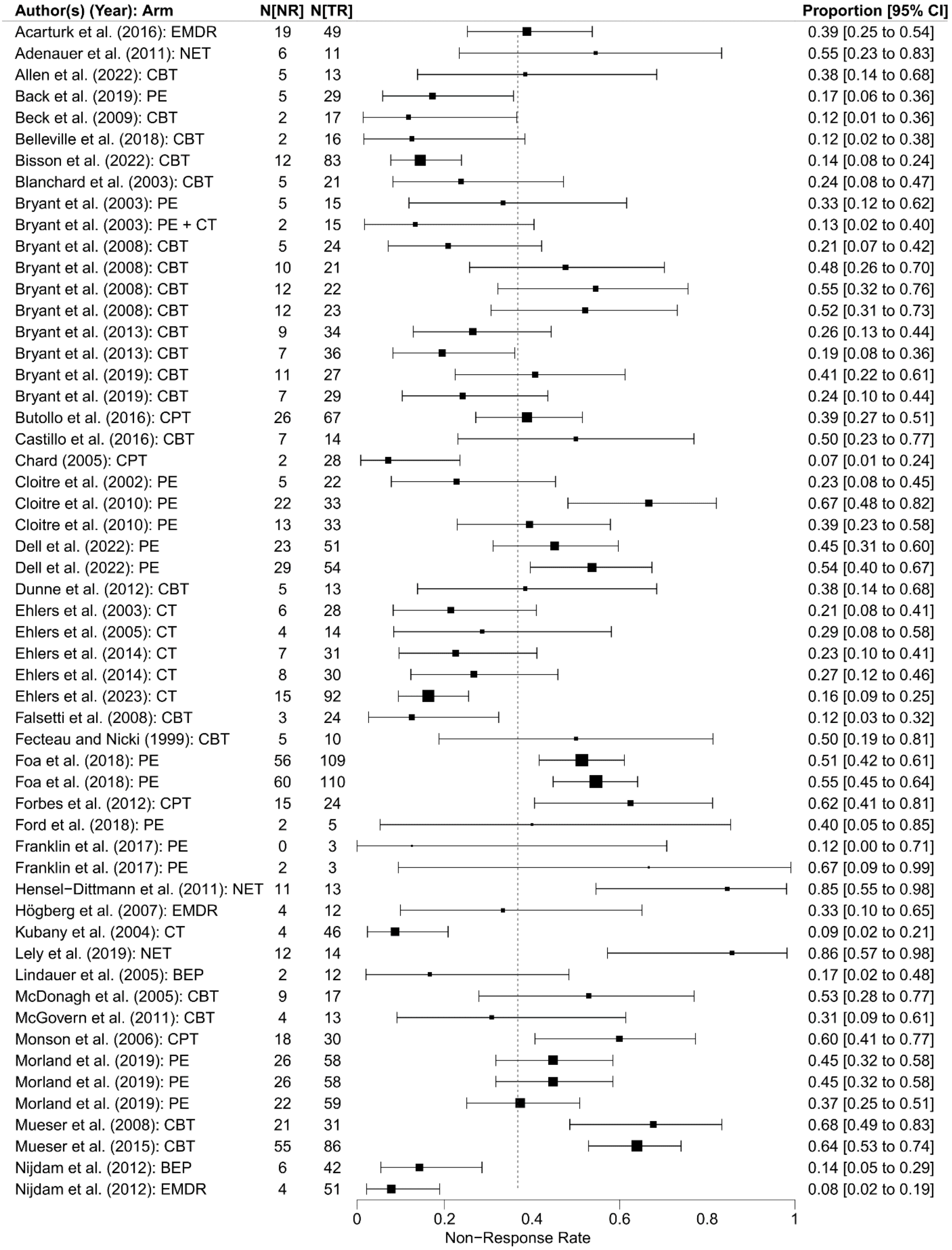
Author(s) (Year): Arm N[NR] N[TR] Proportion [95% CI]
Acarturk et al. (2016): EMDR 19 49 0.39 [0.25 to 0.54]
Adenauer et al. (2011): NET 6 11 0.55 [0.23 to 0.83]
Allen et al. (2022): CBT 5 13 0.38 [0.14 to 0.68]
Back et al. (2019): PE 5 29 0.17 [0.06 to 0.36]
Beck et al. (2009): CBT 2 17 0.12 [0.01 to 0.36]
Belleville et al. (2018): CBT 2 16 0.12 [0.02 to 0.38]
Bisson et al. (2022): CBT 12 83 0.14 [0.08 to 0.24]
Blanchard et al. (2003): CBT 5 21 0.24 [0.08 to 0.47]
Bryant et al. (2003): PE 5 15 0.33 [0.12 to 0.62]
Bryant et al. (2003): PE + CT 2 15 0.13 [0.02 to 0.40]
Bryant et al. (2008): CBT 5 24 0.21 [0.07 to 0.42]
Bryant et al. (2008): CBT 10 21 0.48 [0.26 to 0.70]
Bryant et al. (2008): CBT 12 22 0.55 [0.32 to 0.76]
Bryant et al. (2008): CBT 12 23 0.52 [0.31 to 0.73]
Bryant et al. (2013): CBT 9 34 0.26 [0.13 to 0.44]
Bryant et al. (2013): CBT 7 36 0.19 [0.08 to 0.36]
Bryant et al. (2019): CBT 11 27 0.41 [0.22 to 0.61]
Bryant et al. (2019): CBT 7 29 0.24 [0.10 to 0.44]
Butollo et al. (2016): CPT 26 67 0.39 [0.27 to 0.51]
Castillo et al. (2016): CBT 7 14 0.50 [0.23 to 0.77]
Chard (2005): CPT 2 28 0.07 [0.01 to 0.24]
Cloitre et al. (2002): PE 5 22 0.23 [0.08 to 0.45]
Cloitre et al. (2010): PE 22 33 0.67 [0.48 to 0.82]
Cloitre et al. (2010): PE 13 33 0.39 [0.23 to 0.58]
Dell et al. (2022): PE 23 51 0.45 [0.31 to 0.60]
Dell et al. (2022): PE 29 54 0.54 [0.40 to 0.67]
Dunne et al. (2012): CBT 5 13 0.38 [0.14 to 0.68]
Ehlers et al. (2003): CT 6 28 0.21 [0.08 to 0.41]
Ehlers et al. (2005): CT 4 14 0.29 [0.08 to 0.58]
Ehlers et al. (2014): CT 7 31 0.23 [0.10 to 0.41]
Ehlers et al. (2014): CT 8 30 0.27 [0.12 to 0.46]
Ehlers et al. (2023): CT 15 92 0.16 [0.09 to 0.25]
Falsetti et al. (2008): CBT 3 24 0.12 [0.03 to 0.32]
Fecteau and Nicki (1999): CBT 5 10 0.50 [0.19 to 0.81]
Foa et al. (2018): PE 56 109 0.51 [0.42 to 0.61]
Foa et al. (2018): PE 60 110 0.55 [0.45 to 0.64]
Forbes et al. (2012): CPT 15 24 0.62 [0.41 to 0.81]
Ford et al. (2018): PE 2 5 0.40 [0.05 to 0.85]
Franklin et al. (2017): PE 0 3 0.12 [0.00 to 0.71]
Franklin et al. (2017): PE 2 3 0.67 [0.09 to 0.99]
Hensel−Dittmann et al. (2011): NET 11 13 0.85 [0.55 to 0.98]
Högberg et al. (2007): EMDR 4 12 0.33 [0.10 to 0.65]
Kubany et al. (2004): CT 4 46 0.09 [0.02 to 0.21]
Lely et al. (2019): NET 12 14 0.86 [0.57 to 0.98]
Lindauer et al. (2005): BEP 2 12 0.17 [0.02 to 0.48]
McDonagh et al. (2005): CBT 9 17 0.53 [0.28 to 0.77]
McGovern et al. (2011): CBT 4 13 0.31 [0.09 to 0.61]
Monson et al. (2006): CPT 18 30 0.60 [0.41 to 0.77]
Morland et al. (2019): PE 26 58 0.45 [0.32 to 0.58]
Morland et al. (2019): PE 26 58 0.45 [0.32 to 0.58]
Morland et al. (2019): PE 22 59 0.37 [0.25 to 0.51]
Mueser et al. (2008): CBT 21 31 0.68 [0.49 to 0.83]
Mueser et al. (2015): CBT 55 86 0.64 [0.53 to 0.74]
Nijdam et al. (2012): BEP 6 42 0.14 [0.05 to 0.29]
Nijdam et al. (2012): EMDR 4 51 0.08 [0.02 to 0.19]
0
0.2
0.4
0.6
0.8
1
Non−Response Rate

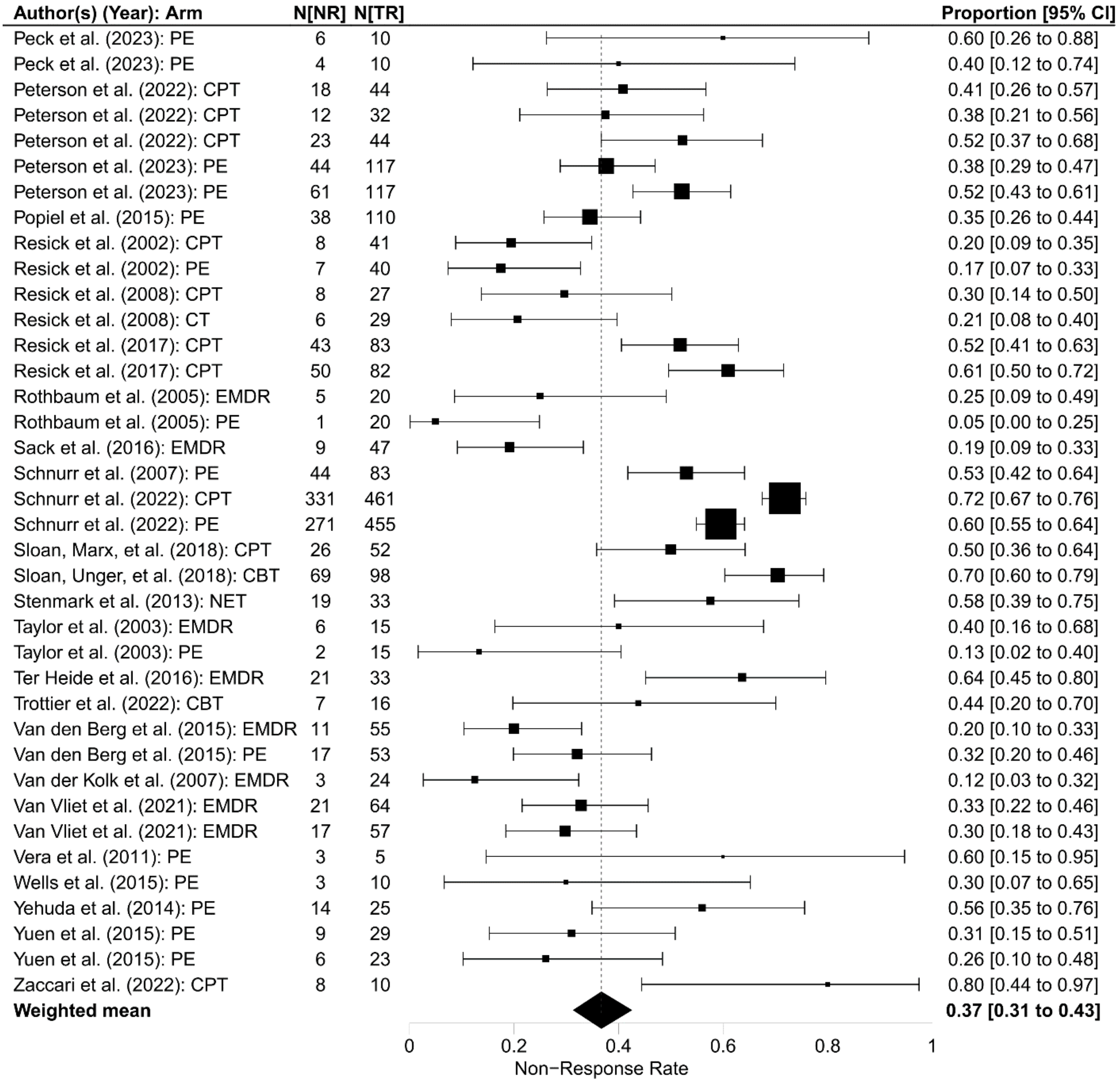

Figure D1 Forest Plot of Non-Response Rate Sub Meta-analysis.

N[NR] = number of non-responders; N[TR] = number in treatment group; CI = confidence interval; CBT = cognitive behavioral therapy; CPT = cognitive processing therapy; CT = cognitive therapy; PE = prolonged exposure therapy; BEP = brief eclectic therapy; EMDR = eye movement desensitization and reprocessing; NET = narrative exposure therapy. Square size indicates study weight. The zero frequency has been trimmed by adding a small constant for computation purposes.

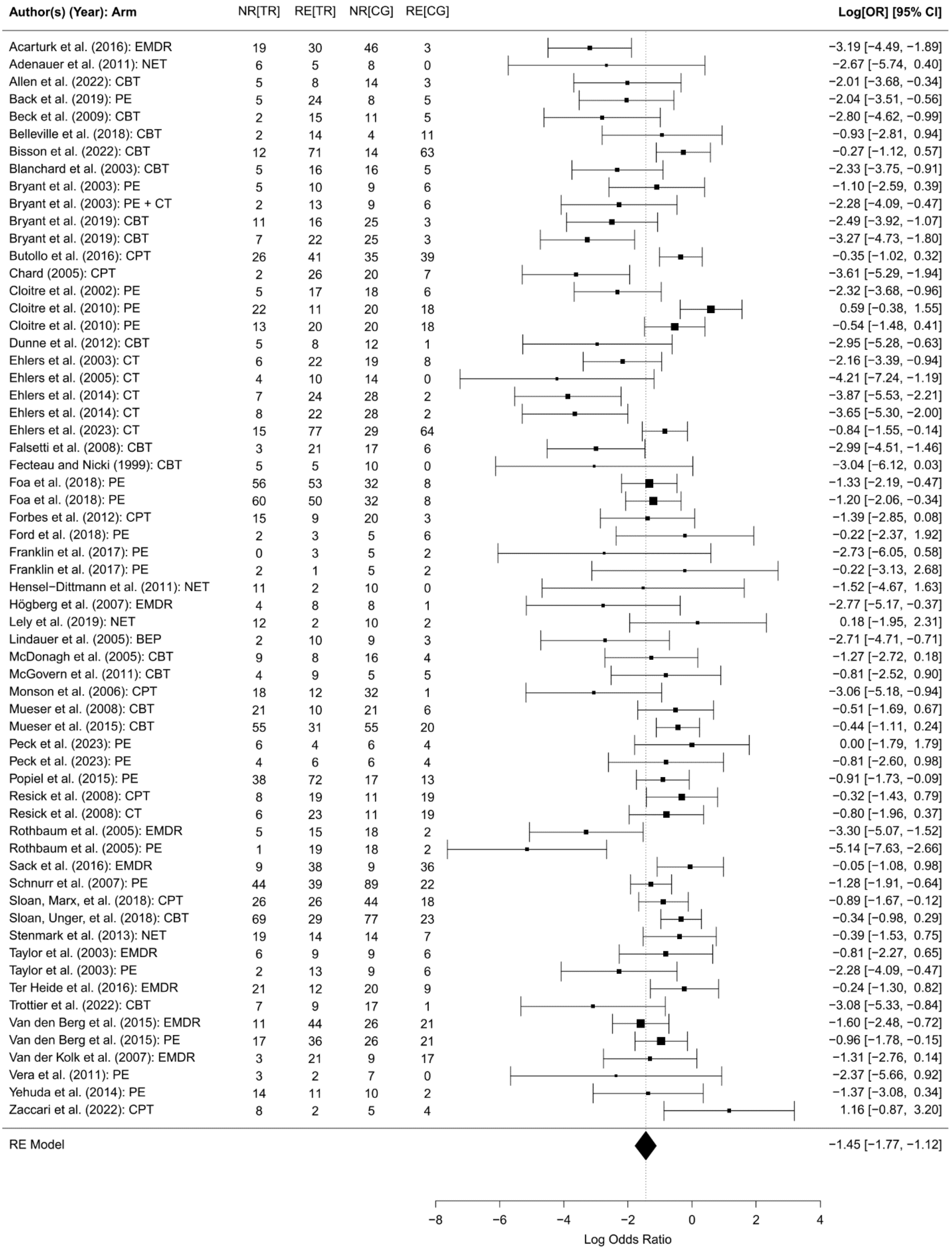

Figure D2 Forest Plot of log OR Sub Meta-analysis

Note. NR[TR] = number of non-responders treatment group; RE[TR] = number of responders treatment group; NR[CG] = number of non-responders control group; RE[CG] = number of responders control group; CI = confidence interval; Log *OR* = log transformed Odds Ratio; CBT = cognitive behavioral therapy; CPT = cognitive processing therapy; CT = cognitive therapy; PE = prolonged exposure therapy; BEP = brief eclectic therapy; EMDR = eye movement desensitization and reprocessing; NET = narrative exposure therapy.

E. References included in the meta-analysis

Acarturk, C., Konuk, E., Cetinkaya, M., Senay, I., Sijbrandij, M., Gulen, B., & Cuijpers, P. (2016). The efficacy of eye movement desensitization and reprocessing for post-traumatic stress disorder and depression among Syrian refugees: results of a randomized controlled trial. *Psychological Medicine*, *46*(12), 2583–2593. https://doi.org/10.1017/S0033291716001070

Adenauer, H., Catani, C., Gola, H., Keil, J., Ruf, M., Schauer, M., & Neuner, F. (2011). Narrative exposure therapy for PTSD increases top-down processing of aversive stimuli - evidence from a randomized controlled treatment trial. *BMC Neuroscience*, *12*(1), 1–13. https://doi.org/10.1186/1471-2202-12-127/FIGURES/7

Allen, A. R., Smith, J., Hobbs, M. J., Loughnan, S. A., Sharrock, M., Newby, J. M., Andrews, G., & Mahoney, A. E. J. (2022). Internet-delivered cognitive behaviour therapy for post-traumatic stress disorder: a randomised controlled trial and outcomes in routine care. *Behavioural and cognitive psychotherapy*, *50*(6), 649-655. https://doi.org/https://dx.doi.org/10.1017/S1352465822000285

Back, S. E., Killeen, T., Badour, C. L., Flanagan, J. C., Allan, N. P., Ana, E. S., Lozano, B., Korte, K. J., Foa, E. B., & Brady, K. T. (2019). Concurrent treatment of substance use disorders and PTSD using prolonged exposure: A randomized clinical trial in military veterans. *Addictive Behaviors*, *90*, 369–377. https://doi.org/10.1016/J.ADDBEH.2018.11.032

Beck, J. G., Coffey, S. F., Foy, D. W., Keane, T. M., & Blanchard, E. B. (2009). Group cognitive behavior therapy for chronic posttraumatic stress disorder: an initial randomized pilot study. *Behavior Therapy*, *40*(1), 82–92. https://doi.org/10.1016/J.BETH.2008.01.003

Belleville, G., Dubé-Frenette, M., & Rousseau, A. (2018). Efficacy of Imagery Rehearsal Therapy and Cognitive Behavioral Therapy in Sexual Assault Victims With Posttraumatic Stress Disorder: A Randomized Controlled Trial. *Journal of Traumatic Stress*, *31*(4), 591–601. https://doi.org/10.1002/JTS.22306

Bisson, J. I., Ariti, C., Cullen, K., Kitchiner, N., Lewis, C., Roberts, N. P., Simon, N., Smallman, K., Addison, K., Bell, V., Brookes-Howell, L., Cosgrove, S., Ehlers, A., Fitzsimmons, D., Foscarini-Craggs, P., Harris, S. R. S., Kelson, M., Lovell, K., McKenna, M., . . . Williams-Thomas, R. (2022). Guided, internet based, cognitive behavioural therapy for post-traumatic stress disorder: pragmatic, multicentre, randomised controlled non-inferiority trial (RAPID). *BMJ (Clinical research ed.)*, *377*, e069405-e069405. https://doi.org/https://dx.doi.org/10.1136/bmj-2021-069405

Blanchard, E. B., Hickling, E. J., Devineni, T., Veazey, C. H., Galovski, T. E., Mundy, E., Malta, L. S., & Buckley, T. C. (2003). A controlled evaluation of cognitive behavioral therapy for posttraumatic stress in motor vehicle accident survivors. *Behaviour Research and Therapy*, *41*(1), 79–96. https://doi.org/10.1016/S0005-7967(01)00131-0

Bohus, M., Dyer, A. S., Priebe, K., Krüger, A., Kleindienst, N., Schmahl, C., Niedtfeld, I., & Steil, R. (2013). Dialectical behaviour therapy for post-traumatic stress disorder after childhood sexual abuse in patients with and without borderline personality disorder: A randomised controlled trial. *Psychotherapy and Psychosomatics*, *82*(4), 221–233. https://doi.org/10.1159/000348451

Brady, F., Chisholm, A., Walsh, E., Ottisova, L., Bevilacqua, L., Mason, C., Von Werthern, M., Cannon, T., Curry, C., Komolafe, K., Robert, R. E., & Robjant, K. (2021). Narrative exposure therapy for survivors of human trafficking: Feasibility randomised controlled trial. *BJPsych Open*, *7*(6), e196-e196. https://doi.org/https://dx.doi.org/10.1192/bjo.2021.1029

Bryant, R. A., Ekasawin, S., Chakrabhand, S., Suwanmitri, S., Duangchun, O., & Chantaluckwong, T. (2011). A randomized controlled effectiveness trial of cognitive behavior therapy for post-traumatic stress disorder in terrorist-affected people in Thailand. *World Psychiatry*, *10*(3), 205. https://doi.org/10.1002/J.2051-5545.2011.TB00058.X

Bryant, R. A., Kenny, L., Rawson, N., Cahill, C., Joscelyne, A., Garber, B., Tockar, J., Dawson, K., & Nickerson, A. (2019). Efficacy of exposure-based cognitive behaviour therapy for post-traumatic stress disorder in emergency service personnel: a randomised clinical trial. *Psychological Medicine*, *49*(9), 1565–1573. https://doi.org/10.1017/S0033291718002234

Bryant, R. A., Mastrodomenico, J., Hopwood, S., Kenny, L., Cahill, C., Kandris, E., & Taylor, K. (2013). Augmenting cognitive behaviour therapy for post-traumatic stress disorder with emotion tolerance training: a randomized controlled trial. *Psychological Medicine*, *43*(10), 2153–2160. https://doi.org/10.1017/S0033291713000068

Bryant, R. A., Moulds, M. L., Guthrie, R. M., Dang, S. T., Mastrodomenico, J., Nixon, R. D. V., Felmingham, K. L., Hopwood, S., & Creamer, M. (2008). A randomized controlled trial of exposure therapy and cognitive restructuring for posttraumatic stress disorder. *Journal of Consulting and Clinical Psychology*, *76*(4), 695. https://doi.org/10.1037/A0012616

Bryant, R. A., Moulds, M. L., Guthrie, R. M., Dang, S. T., & Nixon, R. D. V. (2003). Imaginal exposure alone and imaginal exposure with cognitive restructuring in treatment of posttraumatic stress disorder.

Journal of Consulting and Clinical Psychology, *71*(4), 706–712. https://doi.org/10.1037/0022-006X.71.4.706

Butollo, W., Karl, R., König, J., & Rosner, R. (2016). A Randomized Controlled Clinical Trial of Dialogical Exposure Therapy versus Cognitive Processing Therapy for Adult Outpatients Suffering from PTSD after Type I Trauma in Adulthood. *Psychotherapy and Psychosomatics*, *85*(1), 16–26. https://doi.org/10.1159/000440726

Castillo, D. T., Chee, C. L., Nason, E., Keller, J., C'De Baca, J., Qualls, C., Fallon, S. K., Haaland, K. Y., Miller, M. W., & Keane, T. M. (2016). Group-delivered cognitive/exposure therapy for PTSD in women veterans: A randomized controlled trial. *Psychological Trauma: Theory, Research, Practice, and Policy*, *8*(3), 404–412. https://doi.org/10.1037/TRA0000111

Chard, K. M. (2005). An evaluation of cognitive processing therapy for the treatment of posttraumatic stress disorder related to childhood sexual abuse. *Journal of Consulting and Clinical Psychology*, *73*(5), 965–971. https://doi.org/10.1037/0022-006X.73.5.965

Cloitre, M., Koenen, K. C., Cohen, L. R., & Han, H. (2002). Skills training in affective and interpersonal regulation followed by exposure: A phase-based treatment for PTSD related to childhood abuse. *Journal of Consulting and Clinical Psychology*, *70*(5), 1067–1074. https://doi.org/10.1037//0022-006X.70.5.1067

Cloitre, M., Stovall-McClough, K. C., Nooner, K., Zorbas, P., Cherry, S., Jackson, C. L., Gan, W., & Petkova, E. (2010). Treatment for PTSD related to childhood abuse: A randomized controlled trial. *American Journal of Psychiatry*, *167*(8), 915–924. https://doi.org/10.1176/appi.ajp.2010.09081247

Dell, L., Sbisa, A., Forbes, A., O'Donnell, M., Bryant, R., Hodson, S., Morton, D., Battersby, M., Tuerk, P., Wallace, D., & Forbes, D. (2022). Effect of massed v. standard prolonged exposure therapy on PTSD in military personnel and veterans: a non-inferiority randomised controlled trial. *Psychological Medicine*, 1-8. https://doi.org/https://doi.org/10.1017/S0033291722000927

Dunne, R. L., Kenardy, J., & Sterling, M. (2012). A randomized controlled trial of cognitive-behavioral therapy for the treatment of PTSD in the context of chronic whiplash. *Clinical Journal of Pain*, *28*(9), 755–765. https://doi.org/10.1097/AJP.0B013E318243E16B

Ehlers, A., Clark, D. M., Hackmann, A., McManus, F., Fennell, M., Herbert, C., & Mayou, R. (2003). A Randomized Controlled Trial of Cognitive Therapy, a Self-help Booklet, and Repeated Assessments as Early Interventions for Posttraumatic Stress Disorder. *Archives of General Psychiatry*, *60*(10), 1024–1032. https://doi.org/10.1001/ARCHPSYC.60.10.1024

Ehlers, A., Clark, D. M., Hackmann, A., McManus, F., & Fennell, M. (2005). Cognitive therapy for post-traumatic stress disorder: development and evaluation. *Behav Res Ther*, *43*(4), 413-431. https://doi.org/10.1016/j.brat.2004.03.006

Ehlers, A., Hackmann, A., Grey, N., Wild, J., Liness, S., Albert, I., Deale, A., Stott, R., & Clark, D. M. (2014). A randomized controlled trial of 7-day intensive and standard weekly cognitive therapy for PTSD and emotion-focused supportive therapy. *American Journal of Psychiatry*, *171*(3), 294–304. https://doi.org/10.1176/APPI.AJP.2013.13040552/ASSET/IMAGES/LARGE/294F2.JPEG

Ehlers, A., Wild, J., Warnock-Parkes, E., Grey, N., Murray, H., Kerr, A., Rozental, A., Thew, G., Janecka, M., Beierl, E., Tsiachristas, A., Perera-Salazar, R., Andersson, G., & Clark, D. (2023). Therapist-assisted online psychological therapies differing in trauma focus for post-traumatic stress disorder (STOP-PTSD): a UK-based, single-blind, randomised controlled trial. *Lancet Psychiatry*, *10*(8), 608-622. https://doi.org/https://doi.org/10.1016/S2215-0366(23)00181-5

Falsetti, S. A., Resnick, H. S., & Davis, J. L. (2008). Multiple channel exposure therapy for women with PTSD and comorbid panic attacks. *Cognitive Behaviour Therapy*, *37*(2), 117–130. https://doi.org/10.1080/16506070801969088

Fecteau, G., & Nicki, R. (1999). Cognitive Behavioural Treatment of Post Traumatic Stress Disorder after Motor Vehicle Accident. *Undefined*, *27*(3), 201–214. https://doi.org/10.1017/S135246589927302X

Feske, U. (2008). Treating low-income and minority women with posttraumatic stress disorder: a pilot study comparing prolonged exposure and treatment as usual conducted by community therapists. *Journal of Interpersonal Violence*, *23*(8), 1027–1040. https://doi.org/10.1177/0886260507313967

Foa, E. B., McLean, C. P., Zang, Y., Rosenfield, D., Yadin, E., Yarvis, J. S., Mintz, J., Young-McCaughan, S., Borah, E. V., Dondanville, K. A., Fina, B. A., Hall-Clark, B. N., Lichner, T., Litz, B. T., Roache, J., Wright, E. C., & Peterson, A. L. (2018). Effect of Prolonged Exposure Therapy Delivered Over 2 Weeks vs 8 Weeks vs Present-Centered Therapy on PTSD Symptom Severity in Military Personnel: A Randomized Clinical Trial. *JAMA*, *319*(4), 354–364. https://doi.org/10.1001/JAMA.2017.21242

Forbes, D., Lloyd, D., Nixon, R. D. V., Elliott, P., Varker, T., Perry, D., Bryant, R. A., & Creamer, M. (2012). A multisite randomized controlled effectiveness trial of cognitive processing therapy for military-related posttraumatic stress disorder. *Journal of Anxiety Disorders*, *26*(3), 442–452. https://doi.org/10.1016/J.JANXDIS.2012.01.006

Ford, J. D., Grasso, D. J., Greene, C. A., Slivinsky, M., & DeViva, J. C. (2018). Randomized clinical trial pilot study of prolonged exposure versus present centred affect regulation therapy for PTSD and anger

problems with male military combat veterans. *Clinical Psychology & Psychotherapy*, *25*(5), 641–649. https://doi.org/10.1002/CPP.2194

Franklin, C. L., Cuccurullo, L. A., Walton, J. L., Arseneau, J. R., & Petersen, N. J. (2017). Face to face but not in the same place: A pilot study of prolonged exposure therapy. *Journal of Trauma & Dissociation: The Official Journal of the International Society for the Study of Dissociation (ISSD)*, *18*(1), 116–130. https://doi.org/10.1080/15299732.2016.1205704

Hensel-Dittmann, D., Schauer, M., Ruf, M., Catani, C., Odenwald, M., Elbert, T., & Neuner, F. (2011). Treatment of traumatized victims of war and torture: a randomized controlled comparison of narrative exposure therapy and stress inoculation training. *Psychotherapy and Psychosomatics*, *80*(6), 345–352. https://doi.org/10.1159/000327253

Hinton, D. E., Hofmann, S. G., Rivera, E., Otto, M. W., & Pollack, M. H. (2011). Culturally adapted CBT (CA-CBT) for Latino women with treatment-resistant PTSD: a pilot study comparing CA-CBT to applied muscle relaxation. *Behaviour Research and Therapy*, *49*(4), 275–280. https://doi.org/10.1016/J.BRAT.2011.01.005

Högberg, G., Pagani, M., Sundin, Ö., Soares, J., Åberg-Wistedt, A., Tärnell, B., & Hällström, T. (2007). On treatment with eye movement desensitization and reprocessing of chronic post-traumatic stress disorder in public transportation workers--a randomized controlled trial. *Nordic Journal of Psychiatry*, *61*(1), 54–61. https://doi.org/10.1080/08039480601129408

Hollifield, M., Sinclair-Lian, N., Warner, T. D., & Hammerschlag, R. (2007). Acupuncture for posttraumatic stress disorder: a randomized controlled pilot trial. *The Journal of Nervous and Mental Disease*, *195*(6), 504–513. https://doi.org/10.1097/NMD.0B013E31803044F8

Karatzias, T., Power, K., Brown, K., McGoldrick, T., Begum, M., Young, J., Loughran, P., Chouliara, Z., & Adams, S. (2011). A controlled comparison of the effectiveness and efficiency of two psychological therapies for posttraumatic stress disorder: eye movement desensitization and reprocessing vs. emotional freedom techniques. *The Journal of Nervous and Mental Disease*, *199*(6), 372–378. https://doi.org/10.1097/NMD.0B013E31821CD262

Kubany, E. S., Hill, E. E., Owens, J. A., Iannce-Spencer, C., McCaig, M. A., Tremayne, K. J., & Williams, P. L. (2004). Cognitive trauma therapy for battered women with PTSD (CTT-BW). *Journal of Consulting and Clinical Psychology*, *72*(1), 3–18. https://doi.org/10.1037/0022-006X.72.1.3

Langkaas, T. F., Hoffart, A., Øktedalen, T., Ulvenes, P. G., Hembree, E. A., & Smucker, M. (2017). Exposure and non-fear emotions: A randomized controlled study of exposure-based and rescripting-based imagery in PTSD treatment. *Behaviour Research and Therapy*, *97*, 33–42. https://doi.org/10.1016/J.BRAT.2017.06.007

Lely, J. C. G., Knipscheer, J. W., Moerbeek, M., Ter Heide, F. J. J., Van Den Bout, J., & Kleber, R. J. (2019). Randomised controlled trial comparing narrative exposure therapy with present-centred therapy for older patients with post-traumatic stress disorder. *The British Journal of Psychiatry : The Journal of Mental Science*, *214*(6), 369–377. https://doi.org/10.1192/BJP.2019.59

Lindauer, R. J. L., Gersons, B. P. R., Van Meijel, E. P. M., Blom, K., Carlier, I. V. E., Vrijlandt, I., & Olff, M. (2005). Effects of brief eclectic psychotherapy in patients with posttraumatic stress disorder: randomized clinical trial. *Journal of Traumatic Stress*, *18*(3), 205–212. https://doi.org/10.1002/JTS.20029

Markowitz, J. C., Petkova, E., Neria, Y., Van Meter, P. E., Zhao, Y., Hembree, E., Lovell, K., Biyanova, T., & Marshall, R. D. (2015). Is Exposure Necessary? A Randomized Clinical Trial of Interpersonal Psychotherapy for PTSD. *The American Journal of Psychiatry*, *172*(5), 430–440. https://doi.org/10.1176/APPI.AJP.2014.14070908

Maxwell, K., Callahan, J. L., Holtz, P., Janis, B. M., Gerber, M. M., & Connor, D. R. (2016). Comparative study of group treatments for posttraumatic stress disorder. *Psychotherapy (Chicago, Ill.)*, *53*(4), 433–445. https://doi.org/10.1037/PST0000032

McDonagh, A., McHugo, G., Sengupta, A., Demment, C. C., Schnurr, P. P., Friedman, M., Ford, J., Mueser, K., Founder, D., & Descamps, M. (2005). Randomized trial of cognitive-behavioral therapy for chronic posttraumatic stress disorder in adult female survivors of childhood sexual abuse. *Journal of Consulting and Clinical Psychology*, *73*(3), 515–524. https://doi.org/10.1037/0022-006X.73.3.515

McGovern, M. P., Lambert-Harris, C., Alterman, A. I., Xie, H., & Meier, A. (2011). A Randomized Controlled Trial Comparing Integrated Cognitive Behavioral Therapy Versus Individual Addiction Counseling for Co-occurring Substance Use and Posttraumatic Stress Disorders. *Journal of Dual Diagnosis*, *7*(4), 207–227. https://doi.org/10.1080/15504263.2011.620425

McLay, R. N., Baird, A., Webb-Murphy, J., Deal, W., Tran, L., Anson, H., Klam, W., & Johnston, S. (2017). A Randomized, Head-to-Head Study of Virtual Reality Exposure Therapy for Posttraumatic Stress Disorder. *Cyberpsychology, Behavior and Social Networking*, *20*(4), 218–224. https://doi.org/10.1089/CYBER.2016.0554

Monson, C. M., Schnurr, P. P., Resick, P. A., Friedman, M. J., Young-Xu, Y., & Stevens, S. P. (2006). Cognitive processing therapy for veterans with military-related posttraumatic stress disorder. *Journal of Consulting and Clinical Psychology*, *74*(5), 898–907. https://doi.org/10.1037/0022-006X.74.5.898

Morland, L. A., Mackintosh, M. A., Glassman, L. H., Wells, S. Y., Thorp, S. R., Rauch, S. A. M., Cunningham, P. B., Tuerk, P. W., Grubbs, K. M., Golshan, S., Sohn, M. J., & Acierno, R. (2019). Home-based delivery of variable length prolonged exposure therapy: A comparison of clinical efficacy between service modalities. *Depression and Anxiety*, *37*(4), 346–355. https://doi.org/10.1002/DA.22979

Mueser, K. T., Gottlieb, J. D., Xie, H., Lu, W., Yanos, P. T., Rosenberg, S. D., Silverstein, S. M., Duva, S. M., Minsky, S., Wolfe, R. S., & McHugo, G. J. (2015). Evaluation of cognitive restructuring for post-traumatic stress disorder in people with severe mental illness. *The British Journal of Psychiatry : The Journal of Mental Science*, *206*(6), 501–508. https://doi.org/10.1192/BJP.BP.114.147926

Mueser, K. T., Rosenberg, S. D., Xie, H., Jankowski, M. K., Bolton, E. E., Lu, W., Hamblen, J. L., Rosenberg, H. J., McHugo, G. J., & Wolfe, R. (2008). A randomized controlled trial of cognitive-behavioral treatment for posttraumatic stress disorder in severe mental illness. *Journal of Consulting and Clinical Psychology*, *76*(2), 259–271. https://doi.org/10.1037/0022-006X.76.2.259

Nidich, S., Mills, P. J., Rainforth, M., Heppner, P., Schneider, R. H., Rosenthal, N. E., Salerno, J., Gaylord-King, C., & Rutledge, T. (2018). Non-trauma-focused meditation versus exposure therapy in veterans with post-traumatic stress disorder: a randomised controlled trial. *The Lancet. Psychiatry*, *5*(12), 975–986. https://doi.org/10.1016/S2215-0366(18)30384-5

Nijdam, M. J., Gersons, B. P. R., Reitsma, J. B., De Jongh, A., & Olff, M. (2012). Brief eclectic psychotherapy v. eye movement desensitisation and reprocessing therapy for post-traumatic stress disorder: randomised controlled trial. *The British Journal of Psychiatry : The Journal of Mental Science*, *200*(3), 224–231. https://doi.org/10.1192/BJP.BP.111.099234

Peck, K. R., Badger, G. J., Cole, R., Higgins, S. T., Moxley-Kelly, N., & Sigmon, S. C. (2023). Prolonged exposure therapy for PTSD in individuals with opioid use disorder: A randomized pilot study. *Addictive Behaviors*, *143*, 1-8. https://doi.org/10.1016/j.addbeh.2023.107688

Peterson, A. L., Blount, T. H., Foa, E. B., Brown, L. A., McLean, C. P., Mintz, J., Schobitz, R. P., DeBeer, B. R., Mignogna, J., Fina, B. A., Evans, W. R., Synett, S., Hall-Clark, B. N., Rentz, T. O., Schrader, C., Yarvis, J. S., Dondanville, K. A., Hansen, H., Jacoby, V. M., . . . Keane, T. M. (2023). Massed vs Intensive Outpatient Prolonged Exposure for Combat-Related Posttraumatic Stress Disorder: A Randomized Clinical Trial. *JAMA network open*, *6*(1), e2249422-e2249422. https://doi.org/https://dx.doi.org/10.1001/jamanetworkopen.2022.49422

Peterson, A. L., Mintz, J., Moring, J. C., Straud, C. L., Young-McCaughan, S., McGeary, C. A., McGeary, D. D., Litz, B. T., Velligan, D. I., Macdonald, A., Mata-Galan, E., Holliday, S. L., Dillon, K. H., Roache, J. D., Bira, L. M., Nabity, P. S., Medellin, E. M., Hale, W. J., & Resick, P. A. (2022). In-office, in-home, and telehealth cognitive processing therapy for posttraumatic stress disorder in veterans: a randomized clinical trial. *BMC Psychiatry*, *22*(1), 41-41. https://doi.org/https://dx.doi.org/10.1186/s12888-022-03699-4

Popiel, A., Zawadzki, B., Pragłowska, E., & Teichman, Y. (2015). Prolonged exposure, paroxetine and the combination in the treatment of PTSD following a motor vehicle accident. A randomized clinical trial - The "tRAKT" study. *Journal of Behavior Therapy and Experimental Psychiatry*, *48*, 17–26. https://doi.org/10.1016/J.JBTEP.2015.01.002

Rauch, S. A. M., King, A. P., Abelson, J., Tuerk, P. W., Smith, E., Rothbaum, B. O., Clifton, E., Defever, A., & Liberzon, I. (2015). Biological and symptom changes in posttraumatic stress disorder treatment: A randomized clinical trial. *Depression and Anxiety*, *32*(3), 204–212. https://doi.org/10.1002/DA.22331

Ready, D. J., Mascaro, N., Wattenberg, M. S., Sylvers, P., Worley, V., & Bradley-Davino, B. (2018). A Controlled Study of Group-Based Exposure Therapy with Vietnam-Era Veterans. *Https://Doi.Org/10.1080/15325024.2018.1485268*, *23*(6), 439–457. https://doi.org/10.1080/15325024.2018.1485268

Reger, G. M., Koenen-Woods, P., Zetocha, K., Smolenski, D. J., Holloway, K. M., Rothbaum, B. O., Difede, J. A., Rizzo, A. A., Edwards-Stewart, A., Skopp, N. A., Mishkind, M., Reger, M. A., & Gahm, G. A. (2016). Randomized controlled trial of prolonged exposure using imaginal exposure vs. virtual reality exposure in active duty soldiers with deployment-related posttraumatic stress disorder (PTSD). *Journal of Consulting and Clinical Psychology*, *84*(11), 946–959. https://doi.org/10.1037/CCP0000134

Resick, P. A., Galovski, T. E., Uhlmansiek, M. O. B., Scher, C. D., Clum, G. A., & Young-Xu, Y. (2008). A randomized clinical trial to dismantle components of cognitive processing therapy for posttraumatic stress disorder in female victims of interpersonal violence. *Journal of Consulting and Clinical Psychology*, *76*(2), 243–258. https://doi.org/10.1037/0022-006X.76.2.243

Resick, P. A., Nishith, P., Weaver, T. L., Astin, M. C., & Feuer, C. A. (2002). A comparison of cognitive-processing therapy with prolonged exposure and a waiting condition for the treatment of chronic posttraumatic stress disorder in female rape victims. *Journal of Consulting and Clinical Psychology*, *70*(4), 867–879. https://doi.org/10.1037//0022-006X.70.4.867

Resick, P. A., Wachen, J. S., Dondanville, K. A., Pruiksma, K. E., Yarvis, J. S., Peterson, A. L., Mintz, J., Borah, E. V., Brundige, A., Hembree, E. A., Litz, B. T., Roache, J. D., & Young-McCaughan, S. (2017). Effect of Group vs Individual Cognitive Processing Therapy in Active-Duty Military Seeking Treatment for Posttraumatic Stress Disorder: A Randomized Clinical Trial. *JAMA Psychiatry*, *74*(1), 28–36. https://doi.org/10.1001/JAMAPSYCHIATRY.2016.2729

Resick, P. A., Wachen, J. S., Mintz, J., Young-McCaughan, S., Roache, J. D., Borah, A. M., Borah, E. V., Dondanville, K. A., Hembree, E. A., Litz, B. T., & Peterson, A. L. (2015). A randomized clinical trial of group cognitive processing therapy compared with group present-centered therapy for PTSD among active duty military personnel. *Journal of Consulting and Clinical Psychology*, *83*(6), 1058–1068. https://doi.org/10.1037/CCP0000016

Rothbaum, B. O., Astin, M. C., & Marsteller, F. (2005). Prolonged exposure versus Eye Movement Desensitization and Reprocessing (EMDR) for PTSD rape victims. *Journal of Traumatic Stress*, *18*(6), 607–616. https://doi.org/10.1002/jts.20069

Sack, M., Zehl, S., Otti, A., Lahmann, C., Henningsen, P., Kruse, J., & Stingl, M. (2016). A Comparison of Dual Attention, Eye Movements, and Exposure Only during Eye Movement Desensitization and Reprocessing for Posttraumatic Stress Disorder: Results from a Randomized Clinical Trial. *Psychotherapy and Psychosomatics*, *85*(6), 357–365. https://doi.org/10.1159/000447671

Schacht, R. L., Brooner, R. K., King, V. L., Kidorf, M. S., & Peirce, J. M. (2017). Incentivizing attendance to prolonged exposure for PTSD with opioid use disorder patients: A randomized controlled trial. *Journal of Consulting and Clinical Psychology*, *85*(7), 689–701. https://doi.org/10.1037/CCP0000208

Schnurr, P. P., Chard, K. M., Ruzek, J. I., Chow, B. K., Resick, P. A., Foa, E. B., Marx, B. P., Friedman, M. J., Bovin, M. J., Caudle, K. L., Castillo, D., Curry, K. T., Hollifield, M., Huang, G. D., Chee, C. L., Astin, M. C., Dickstein, B., Renner, K., Clancy, C. P., . . . Shih, M.-C. (2022). Comparison of Prolonged Exposure vs Cognitive Processing Therapy for Treatment of Posttraumatic Stress Disorder Among US Veterans: A Randomized Clinical Trial. *JAMA network open*, *5*(1), e2136921-e2136921. https://doi.org/https://dx.doi.org/10.1001/jamanetworkopen.2021.36921

Schnurr, P. P., Friedman, M. J., Engel, C. C., Foa, E. B., Shea, M. T., Chow, B. K., Resick, P. A., Thurston, V., Orsillo, S. M., Haug, R., Turner, C., & Bernardy, N. (2007). Cognitive behavioral therapy for posttraumatic stress disorder in women: a randomized controlled trial. *JAMA*, *297*(8), 820–830. https://doi.org/10.1001/JAMA.297.8.820

Schnurr, P. P., Friedman, M. J., Foy, D. W., Shea, M. T., Hsieh, F. Y., Lavori, P. W., Glynn, S. M., Wattenberg, M., & Bernardy, N. C. (2003). Randomized trial of trauma-focused group therapy for posttraumatic stress disorder: results from a department of veterans affairs cooperative study. *Archives of General Psychiatry*, *60*(5), 481–489. https://doi.org/10.1001/ARCHPSYC.60.5.481

Sloan, D. M., Marx, B. P., Lee, D. J., & Resick, P. A. (2018). A Brief Exposure-Based Treatment vs Cognitive Processing Therapy for Posttraumatic Stress Disorder: A Randomized Noninferiority Clinical Trial. *JAMA Psychiatry*, *75*(3), 233–239. https://doi.org/10.1001/JAMAPSYCHIATRY.2017.4249

Sloan, D. M., Unger, W., Lee, D. J., & Beck, J. G. (2018). A Randomized Controlled Trial of Group Cognitive Behavioral Treatment for Veterans Diagnosed With Chronic Posttraumatic Stress Disorder. *Journal of Traumatic Stress*, *31*(6), 886–898. https://doi.org/10.1002/JTS.22338

Stenmark, H., Catani, C., Neuner, F., Elbert, T., & Holen, A. (2013). Treating PTSD in refugees and asylum seekers within the general health care system. A randomized controlled multicenter study. *Behaviour Research and Therapy*, *51*(10), 641–647. https://doi.org/10.1016/J.BRAT.2013.07.002

Taylor, D. J., Pruiksma, K. E., Mintz, J., Slavish, D. C., Wardle-Pinkston, S., Dietch, J. R., Dondanville, K. A., Young-McCaughan, S., Nicholson, K. L., Litz, B. T., Keane, T. M., Peterson, A. L., & Resick, P. A. (2023). Treatment of comorbid sleep disorders and posttraumatic stress disorder in US active duty military personnel: A pilot randomized clinical trial. *Journal of Traumatic Stress, 36*(4), 712-726. https://doi.org/10.1002/jts.22939

Taylor, S., Thordarson, D. S., Fedoroff, I. C., Maxfield, L., Lovell, K., & Ogrodniczuk, J. (2003). Comparative efficacy, speed, and adverse effects of three PTSD treatments: exposure therapy, EMDR, and relaxation training. *Journal of Consulting and Clinical Psychology*, *71*(2), 330–338. https://doi.org/10.1037/0022-006X.71.2.330

Ter Heide, F. J. J., Mooren, T. M., Van De Schoot, R., De Jongh, A., & Kleber, R. J. (2016). Eye movement desensitisation and reprocessing therapy v. stabilisation as usual for refugees: randomised controlled trial. *The British Journal of Psychiatry : The Journal of Mental Science*, *209*(4), 311–318. https://doi.org/10.1192/BJP.BP.115.167775

Thompson-Hollands, J., Lunney, C. A., Sloan, D. M., Wiltsey Stirman, S., & Schnurr, P. P. (2023). Treatment length and symptom improvement in prolonged exposure and present-centered therapy for posttraumatic stress disorder: Comparing dose–response and good-enough level models in two manualized interventions. *Journal of Consulting and Clinical Psychology*, *91*(10), 596-605. https://doi.org/10.1037/ccp0000834

Trottier, K., Monson, C. M., Wonderlich, S. A., & Crosby, R. D. (2022). Results of the first randomized controlled trial of integrated cognitive-behavioral therapy for eating disorders and posttraumatic stress disorder. *Psychological Medicine*, *52*(3), 587-596. https://doi.org/https://dx.doi.org/10.1017/S0033291721004967

Van Den Berg, D. P. G., De Bont, P. A. J. M., Van Der Vleugel, B. M., De Roos, C., De Jongh, A., Van Minnen, A., & Van Der Gaag, M. (2015). Prolonged exposure vs eye movement desensitization and reprocessing vs waiting list for posttraumatic stress disorder in patients with a psychotic disorder: a randomized clinical trial. *JAMA Psychiatry*, *72*(3), 259–267. https://doi.org/10.1001/JAMAPSYCHIATRY.2014.2637

Van Der Kolk, B. A., Spinazzola, J., Blaustein, M. E., Hopper, J. W., Hopper, E. K., Korn, D. L., & Simpson, W. B. (2007). A randomized clinical trial of eye movement desensitization and reprocessing (EMDR), fluoxetine, and pill placebo in the treatment of posttraumatic stress disorder: treatment effects and long-term maintenance. *The Journal of Clinical Psychiatry*, *68*(1), 37–46. https://doi.org/10.4088/JCP.V68N0105

van Vliet, N. I., Huntjens, R. J. C., van Dijk, M. K., Bachrach, N., Meewisse, M.-L., & de Jongh, A. (2021). Phase-based treatment versus immediate trauma-focused treatment for post-traumatic stress disorder due to childhood abuse: Randomised clinical trial. *BJPsych Open*, *7*(6), 20211057-20211057. https://doi.org/https://dx.doi.org/10.1192/bjo.2021.1057

Vera, M., Reyes-Rabanillo, M. L., Juarbe, D., Pérez-Pedrogo, C., Olmo, A., Kichic, R., & Chaplin, W. F. (2011). Prolonged exposure for the treatment of Spanish-speaking Puerto Ricans with posttraumatic stress disorder: A feasibility study. *BMC Research Notes*, *4*(1), 1–8. https://doi.org/10.1186/1756-0500-4-415/FIGURES/1

Wells, A., Walton, D., Lovell, K., & Proctor, D. (2015). Metacognitive Therapy Versus Prolonged Exposure in Adults with Chronic Post-traumatic Stress Disorder: A Parallel Randomized Controlled Trial. *Cognitive Therapy and Research*, *39*(1), 70–80. https://doi.org/10.1007/S10608-014-9636-6

Yehuda, R., Pratchett, L. C., Elmes, M. W., Lehrner, A., Daskalakis, N. P., Koch, E., Makotkine, I., Flory, J. D., & Bierer, L. M. (2014). Glucocorticoid-related predictors and correlates of post-traumatic stress disorder treatment response in combat veterans. *Interface Focus*, *4*(5). https://doi.org/10.1098/RSFS.2014.0048

Yuen, E. K., Gros, D. F., Price, M., Zeigler, S., Tuerk, P. W., Foa, E. B., & Acierno, R. (2015). Randomized Controlled Trial of Home-Based Telehealth Versus In-Person Prolonged Exposure for Combat-Related PTSD in Veterans: Preliminary Results. *Journal of Clinical Psychology*, *71*(6), 500–512. https://doi.org/10.1002/JCLP.22168

Zaccari, B., Sherman, A. D. F., Febres-Cordero, S., Higgins, M., & Kelly, U. (2022). Findings from a pilot study of Trauma Center Trauma-Sensitive Yoga versus cognitive processing therapy for PTSD related to military sexual trauma among women Veterans. *Complementary therapies in medicine*, *70*, 102850-102850. https://doi.org/https://dx.doi.org/10.1016/j.ctim.2022.102850

F. List of excluded studies

Citation	Reason for exclusion
Acierno, R., Jaffe, A. E., Gilmore, A. K., Birks, A., Denier, C., Muzzy, W., Lopez, C. M., Tuerk, P., & Grubaugh, A. L. (2021). A randomized clinical trial of in-person vs. home-based telemedicine delivery of Prolonged Exposure for PTSD in military sexual trauma survivors. *Journal of anxiety disorders*, *83*, 102461. https://doi.org/https://dx.doi.org/10.1016/j.janxdis.2021.102461	No dichotomous clinician-rated or self-reported response outcome related to change in PTSD symptoms
Acierno, R., Knapp, R., Tuerk, P., Gilmore, A. K., Lejuez, C., Ruggiero, K., Muzzy, W., Egede, L., Hernandez-Tejada, M. A., & Foa, E. B. (2017). A non-inferiority trial of Prolonged Exposure for posttraumatic stress disorder: In person versus home-based telehealth. *Behaviour research and therapy*, *89*, 57-65. https://doi.org/10.1016/j.brat.2016.11.009	No dichotomous clinician-rated or self-reported response outcome related to change in PTSD symptoms
Akbarian, F., Bajoghli, H., Haghighi, M., Kalak, N., Holsboer-Trachsler, E., & Brand, S. (2015). The effectiveness of cognitive behavioral therapy with respect to psychological symptoms and recovering autobiographical memory in patients suffering from post-traumatic stress disorder. *Neuropsychiatric disease and treatment*, *11*, 395-404. https://doi.org/10.2147/NDT.S79581	No clinician-based PTSD-diagnosis based on a structured interview at inclusion
Alghamdi, M., Hunt, N., & Thomas, S. (2015). The effectiveness of narrative exposure therapy with traumatised firefighters in Saudi Arabia: A randomized controlled study. *Behaviour research and therapy*, *66*, 64-71. https://doi.org/10.1016/j.brat.2015.01.008	No full PTSD-diagnosis required for inclusion
Alpert, E., Hayes, A. M., Barnes, J. B., & Sloan, D. M. (2023). Using Client Narratives to Identify Predictors of Outcome in Written Exposure Therapy and Cognitive Processing Therapy. *Behavior therapy*, *54*(2), 185-199. https://doi.org/https://dx.doi.org/10.1016/j.beth.2022.09.002	Secondary analyses
Andersson, G., Olsson, E., Ringsgard, E., Sandgren, T., Viklund, I., Andersson, C., Hesselman, Y., Johansson, R., Nordgren, L. B., & Bohman, B. (2021). Individually tailored Internet-delivered cognitive-behavioral therapy for survivors of intimate partner violence: A randomized controlled pilot trial. *Internet interventions*, *26*, 100453. https://doi.org/https://dx.doi.org/10.1016/j.invent.2021.100453	No full PTSD-diagnosis required for inclusion
Arditte Hall, K. A., Werner, K. B., Griffin, M. G., & Galovski, T. E. (2021). The effects of cognitive processing therapy + hypnosis on objective sleep quality in women with posttraumatic stress disorder. *Psychological Trauma: Theory, Research, Practice, and Policy*, *13*(6), 652-656. https://doi.org/10.1037/tra0000970	Secondary analyses
Arntz, A., Tiesema, M., & Kindt, M. (2007). Treatment of PTSD: A comparison of imaginal exposure with and without imagery rescripting. *Journal of behavior therapy and experimental psychiatry*, *38*(4), 345-370. https://doi.org/https://doi.org/10.1016/j.jbtep.2007.10.006	Did not apply DSM-IV, DSM-5, or ICD-10 criteria for PTSD
Asukai, N., Saito, A., Tsuruta, N., Kishimoto, J., & Nishikawa, T. (2010). Efficacy of exposure therapy for Japanese patients with posttraumatic stress disorder due to mixed traumatic events: A randomized controlled study. *J Trauma Stress*, *23*(6), 744-750. https://doi.org/10.1002/jts.20589	Data do not allow conclusions on the number of non-responders per experimental and control group
Bayley, P., Schulz-Heik, J., Tang, J., Mathersul, D., Avery, T., Wong, M., Zeitzer, J., Rosen, C., Burn, A., Hernandez, B., Lazzeroni, L., & Seppälä, E. (2022). Randomised clinical non-inferiority trial of breathing-based meditation and cognitive processing therapy for symptoms of post-traumatic stress disorder in military veterans. *BMJ Open*, *12*(8). https://doi.org/https://doi.org/10.1136/bmjopen-2021-056609	No full PTSD-diagnosis required for inclusion
Beidel, D. C., Frueh, B. C., Uhde, T. W., Wong, N., & Mentrikoski, J. M. (2011). Multicomponent behavioral treatment for chronic combat-related posttraumatic stress disorder: a randomized controlled trial. *J Anxiety Disord*, *25*(2), 224-231. https://doi.org/10.1016/j.janxdis.2010.09.006	No dichotomous clinician-rated or self-reported response outcome related to change in PTSD symptoms
Bellehsen, M., Stoycheva, V., Cohen, B. H., & Nidich, S. (2022). A Pilot Randomized Controlled Trial of Transcendental Meditation as Treatment for Posttraumatic Stress Disorder in Veterans. *Journal of traumatic stress*, *35*(1), 22-31. https://doi.org/https://dx.doi.org/10.1002/jts.22665	Not a psychotherapeutic guideline-recommended intervention
Benfer, N., Darnell, B. C., Rusowicz-Orazem, L., Fielstein, E. M., Grunthal, B., Lehavot, K., Marx, B. P., & Litz, B. (2023). An examination of the criterion-related validity of varying methods of indexing clinically significant change in posttraumatic stress disorder treatment. *Psychological Trauma: Theory, Research, Practice, and Policy*. https://doi.org/10.1037/tra0001479	Secondary analyses
Bichescu, D., Neuner, F., Schauer, M., & Elbert, T. (2007). Narrative exposure therapy for political imprisonment-related chronic posttraumatic stress disorder and depression. *Behav Res Ther*, *45*(9), 2212-2220. https://doi.org/10.1016/j.brat.2006.12.006	Post-assessment more than six weeks after the end of treatment

Citation	Reason for exclusion
Bottche, M., Wagner, B., Vohringer, M., Heinrich, M., Stein, J., Selmo, P., Stammel, N., & Knaevelsrud, C. (2021). Is only one cognitive technique also effective? Results from a randomized controlled trial of two different versions of an internet-based cognitive behavioural intervention for post-traumatic stress disorder in Arabic-speaking countries. *European Journal of Psychotraumatology*, *12*(1), 1943870. https://doi.org/https://dx.doi.org/10.1080/20008198.2021.1943870	Not a psychotherapeutic guideline-recommended intervention
Bragesjo, M., Arnberg, F. K., Olofsdotter Lauri, K., Aspvall, K., Sarnholm, J., & Andersson, E. (2023). Condensed Internet-delivered prolonged exposure provided soon after trauma: a randomised trial. *Psychological Medicine*, *53*(5), 1989-1998. https://doi.org/https://dx.doi.org/10.1017/S0033291721003706	No full PTSD-diagnosis required for inclusion
Bragesjo, M., Arnberg, F. K., Sarnholm, J., Olofsdotter Lauri, K., & Andersson, E. (2021). Condensed internet-delivered prolonged exposure provided soon after trauma: A randomised pilot trial. *Internet interventions*, *23*, 100358. https://doi.org/https://dx.doi.org/10.1016/j.invent.2020.100358	No full PTSD-diagnosis required for inclusion
Brom, D., Kleber, R. J., & Defares, P. B. (1989). Brief psychotherapy for posttraumatic stress disorders. *Journal of Consulting and Clinical Psychology*, *57*(5), 607. https://doi.org/10.1037//0022-006x.57.5.607	Did not apply DSM-IV, DSM-5, or ICD-10 criteria for PTSD
Brown, D. G., Flanagan, J. C., Jarnecke, A., Killeen, T. K., & Back, S. E. (2022). Ethnoracial differences in treatment-seeking veterans with substance use disorders and co-occurring PTSD: Presenting characteristics and response to integrated exposure-based treatment. *Journal of ethnicity in substance abuse*, *21*(3), 1141-1164. https://doi.org/https://dx.doi.org/10.1080/15332640.2020.1836699	Secondary analyses
Bryant, R., Dawson, K., Azevedo, S., Yadav, S., Cahill, C., Kenny, L., Maccallum, F., Tran, J., Rawson, N., Tockar, J., Garber, B., & Keyan, D. (2022). Augmenting trauma-focused psychotherapy for post-traumatic stress disorder with brief aerobic exercise in Australia: a randomised clinical trial. *Lancet Psychiatry*, *10*(1), 21-29. https://doi.org/https://doi.org/10.1016/S2215-0366(22)00368-6	No dichotomous clinician-rated or self-reported response outcome related to change in PTSD symptoms
Bryant, R., Kenny, L., Rawson, N., Cahill, C., Joscelyne, A., Garber, B., Tockar, J., Tran, J., & Dawson, K. (2021). Two-year follow-up of trauma-focused cognitive behavior therapy for posttraumatic stress disorder in emergency service personnel: a randomized clinical trial. *Depression and Anxiety*, *38*(11), 1131-1137. https://doi.org/https://doi.org/10.1002/da.23214	No dichotomous clinician-rated or self-reported response outcome related to change in PTSD symptoms
Buhmann, C. B., Nordentoft, M., Ekstroem, M., Carlsson, J., & Mortensen, E. L. (2016). The effect of flexible cognitive-behavioural therapy and medical treatment, including antidepressants on post-traumatic stress disorder and depression in traumatised refugees: pragmatic randomised controlled clinical trial. *Br J Psychiatry*, *208*(3), 252-259. https://doi.org/10.1192/bjp.bp.114.150961	No clinician-based PTSD-diagnosis based on a structured interview at inclusion
Burrichter, K., & Logan, W. (2023). The effectiveness of eye movement desensitization and reprocessing in the treatment of post-traumatic stress disorder: a randomized controlled trial. *Revista de Psiquiatria Clinica*, *50*(1), 63-70. https://doi.org/https://dx.doi.org/10.15761/0101-60830000000523	Not a randomized controlled trial
Burton, M. S., Cooper, A. A., Mello, P. G., Feeny, N. C., & Zoellner, L. A. (2021). Latent Profiles of Comorbid Depression as Predictors of PTSD Treatment Outcome. *Behavior therapy*, *52*(4), 970-981. https://doi.org/https://dx.doi.org/10.1016/j.beth.2020.12.005	Secondary analyses
Burton, M. S., Marks, E. H., Bedard-Gilligan, M. A., Feeny, N. C., & Zoellner, L. A. (2021). The effect of perceived life stress on posttraumatic stress disorder treatment outcome. *Journal of Traumatic Stress, 34*(6), 1219-1227. https://doi.org/https://dx.doi.org/10.1002/jts.22744	Secondary analyses
Butler, O., Willmund, G., Gleich, T., Gallinat, J., Kühn, S., & Zimmermann, P. (2018). Hippocampal gray matter increases following multimodal psychological treatment for combat-related post-traumatic stress disorder. *Brain Behav*, *8*(5), e00956. https://doi.org/10.1002/brb3.956	No clinician-based PTSD-diagnosis based on a structured interview at inclusion
Capone, C., Presseau, C., Saunders, E., Eaton, E., Hamblen, J., & McGovern, M. (2018). Is Integrated CBT Effective in Reducing PTSD Symptoms and Substance Use in Iraq and Afghanistan Veterans? Results from a Randomized Clinical Trial. *Cognitive therapy and research*, *42*(6), 735-746. https://doi.org/10.1007/s10608-018-9931-8	No dichotomous clinician-rated or self-reported response outcome related to change in PTSD symptoms

**Citation**	**Reason for exclusion**
Carlson, J. G., Chemtob, C. M., Rusnak, K., Hedlund, N. L., & Muraoka, M. Y. (1998). Eye movement desensitization and reprocessing (EDMR) treatment for combat-related posttraumatic stress disorder. *J Trauma Stress*, *11*(1), 3-24. https://doi.org/10.1023/a:1024448814268	Post-assessment more than six weeks after the end of treatment
Carlsson, J., Sonne, C., Vindbjerg, E., & Mortensen, E. L. (2018). Stress management versus cognitive restructuring in trauma-affected refugees-A pragmatic randomised study. *Psychiatry Res*, *266*, 116-123. https://doi.org/10.1016/j.psychres.2018.05.015	No dichotomous clinician-rated or self-reported response outcome related to change in PTSD symptoms
Classen, C., Koopman, C., Nevillmanning, K., & Spiegel, D. (2001). A preliminary report comparing trauma-focused and present-focused group therapy against a wait-listed condition among childhood sexual abuse survivors with PTSD. *Journal of Aggression, Maltreatment & Trauma*, *4*(2), 265-288. https://doi.org/10.1300/J146v04n02_12	No clinician-based PTSD-diagnosis based on a structured interview at inclusion
Claudat, K., Reilly, E. E., Convertino, A. D., Trim, J., Cusack, A., & Kaye, W. H. (2022). Integrating evidence-based PTSD treatment into intensive eating disorders treatment: a preliminary investigation. *Eating and weight disorders : EWD*, *27*(8), 3599-3607. https://doi.org/https://dx.doi.org/10.1007/s40519-022-01500-9	Not a randomized controlled trial
Coffey, S. F., Schumacher, J. A., Nosen, E., Littlefield, A. K., Henslee, A. M., Lappen, A., & Stasiewicz, P. R. (2016). Trauma-focused exposure therapy for chronic posttraumatic stress disorder in alcohol and drug dependent patients: A randomized controlled trial. *Psychol Addict Behav*, *30*(7), 778-790. https://doi.org/10.1037/adb0000201	Data do not allow conclusions on the number of non-responders per experimental and control group
Cottraux, J., Note, I., Yao, S. N., de Mey-Guillard, C., Bonasse, F., Djamoussian, D., Mollard, E., Note, B., & Chen, Y. (2008). Randomized controlled comparison of cognitive behavior therapy with Rogerian supportive therapy in chronic post-traumatic stress disorder: a 2-year follow-up. *Psychother Psychosom*, *77*(2), 101-110. https://doi.org/10.1159/000112887	No clinician-based PTSD-diagnosis based on a structured interview at inclusion
Cox, K. S., Wiener, D., Rauch, S. A. M., Tuerk, P. W., Wangelin, B., & Acierno, R. (2023). Individual symptom reduction and post-treatment severity: Varying levels of symptom amelioration in response to prolonged exposure for post-traumatic stress disorder. *Psychological Services*, *20*(1), 94-106. https://doi.org/https://dx.doi.org/10.1037/ser0000579	Secondary analyses
Crocker, L. D., Sullan, M. J., Jurick, S. M., Thomas, K. R., Davey, D. K., Hoffman, S. N., Twamley, E. W., & Jak, A. J. (2023). Baseline executive functioning moderates treatment-related changes in quality of life in veterans with posttraumatic stress disorder and comorbid traumatic brain injury. *Journal of Traumatic Stress*, *36*(1), 94-105. https://doi.org/https://dx.doi.org/10.1002/jts.22883	Secondary analyses
Davis, L. W., Luedtke, B. L., Monson, C., Siegel, A., Daggy, J. K., Yang, Z., Bair, M. J., Brustuen, B., & Ertl, M. (2021). Testing adaptations of cognitive-behavioral conjoint therapy for PTSD: A randomized controlled pilot study with veterans. *Couple and Family Psychology: Research and Practice*, *10*(2), 71-86. https://doi.org/10.1037/cfp0000148	Not a psychotherapeutic guideline-recommended intervention
Decker, K. P., Deaver, S. P., Abbey, V., Campbell, M., & Turpin, C. (2018). Quantitatively improved treatment outcomes for combat-associated PTSD with adjunctive art therapy: Randomized controlled trial. *Art Therapy*, *35*(4), 184-194. https://doi.org/10.1080/07421656.2018.1540822	No clinician-based PTSD-diagnosis based on a structured interview at inclusion
Dedert, E. A., Resick, P. A., Dennis, P. A., Wilson, S. M., Moore, S. D., & Beckham, J. C. (2019). Pilot trial of a combined cognitive processing therapy and smoking cessation treatment. *Journal of addiction medicine*, *13*(4), 322. https://doi.org/10.1097/ADM.0000000000000502	No dichotomous clinician-rated or self-reported response outcome related to change in PTSD symptoms
Devilly, G., Spence, S., & Rapee, R. (1998). Statistical and reliable change with eye movement desensitization and reprocessing: Treating trauma within a veteran population. *Behavior therapy*, *29*(3), 435-455. https://doi.org/10.1016/S0005-7894(98)80042-7	Did not apply DSM-IV, DSM-5, or ICD-10 criteria for PTSD
Devilly, G. J., & Spence, S. H. (1999). The relative efficacy and treatment distress of EMDR and a cognitive-behavior trauma treatment protocol in the amelioration of posttraumatic stress disorder. *J Anxiety Disord*, *13*(1-2), 131-157. https://doi.org/10.1016/s0887-6185(98)00044-9	No clinician-based PTSD-diagnosis based on a structured interview at inclusion
Dorrepaal, E., Thomaes, K., Smit, J. H., van Balkom, A. J., Veltman, D. J., Hoogendoorn, A. W., & Draijer, N. (2012). Stabilizing group treatment for complex posttraumatic stress disorder related to child abuse based on psychoeducation and cognitive behavioural therapy: a multisite randomized controlled trial. *Psychother Psychosom*, *81*(4), 217-225. https://doi.org/10.1159/000335044	Not a psychotherapeutic guideline-recommended intervention

Citation	Reason for exclusion
Duffy, M., Gillespie, K., & Clark, D. M. (2007). Post-traumatic stress disorder in the context of terrorism and other civil conflict in Northern Ireland: randomised controlled trial. *BMJ, 334*(7604), 1147. https://doi.org/10.1136/bmj.39021.846852.BE	No clinician-based PTSD-diagnosis based on a structured interview at inclusion
Duran, É., Corchs, F., Vianna, A., Araujo, A., Del Real, N., Silva, C., Ferreira, A., Francez, P., Godoi, C., Silveira, H., Matsumoto, L., Gebara, C., Neto, T., Chilvarquer, R., de Siqueira, L., Bernik, M., & Neto, F. (2021). A randomized clinical trial to assess the efficacy of trial-based cognitive therapy compared to prolonged exposure for post-traumatic stress disorder: preliminary findings. *CNS Spectrums, 26*(4), 427-434. https://doi.org/https://doi.org/10.1017/S1092852920001455	No clinician-based PTSD-diagnosis based on a structured interview at inclusion
Echeburúa, E., de Corral, P., Sarasua, B., & Zubizarreta, I. (1996). Treatment of acute posttraumatic stress disorder in rape victims: An experimental study. *Journal of anxiety disorders, 10*(3), 185-199. https://doi.org/10.1016/0887-6185(96)89842-2	Participants under 17 years of age
Edgar, N., Bennett, A., Dunn, N., MacLean, S., Hatcher, S., N.E, E., A, B., N.S, D., & S.E, M. (2022). Feasibility and acceptability of Narrative Exposure Therapy to treat individuals with PTSD who are homeless or vulnerably housed: a pilot randomized controlled trial. *Pilot and Feasibility Studies, 8*(1), 83-83. https://doi.org/https://dx.doi.org/10.1186/s40814-022-01043-x	No dichotomous clinician-rated or self-reported response outcome related to change in PTSD symptoms
Eskici, H. S., Hinton, D. E., Jalal, B., Yurtbakan, T., & Acarturk, C. (2023). Culturally adapted cognitive behavioral therapy for Syrian refugee women in Turkey: A randomized controlled trial. *Psychological trauma : theory, research, practice and policy, 15*(2), 189-198. https://doi.org/https://dx.doi.org/10.1037/tra0001138	No clinician-based PTSD-diagnosis based on a structured interview at inclusion
Fan, Y., Shi, Y., Zhang, J., Sun, D., Wang, X., Fu, G., Mo, D., Wen, J., Xiao, X., & Kong, L. (2021). The effects of narrative exposure therapy on COVID-19 patients with post-traumatic stress symptoms: A randomized controlled trial. *Journal of Affective Disorders, 293*, 141-147. https://doi.org/https://dx.doi.org/10.1016/j.jad.2021.06.019	No clinician-based PTSD-diagnosis based on a structured interview at inclusion
Feurer, C., Francis, J., Ajilore, O., Craske, M. G., Phan, K. L., & Klumpp, H. (2021). Emotion Regulation and Repetitive Negative Thinking Before and After CBT and SSRI Treatment of Internalizing Psychopathologies. *Cognitive Therapy and Research, 45*(6), 1064-1076. https://doi.org/https://dx.doi.org/10.1007/s10608-021-10222-8	No full PTSD-diagnosis required for inclusion
Foa, E. B., Bredemeier, K., Acierno, R., Rosenfield, D., Muzzy, W., Tuerk, P. W., Zandberg, L. J., Hart, S., Young-McCaughan, S., Peterson, A. L., & McLean, C. P. (2022). The efficacy of 90-min versus 60-min sessions of prolonged exposure for PTSD: A randomized controlled trial in active-duty military personnel. *Journal of Consulting and Clinical Psychology, 90*(6), 503-512. https://doi.org/https://dx.doi.org/10.1037/ccp0000739	No dichotomous clinician-rated or self-reported response outcome related to change in PTSD symptoms
Foa, E. B., Dancu, C. V., Hembree, E. A., Jaycox, L. H., Meadows, E. A., & Street, G. P. (1999). A comparison of exposure therapy, stress inoculation training, and their combination for reducing posttraumatic stress disorder in female assault victims. *J Consult Clin Psychol*, *67*(2), 194-200. https://doi.org/10.1037//0022-006x.67.2.194	Did not apply DSM-IV, DSM-5, or ICD-10 criteria for PTSD
Foa, E. B., Hembree, E. A., Cahill, S. P., Rauch, S. A., Riggs, D. S., Feeny, N. C., & Yadin, E. (2005). Randomized trial of prolonged exposure for posttraumatic stress disorder with and without cognitive restructuring: outcome at academic and community clinics. *J Consult Clin Psychol*, *73*(5), 953-964. https://doi.org/10.1037/0022-006x.73.5.953	Post-assessment more than six weeks after the end of treatment
Foa, E. B., Rothbaum, B. O., Riggs, D. S., & Murdock, T. B. (1991). Treatment of posttraumatic stress disorder in rape victims: a comparison between cognitive-behavioral procedures and counseling. *J Consult Clin Psychol*, *59*(5), 715-723. https://doi.org/10.1037//0022-006x.59.5.715	Did not apply DSM-IV, DSM-5, or ICD-10 criteria for PTSD
Fonzo, G. A., Goodkind, M. S., Oathes, D. J., Zaiko, Y. V., Harvey, M., Peng, K. K., Weiss, M. E., Thompson, A. L., Zack, S. E., Lindley, S. E., Arnow, B. A., Jo, B., Gross, J. J., Rothbaum, B. O., & Etkin, A. (2017). PTSD Psychotherapy Outcome Predicted by Brain Activation During Emotional Reactivity and Regulation. *Am J Psychiatry*, *174*(12), 1163-1174. https://doi.org/10.1176/appi.ajp.2017.16091072	No dichotomous clinician-rated or self-reported response outcome related to change in PTSD symptoms
Frommberger, U., Stieglitz, R. D., Nyberg, E., Richter, H., Novelli-Fischer, U., Angenendt, J., Zaninelli, R., & Berger, M. (2004). Comparison between paroxetine and behaviour therapy in patients with posttraumatic stress disorder (PTSD): A pilot study. *Int J Psychiatry Clin Pract*, *8*(1), 19-23. https://doi.org/10.1080/13651500310004803	Did not apply DSM-IV, DSM-5, or ICD-10 criteria for PTSD

Citation	Reason for exclusion
Frueh, B. C., Monnier, J., Yim, E., Grubaugh, A. L., Hamner, M. B., & Knapp, R. G. (2007). A randomized trial of telepsychiatry for post-traumatic stress disorder. *J Telemed Telecare*, *13*(3), 142-147. https://doi.org/10.1258/135763307780677604	No dichotomous clinician-rated or self-reported response outcome related to change in PTSD symptoms
Galovski, T. E., Blain, L. M., Mott, J. M., Elwood, L., & Houle, T. (2012). Manualized therapy for PTSD: flexing the structure of cognitive processing therapy. *J Consult Clin Psychol*, *80*(6), 968-981. https://doi.org/10.1037/a0030600	Data do not allow conclusions on the number of non-responders per experimental and control group
Galovski, T. E., Harik, J. M., Blain, L. M., Elwood, L., Gloth, C., & Fletcher, T. D. (2016). Augmenting cognitive processing therapy to improve sleep impairment in PTSD: A randomized controlled trial. *J Consult Clin Psychol*, *84*(2), 167-177. https://doi.org/10.1037/ccp0000059	No dichotomous clinician-rated or self-reported response outcome related to change in PTSD symptoms
Gamito, P., Oliveira, J., Rosa, P., Morais, D., Duarte, N., Oliveira, S., & Saraiva, T. (2010). PTSD elderly war veterans: a clinical controlled pilot study. *Cyberpsychol Behav Soc Netw*, *13*(1), 43-48. https://doi.org/10.1089/cyber.2009.0237	No dichotomous clinician-rated or self-reported response outcome related to change in PTSD symptoms
Gersons, B. P., Carlier, I. V., Lamberts, R. D., & van der Kolk, B. A. (2000). Randomized clinical trial of brief eclectic psychotherapy for police officers with posttraumatic stress disorder. *J Trauma Stress*, *13*(2), 333-347. https://doi.org/10.1023/a:1007793803627	Did not apply DSM-IV, DSM-5, or ICD-10 criteria for PTSD
Ghafoori, B., Hansen, M. C., Garibay, E., & Korosteleva, O. (2017). Feasibility of Training Frontline Therapists in Prolonged Exposure: A Randomized Controlled Pilot Study of Treatment of Complex Trauma in Diverse Victims of Crime and Violence. *J Nerv Ment Dis*, *205*(4), 283-293. https://doi.org/10.1097/nmd.0000000000000659	No dichotomous clinician-rated or self-reported response outcome related to change in PTSD symptoms
Hahn, C. K., Jarnecke, A. M., Calhoun, C., Melkonian, A., Flanagan, J. C., & Back, S. E. (2022). Sexual harassment and assault during deployment: Associations with treatment outcomes among Veterans with co-occurring PTSD and SUD. *Military Psychology, 34*(1), 12-22. https://doi.org/10.1080/08995605.2021.1964901	No dichotomous clinician-rated or self-reported response outcome related to change in PTSD symptoms
Haller, H., Mitzinger, D., & Cramer, H. (2023). The integration of yoga breathing techniques in cognitive behavioral therapy for post-traumatic stress disorder: A pragmatic randomized controlled trial. *Frontiers in Psychiatry*, *14*, 1101046. https://doi.org/https://dx.doi.org/10.3389/fpsyt.2023.1101046	No clinician-based PTSD-diagnosis based on a structured interview at inclusion
Held, P., Kovacevic, M., Petrey, K., Meade, E. A., Pridgen, S., Montes, M., Werner, B., Miller, M. L., Smith, D. L., Kaysen, D., & Karnik, N. S. (2022). Treating posttraumatic stress disorder at home in a single week using 1-week virtual massed cognitive processing therapy. *Journal of Traumatic Stress, 35*(4), 1215-1225. https://doi.org/https://dx.doi.org/10.1002/jts.22831	No full PTSD-diagnosis required for inclusion
Hien, D. A., Cohen, L. R., Miele, G. M., Litt, L. C., & Capstick, C. (2004). Promising treatments for women with comorbid PTSD and substance use disorders. *Am J Psychiatry*, *161*(8), 1426-1432. https://doi.org/10.1176/appi.ajp.161.8.1426	No full PTSD-diagnosis required for inclusion
Hien, D. A., Wells, E. A., Jiang, H., Suarez-Morales, L., Campbell, A. N., Cohen, L. R., Miele, G. M., Killeen, T., Brigham, G. S., Zhang, Y., Hansen, C., Hodgkins, C., Hatch-Maillette, M., Brown, C., Kulaga, A., Kristman-Valente, A., Chu, M., Sage, R., Robinson, J. A., . . . Nunes, E. V. (2009). Multisite randomized trial of behavioral interventions for women with co-occurring PTSD and substance use disorders. *J Consult Clin Psychol*, *77*(4), 607-619. https://doi.org/10.1037/a0016227	No full PTSD-diagnosis required for inclusion
Hijazi, A. M., Lumley, M. A., Ziadni, M. S., Haddad, L., Rapport, L. J., & Arnetz, B. B. (2014). Brief narrative exposure therapy for posttraumatic stress in Iraqi refugees: a preliminary randomized clinical trial. *J Trauma Stress*, *27*(3), 314-322. https://doi.org/10.1002/jts.21922	No full PTSD-diagnosis required for inclusion
Hinton, D. E., Hofmann, S. G., Pollack, M. H., & Otto, M. W. (2009). Mechanisms of efficacy of CBT for Cambodian refugees with PTSD: improvement in emotion regulation and orthostatic blood pressure response. *CNS Neurosci Ther*, *15*(3), 255-263. https://doi.org/10.1111/j.1755-5949.2009.00100.x	No dichotomous clinician-rated or self-reported response outcome related to change in PTSD symptoms
Hoffart, A., Øktedalen, T., Langkaas, T. F., & Wampold, B. E. (2013). Alliance and outcome in varying imagery procedures for PTSD: a study of within-person processes. *J Couns Psychol*, *60*(4), 471-482. https://doi.org/10.1037/a0033604	No dichotomous clinician-rated or self-reported response outcome related to change in PTSD symptoms

Citation	Reason for exclusion
Hunt, C., Park, J., Bomyea, J., & Colvonen, P. J. (2023). Sleep efficiency predicts improvements in fear extinction and PTSD symptoms during prolonged exposure for veterans with comorbid insomnia. *Psychiatry Research*, *324*, 115216. https://doi.org/https://dx.doi.org/10.1016/j.psychres.2023.115216	No clinician-based PTSD-diagnosis based on a structured interview at inclusion
Ivarsson, D., Blom, M., Hesser, H., Carlbring, P., Enderby, P., Nordberg, R., & Andersson, G. (2014). Guided internet-delivered cognitive behavior therapy for post-traumatic stress disorder: a randomized controlled trial. *Internet interventions*, *1*(1), 33-40. https://doi.org/10.1016/j.invent.2014.03.002	Not a psychotherapeutic guideline recommended intervention
Jacob, N., Neuner, F., Maedl, A., Schaal, S., & Elbert, T. (2014). Dissemination of psychotherapy for trauma spectrum disorders in postconflict settings: a randomized controlled trial in Rwanda. *Psychother Psychosom*, *83*(6), 354-363. https://doi.org/10.1159/000365114	Post-assessment more than six weeks after the end of treatment
Jak, A. J., Jurick, S., Crocker, L. D., Sanderson-Cimino, M., Aupperle, R., Rodgers, C. S., Thomas, K. R., Boyd, B., Norman, S. B., Lang, A. J., Keller, A. V., Schiehser, D. M., & Twamley, E. W. (2019). SMART-CPT for veterans with comorbid post-traumatic stress disorder and history of traumatic brain injury: a randomised controlled trial. *J Neurol Neurosurg Psychiatry*, *90*(3), 333-341. https://doi.org/10.1136/jnnp-2018-319315	No dichotomous clinician-rated or self-reported response outcome related to change in PTSD symptoms
Jamshidi, F., Rajabi, S., & Dehghani, Y. (2021). How to heal their psychological wounds? Effectiveness of EMDR therapy on post-traumatic stress symptoms, mind-wandering and suicidal ideation in Iranian child abuse victims. *Counselling and Psychotherapy Research*, *21*(2), 412-421. https://doi.org/https://doi.org/10.1002/capr.12339	No clinician-based PTSD-diagnosis based on a structured interview at inclusion
Jensen, J. A. (1994). An investigation of eye movement desensitization and reprocessing (EMD/R) as a treatment for posttraumatic stress disorder (PTSD) symptoms of Vietnam combat veterans. *Behavior therapy*, *25*(2), 311-325. https://doi.org/10.1016/S0005-7894(05)80290-4	Did not apply DSM-IV, DSM-5, or ICD-10 criteria for PTSD
Katz, L., Douglas, S., Zaleski, K., Williams, J., Huffman, C., & Cojucar, G. (2014). Comparing Holographic Reprocessing and Prolonged Exposure for Women Veterans with Sexual Trauma: A Pilot Randomized Trial. *Journal of Contemporary Psychotherapy*, *44*(1). https://doi.org/10.1007/s10879-013-9248-6	No full PTSD-diagnosis required for inclusion
Keane, T. M., Fairbank, J. A., Caddell, J. M., & Zimering, R. T. (1989). Implosive (flooding) therapy reduces symptoms of PTSD in Vietnam combat veterans. *Behavior therapy*, *20*(2), 245-260. https://doi.org/10.1016/S0005-7894(89)80072-3	Did not apply DSM-IV, DSM-5, or ICD-10 criteria for PTSD
Kehle-Forbes, S. M., Chen, S., Polusny, M. A., Lynch, K. G., Koffel, E., Ingram, E., Foa, E. B., Van Horn, D. H. A., Drapkin, M. L., Yusko, D. A., & Oslin, D. W. (2019). A randomized controlled trial evaluating integrated versus phased application of evidence-based psychotherapies for military veterans with comorbid PTSD and substance use disorders. *Drug Alcohol Depend*, *205*, 107647. https://doi.org/10.1016/j.drugalcdep.2019.107647	No dichotomous clinician-rated or self-reported response outcome related to change in PTSD symptoms
Khan, A., Ullah, F., Abid, O., & Awan, K. H. (2021). Efficacy of cognitive behavioral therapy in post-traumatic stress disorder among spinal cord injury patients: A randomized controlled pilot study. *Journal of Evidence-Based Psychotherapies, 21*(2), 143-162. https://doi.org/10.24193/jebp.2021.2.16	No dichotomous clinician-rated or self-reported response outcome related to change in PTSD symptoms
Kleindienst, N., Steil, R., Priebe, K., Muller-Engelmann, M., Biermann, M., Fydrich, T., Schmahl, C., & Bohus, M. (2021). Treating adults with a dual diagnosis of borderline personality disorder and posttraumatic stress disorder related to childhood abuse: Results from a randomized clinical trial. *Journal of Consulting and Clinical Psychology*, *89*(11), 925-936. https://doi.org/https://dx.doi.org/10.1037/ccp0000687	No dichotomous clinician-rated or self-reported response outcome related to change in PTSD symptoms
Knaevelsrud, C., Böttche, M., Pietrzak, R. H., Freyberger, H. J., & Kuwert, P. (2017). Efficacy and Feasibility of a Therapist-Guided Internet-Based Intervention for Older Persons with Childhood Traumatization: A Randomized Controlled Trial. *Am J Geriatr Psychiatry*, *25*(8), 878-888. https://doi.org/10.1016/j.jagp.2017.02.024	No full PTSD-diagnosis required for inclusion
Knaevelsrud, C., Brand, J., Lange, A., Ruwaard, J., & Wagner, B. (2015). Web-based psychotherapy for posttraumatic stress disorder in war-traumatized Arab patients: randomized controlled trial. *J Med Internet Res*, *17*(3), e71. https://doi.org/10.2196/jmir.3582	No clinician-based PTSD-diagnosis based on a structured interview at inclusion
Koebach, A., Carleial, S., Elbert, T., Schmitt, S., & Robjant, K. (2021). Treating trauma and aggression with narrative exposure therapy in former child and adult soldiers: A randomized controlled trial in Eastern DR Congo. *Journal of Consulting and Clinical Psychology*, *89*(3), 143.	No dichotomous clinician-rated or self-reported response outcome related to change in PTSD symptoms

Citation	Reason for exclusion
Koochaki, M., Mahmoodi, Z., Esmaelzadeh–Saeieh, S., Kabir, K., & Dolatian, M. (2017). Effects of Cognitive-Behavioral Counseling on Posttraumatic Stress Disorder in Mothers with Infants Hospitalized at Neonatal Intensive Care Units: A Randomized Controlled Trial. *Iranian Journal of Psychiatry and Behavioral Sciences*, *12*(4). https://doi.org/10.5812/ijpbs.65159	No clinician-based PTSD-diagnosis based on a structured interview at inclusion
Krupnick, J. L., Green, B. L., Amdur, R., Alaoui, A., Belouali, A., Roberge, E., Cueva, D., Roberts, M., Melnikoff, E., & Dutton, M. A. (2017). An Internet-based writing intervention for PTSD in veterans: A feasibility and pilot effectiveness trial. *Psychol Trauma*, *9*(4), 461-470. https://doi.org/10.1037/tra0000176	No clinician-based PTSD-diagnosis based on a structured interview at inclusion
Kullberg, M.-L. J., Schoorl, M., Oprel, D. A. C., Hoeboer, C. M., Smit, F., van der Does, W., de Kleine, R. A., van Minnen, A., & van den Hout, W. (2023). Exposure-based treatments for childhood abuse-related post-traumatic stress disorder in adults: a health-economic evaluation. *European Journal of Psychotraumatology*, *14*(1), 2171752. https://doi.org/https://dx.doi.org/10.1080/20008066.2023.2171752	Secondary analyses
Larsen, S. E., Mackintosh, M.-A., La Bash, H., Evans, W. R., Suvak, M. K., Shields, N., Lane, J. E. M., Sijercic, I., Monson, C. M., & Wiltsey Stirman, S. (2022). Temporary PTSD symptom increases among individuals receiving CPT in a hybrid effectiveness-implementation trial: Potential predictors and association with overall symptom change trajectory. *Psychological trauma : theory, research, practice and policy*, *14*(5), 853-861. https://doi.org/https://dx.doi.org/10.1037/tra0000545	Secondary analyses
Lee, C., Gavriel, H., Drummond, P., Richards, J., & Greenwald, R. (2002). Treatment of PTSD: stress inoculation training with prolonged exposure compared to EMDR. *J Clin Psychol*, *58*(9), 1071-1089. https://doi.org/10.1002/jclp.10039	Did not apply DSM-IV, DSM-5, or ICD-10 criteria for PTSD
Lee, D. J., Marx, B. P., Thompson-Hollands, J., Gallagher, M. W., Resick, P. A., & Sloan, D. M. (2021). The temporal sequence of change in PTSD symptoms and hypothesized mediators in Cognitive Processing Therapy and Written Exposure Therapy for PTSD. *Behaviour Research and Therapy*, *144*, 103918-103918. https://doi.org/https://dx.doi.org/10.1016/j.brat.2021.103918	Secondary analyses
Lely, J. C. G., Ter Heide, F. J. J., Moerbeek, M., Knipscheer, J. W., & Kleber, R. J. (2022). Psychopathology and resilience in older adults with posttraumatic stress disorder: a randomized controlled trial comparing narrative exposure therapy and present-centered therapy. *European Journal of Psychotraumatology*, *13*(1), 2022277-2022277. https://doi.org/https://dx.doi.org/10.1080/20008198.2021.2022277	No clinician-based PTSD-diagnosis based on a structured interview at inclusion
Lewis, N. V., Gregory, A., Feder, G. S., Angill-Williams, A., Bates, S., Glynn, J., Halliwell, G., Hawcroft, C., Kessler, D., Lawton, M., Leach, R., Millband, S., Pitt, K., Zammit, S., & Malpass, A. (2023). Trauma-specific mindfulness-based cognitive therapy for women with post-traumatic stress disorder and a history of domestic abuse: intervention refinement and a randomised feasibility trial (coMforT study). *Pilot and Feasibility Studies*, *9*(1), 112. https://doi.org/https://dx.doi.org/10.1186/s40814-023-01335-w	Secondary analyses
Lin, Y., Lv, W., Xu, J., Jiang, Y., & Chen, Z. (2022). Effectiveness of Cognitive Behavior Therapy Combined with Eye Movement Desensitization and Reprocessing on Psychological Problems and Life Quality in Patients' Postfacial Trauma. *Computational and mathematical methods in medicine*, *2022*, 7822847-7822847. https://doi.org/https://dx.doi.org/10.1155/2022/7822847	No clinician-based PTSD-diagnosis based on a structured interview at inclusion
Liu, L., Thorp, S. R., Moreno, L., Wells, S. Y., Glassman, L. H., Busch, A. C., Zamora, T., Rodgers, C. S., Allard, C. B., Morland, L. A., & Agha, Z. (2020). Videoconferencing psychotherapy for veterans with PTSD: Results from a randomized controlled non-inferiority trial. *J Telemed Telecare*, *26*(9), 507-519. https://doi.org/10.1177/1357633x19853947	No dichotomous clinician-rated or self-reported response outcome related to change in PTSD symptoms
LoSavio, S. T., Hale, W. J., Moring, J. C., Blankenship, A. E., Dondanville, K. A., Wachen, J. S., Mintz, J., Peterson, A. L., Litz, B. T., Young-McCaughan, S., Yarvis, J. S., & Resick, P. A. (2021). Efficacy of individual and group cognitive processing therapy for military personnel with and without child abuse histories. *Journal of Consulting and Clinical Psychology*, *89*(5), 476-482. https://doi.org/https://dx.doi.org/10.1037/ccp0000641	Secondary analyses
Lyons, R., Helm, J., Luciano, M., Haller, M., & Norman, S. B. (2023). The role of posttraumatic cognitions in integrated treatments for co-occurring posttraumatic stress disorder and alcohol use disorder. *Psychological Trauma: Theory, Research, Practice, and Policy*. https://doi.org/10.1037/tra0001540	Secondary analyses

Citation	Reason for exclusion
Maieritsch, K. P., Smith, T. L., Hessinger, J. D., Ahearn, E. P., Eickhoff, J. C., & Zhao, Q. (2016). Randomized controlled equivalence trial comparing videoconference and in person delivery of cognitive processing therapy for PTSD. *J Telemed Telecare*, *22*(4), 238-243. https://doi.org/10.1177/1357633x15596109	No dichotomous clinician-rated or self-reported response outcome related to change in PTSD symptoms
Marks, I., Lovell, K., Noshirvani, H., Livanou, M., & Thrasher, S. (1998). Treatment of posttraumatic stress disorder by exposure and/or cognitive restructuring: a controlled study. *Arch Gen Psychiatry*, *55*(4), 317-325. https://doi.org/10.1001/archpsyc.55.4.317	Did not apply DSM-IV, DSM-5, or ICD-10 criteria for PTSD
McGeary, D. D., Resick, P. A., Penzien, D. B., McGeary, C. A., Houle, T. T., Eapen, B. C., Jaramillo, C. A., Nabity, P. S., Reed, D. E., 2nd, Moring, J. C., Bira, L. M., Hansen, H. R., Young-McCaughan, S., Cobos, B. A., Mintz, J., Keane, T. M., & Peterson, A. L. (2022). Cognitive Behavioral Therapy for Veterans With Comorbid Posttraumatic Headache and Posttraumatic Stress Disorder Symptoms: A Randomized Clinical Trial. *JAMA neurology*, *79*(8), 746-757. https://doi.org/https://dx.doi.org/10.1001/jamaneurol.2022.1567	No full PTSD-diagnosis required for inclusion
McGovern, M. P., Lambert-Harris, C., Xie, H., Meier, A., McLeman, B., & Saunders, E. (2015). A randomized controlled trial of treatments for co-occurring substance use disorders and post-traumatic stress disorder. *Addiction*, *110*(7), 1194-1204. https://doi.org/10.1111/add.12943	No dichotomous clinician-rated or self-reported response outcome related to change in PTSD symptoms
McGuire Stanbury, T. M., Drummond, P. D., Laugharne, J., Kullack, C., & Lee, C. W. (2020). Comparative efficiency of EMDR and prolonged exposure in treating posttraumatic stress disorder: A randomized trial. *Journal of EMDR Practice and Research*, *14*(1), 2-12. https://doi.org/10.1891/1933-3196.14.1.2	No dichotomous clinician-rated or self-reported response outcome related to change in PTSD symptoms
McLay, R. N., Wood, D. P., Webb-Murphy, J. A., Spira, J. L., Wiederhold, M. D., Pyne, J. M., & Wiederhold, B. K. (2011). A randomized, controlled trial of virtual reality-graded exposure therapy for post-traumatic stress disorder in active duty service members with combat-related post-traumatic stress disorder. *Cyberpsychol Behav Soc Netw*, *14*(4), 223-229. https://doi.org/10.1089/cyber.2011.0003	Not a psychotherapeutic guideline recommended intervention
Mills, K. L., Teesson, M., Back, S. E., Brady, K. T., Baker, A. L., Hopwood, S., Sannibale, C., Barrett, E. L., Merz, S., Rosenfeld, J., & Ewer, P. L. (2012). Integrated exposure-based therapy for co-occurring posttraumatic stress disorder and substance dependence: a randomized controlled trial. *Jama*, *308*(7), 690-699. https://doi.org/10.1001/jama.2012.9071	Post-assessment more than six weeks after the end of treatment
Morath, J., Gola, H., Sommershof, A., Hamuni, G., Kolassa, S., Catani, C., Adenauer, H., Ruf-Leuschner, M., Schauer, M., Elbert, T., Groettrup, M., & Kolassa, I. T. (2014). The effect of trauma-focused therapy on the altered T cell distribution in individuals with PTSD: evidence from a randomized controlled trial. *J Psychiatr Res*, *54*, 1-10. https://doi.org/10.1016/j.jpsychires.2014.03.016	Post-assessment more than six weeks after the end of treatment
Moreira, A., Moreira, A. C., & Rocha, J. C. (2022). Randomized Controlled Trial: Cognitive-Narrative Therapy for IPV Victims. *Journal of Interpersonal Violence*, *37*(5-6), NP2998-NP3014. https://doi.org/https://dx.doi.org/10.1177/0886260520943719	No clinician-based PTSD-diagnosis based on a structured interview at inclusion
Morland, L. A., Mackintosh, M. A., Greene, C. J., Rosen, C. S., Chard, K. M., Resick, P., & Frueh, B. C. (2014). Cognitive processing therapy for posttraumatic stress disorder delivered to rural veterans via telemental health: a randomized noninferiority clinical trial. *J Clin Psychiatry*, *75*(5), 470-476. https://doi.org/10.4088/JCP.13m08842	Data do not allow conclusions on the number of non-responders per experimental and control group
Morland, L. A., Mackintosh, M. A., Rosen, C. S., Willis, E., Resick, P., Chard, K., & Frueh, B. C. (2015). Telemedicine versus in-person delivery of cognitive processing therapy for women with posttraumatic stress disorder: a randomized noninferiority trial. *Depress Anxiety*, *32*(11), 811-820. https://doi.org/10.1002/da.22397	No dichotomous clinician-rated or self-reported response outcome related to change in PTSD symptoms
Nacasch, N., Foa, E. B., Huppert, J. D., Tzur, D., Fostick, L., Dinstein, Y., Polliack, M., & Zohar, J. (2011). Prolonged exposure therapy for combat- and terror-related posttraumatic stress disorder: a randomized control comparison with treatment as usual. *J Clin Psychiatry*, *72*(9), 1174-1180. https://doi.org/10.4088/JCP.09m05682blu	No dichotomous clinician-rated or self-reported response outcome related to change in PTSD symptoms
Nacasch, N., Huppert, J. D., Su, Y. J., Kivity, Y., Dinshtein, Y., Yeh, R., & Foa, E. B. (2015). Are 60-minute prolonged exposure sessions with 20-minute imaginal exposure to traumatic memories sufficient to successfully treat PTSD? A randomized noninferiority clinical trial. *Behav Ther*, *46*(3), 328-341. https://doi.org/10.1016/j.beth.2014.12.002	No dichotomous clinician-rated or self-reported response outcome related to change in PTSD symptoms

Citation	Reason for exclusion
Neuner, F., Kurreck, S., Ruf, M., Odenwald, M., Elbert, T., & Schauer, M. (2010). Can asylum-seekers with posttraumatic stress disorder be successfully treated? A randomized controlled pilot study. *Cogn Behav Ther*, *39*(2), 81-91. https://doi.org/10.1080/16506070903121042	No clinician-based PTSD-diagnosis based on a structured interview at inclusion
Neuner, F., Onyut, P. L., Ertl, V., Odenwald, M., Schauer, E., & Elbert, T. (2008). Treatment of posttraumatic stress disorder by trained lay counselors in an African refugee settlement: a randomized controlled trial. *J Consult Clin Psychol*, *76*(4), 686-694. https://doi.org/10.1037/0022-006x.76.4.686	No clinician-based PTSD-diagnosis based on a structured interview at inclusion
Norman, S. B., Trim, R., Haller, M., Davis, B. C., Myers, U. S., Colvonen, P. J., Blanes, E., Lyons, R., Siegel, E. Y., Angkaw, A. C., Norman, G. J., & Mayes, T. (2019). Efficacy of Integrated Exposure Therapy vs Integrated Coping Skills Therapy for Comorbid Posttraumatic Stress Disorder and Alcohol Use Disorder: A Randomized Clinical Trial. *JAMA Psychiatry*, *76*(8), 791-799. https://doi.org/10.1001/jamapsychiatry.2019.0638	No full PTSD-diagnosis required for inclusion
Oprel, D. A. C., Hoeboer, C. M., Schoorl, M., de Kleine, R. A., Cloitre, M., Wigard, I. G., van Minnen, A., & van der Does, W. (2021). Effect of Prolonged Exposure, intensified Prolonged Exposure and STAIR+Prolonged Exposure in patients with PTSD related to childhood abuse: a randomized controlled trial. *European Journal of Psychotraumatology*, *12*(1), 1851511-1851511. https://doi.org/https://dx.doi.org/10.1080/20008198.2020.1851511	No dichotomous clinician-rated or self-reported response outcome related to change in PTSD symptoms
Orang, T., Ayoughi, S., Moran, J. K., Ghaffari, H., Mostafavi, S., Rasoulian, M., & Elbert, T. (2018). The efficacy of narrative exposure therapy in a sample of Iranian women exposed to ongoing intimate partner violence-A randomized controlled trial. *Clin Psychol Psychother*, *25*(6), 827-841. https://doi.org/10.1002/cpp.2318	Post-assessment more than six weeks after the end of treatment
Park, J., Hunt, C., Abirgas, K., Bomyea, J., & Colvonen, P. J. (2023). Veterans who focus on sexual assault trauma show slower between-session habituation and symptom reduction during prolonged exposure treatment for posttraumatic stress disorder. *Psychological Trauma: Theory, Research, Practice, and Policy*. https://doi.org/10.1037/tra0001536	Secondary analyses
Paunovic, N., & Ost, L. G. (2001). Cognitive-behavior therapy vs exposure therapy in the treatment of PTSD in refugees. *Behav Res Ther*, *39*(10), 1183-1197. https://doi.org/10.1016/s0005-7967(00)00093-0	No dichotomous clinician-rated or self-reported response outcome related to change in PTSD symptoms
Perez-Dandieu, B., & Tapia, G. (2014). Treating Trauma in Addiction with EMDR: A Pilot Study. *J Psychoactive Drugs*, *46*(4), 303-309. https://doi.org/10.1080/02791072.2014.921744	No clinician-based PTSD-diagnosis based on a structured interview at inclusion
Pigeon, W. R., Crean, H. F., Cerulli, C., Gallegos, A. M., Bishop, T. M., & Heffner, K. L. (2022). A Randomized Clinical Trial of Cognitive-Behavioral Therapy for Insomnia to Augment Posttraumatic Stress Disorder Treatment in Survivors of Interpersonal Violence. *Psychotherapy and Psychosomatics*, *91*(1), 50-62. https://doi.org/https://dx.doi.org/10.1159/000517862	No full PTSD-diagnosis required for inclusion
Polak, A. R., Witteveen, A. B., Denys, D., & Olff, M. (2015). Breathing biofeedback as an adjunct to exposure in cognitive behavioral therapy hastens the reduction of PTSD symptoms: a pilot study. *Appl Psychophysiol Biofeedback*, *40*(1), 25-31. https://doi.org/10.1007/s10484-015-9268-y	No dichotomous clinician-rated or self-reported response outcome related to change in PTSD symptoms
Power, K., McGoldrick, T., Brown, K., Buchanan, R., Sharp, D., Swanson, V., & Karatzias, A. (2002). A controlled comparison of eye movement desensitization and reprocessing versus exposure plus cognitive restructuring versus waiting list in the treatment of post-traumatic stress disorder. *Clinical Psychology & Psychotherapy*, *9*(5), 299-318. https://doi.org/10.1002/cpp.341	Did not apply DSM-IV, DSM-5, or ICD-10 criteria for PTSD
Powers, M. B., Medina, J. L., Burns, S., Kauffman, B. Y., Monfils, M., Asmundson, G. J., Diamond, A., McIntyre, C., & Smits, J. A. (2015). Exercise Augmentation of Exposure Therapy for PTSD: Rationale and Pilot Efficacy Data. *Cogn Behav Ther*, *44*(4), 314-327. https://doi.org/10.1080/16506073.2015.1012740	No dichotomous clinician-rated or self-reported response outcome related to change in PTSD symptoms
Rauch, S. A. M., Kim, H. M., Acierno, R., Ragin, C., Wangelin, B., Blitch, K., Muzzy, W., Hart, S., & Zivin, K. (2023). Improving function through primary care treatment of posttraumatic stress disorder study outcomes: A randomized controlled trial of prolonged exposure for primary care in veterans. *Families, Systems, & Health*. https://doi.org/10.1037/fsh0000823	No clinician-based PTSD-diagnosis based on a structured interview at inclusion

Citation	Reason for exclusion
Resick, P. A., Wachen, J. S., Dondanville, K. A., LoSavio, S. T., Young-McCaughan, S., Yarvis, J. S., Pruiksma, K. E., Blankenship, A., Jacoby, V., Peterson, A. L., & Mintz, J. (2021). Variable-length cognitive processing therapy for posttraumatic stress disorder in active duty military: Outcomes and predictors. *Behaviour Research and Therapy*, *141*. https://doi.org/10.1016/j.brat.2021.103846	Not a randomized controlled trial
Robjant, K., Koebach, A., Schmitt, S., Chibashimba, A., Carleial, S., & Elbert, T. (2019). The treatment of posttraumatic stress symptoms and aggression in female former child soldiers using adapted Narrative Exposure therapy - a RCT in Eastern Democratic Republic of Congo. *Behav Res Ther*, *123*, 103482. https://doi.org/10.1016/j.brat.2019.103482	Post-assessment more than six weeks after the end of treatment
Rothbaum, B. O. (1997). A controlled study of eye movement desensitization and reprocessing in the treatment of posttraumatic stress disordered sexual assault victims. *Bull Menninger Clin*, *61*(3), 317-334.	Did not apply DSM-IV, DSM-5, or ICD-10 criteria for PTSD
Sannibale, C., Teesson, M., Creamer, M., Sitharthan, T., Bryant, R. A., Sutherland, K., Taylor, K., Bostock-Matusko, D., Visser, A., & Peek-O'Leary, M. (2013). Randomized controlled trial of cognitive behaviour therapy for comorbid post-traumatic stress disorder and alcohol use disorders. *Addiction*, *108*(8), 1397-1410. https://doi.org/10.1111/add.12167	No full PTSD-diagnosis required for inclusion
Santarnecchi, E., Bossini, L., Vatti, G., Fagiolini, A., La Porta, P., Di Lorenzo, G., Siracusano, A., Rossi, S., & Rossi, A. (2019). Psychological and Brain Connectivity Changes Following Trauma-Focused CBT and EMDR Treatment in Single-Episode PTSD Patients. *Front Psychol*, *10*, 129. https://doi.org/10.3389/fpsyg.2019.00129	Not a randomized controlled trial
Saraiya, T. C., Badour, C. L., Jones, A. C., Jarnecke, A. M., Brown, D. G., Flanagan, J. C., Killeen, T. K., & Back, S. E. (2022). The role of posttraumatic guilt and anger in integrated treatment for PTSD and co-occurring substance use disorders among primarily male veterans. *Psychological Trauma: Theory, Research, Practice, and Policy*. https://doi.org/10.1037/tra0001204	No dichotomous clinician-rated or self-reported response outcome related to change in PTSD symptoms
Saul, H., Cassidy, S., Deeney, B., Kwint, J., & Bisson, J. (2023). Online cognitive behavioural therapy for post-traumatic stress disorder is as effective as face-to-face therapy. *BMJ (Clinical research ed.)*, *380*, 266-266. https://doi.org/https://dx.doi.org/10.1136/bmj.p266	Not a randomized controlled trial
Schulz-Heik, R. J., Avery, T. J., Jo, B., Mahoney, L., & Bayley, P. J. (2022). Posttraumatic Stress Disorder Does Not Compromise Behavioral Pain Treatment: Secondary Analysis of a Randomized Clinical Trial Among Veterans. *Global Advances in Health and Medicine*, *11*. https://doi.org/https://doi.org/10.1177/21649561221075578	Secondary analyses
Schulz-Heik, R. J., Lazzeroni, L. C., Hernandez, B., Avery, T. J., Mathersul, D. C., Tang, J. S., Hugo, E., & Bayley, P. J. (2022). Valued living among veterans in breath-based meditation treatment or cognitive processing therapy for posttraumatic stress disorder: exploratory outcome of a randomized controlled trial. *Global Advances in Health and Medicine*, *11*. https://doi.org/https://dx.doi.org/10.1177/2164957X221108376	No clinician-based PTSD-diagnosis based on a structured interview at inclusion
Shapiro, E., & Laub, B. (2015). Early EMDR intervention following a community critical incident: A randomized clinical trial. *Journal of EMDR Practice and Research*, *9*(1), 17-27. https://doi.org/10.1891/1933-3196.9.1.17	No full PTSD-diagnosis required for inclusion
Shemesh, E., Annunziato, R. A., Weatherley, B. D., Cotter, G., Feaganes, J. R., Santra, M., Yehuda, R., & Rubinstein, D. (2011). A randomized controlled trial of the safety and promise of cognitive-behavioral therapy using imaginal exposure in patients with posttraumatic stress disorder resulting from cardiovascular illness. *J Clin Psychiatry*, *72*(2), 168-174. https://doi.org/10.4088/JCP.09m05116blu	No dichotomous clinician-rated or self-reported response outcome related to change in PTSD symptoms
Simpson, T. L., Kaysen, D. L., Fleming, C. B., Rhew, I. C., Jaffe, A. E., Desai, S., Hien, D. A., Berliner, L., Donovan, D., & Resick, P. A. (2022). Cognitive Processing Therapy or Relapse Prevention for comorbid Posttraumatic Stress Disorder and Alcohol Use Disorder: A randomized clinical trial. *PLoS ONE*, *17*(11), e0276111-e0276111. https://doi.org/https://dx.doi.org/10.1371/journal.pone.0276111	No dichotomous clinician-rated or self-reported response outcome related to change in PTSD symptoms
Sjomark, J., Svanberg, A., Viirman, F., Larsson, M., Poromaa, I., Skalkidou, A., Jonsson, M., & Parling, T. (2022). Antepartum and labour-related single predictors of non-participation, dropout and lost to follow up in a randomised controlled trial comparing internet-based cognitive-behaviour therapy with treatment as usual for women with negative birth experiences and. *BMJ Open*, *12*(11), e063214-e063214. https://doi.org/https://dx.doi.org/10.1136/bmjopen-2022-063214	Secondary analyses

Citation	Reason for exclusion
Sjomark, J., Svanberg, A. S., Larsson, M., Viirman, F., Poromaa, I. S., Skalkidou, A., Jonsson, M., & Parling, T. (2022). Effect of internet-based cognitive behaviour therapy among women with negative birth experiences on mental health and quality of life - a randomized controlled trial. *BMC pregnancy and childbirth*, *22*(1), 835-835. https://doi.org/https://dx.doi.org/10.1186/s12884-022-05168-y	No full PTSD-diagnosis required for inclusion
Sloan, D. M., Marx, B. P., Acierno, R., Messina, M., & Cole, T. A. (2021). Comparing written exposure therapy to Prolonged Exposure for the treatment of PTSD in a veteran sample: A non-inferiority randomized design. *Contemporary Clinical Trials Communications*, *22*, 100764-100764. https://doi.org/https://dx.doi.org/10.1016/j.conctc.2021.100764	Not a randomized controlled trial
Sloan, D. M., Marx, B. P., Resick, P. A., Young-McCaughan, S., Dondanville, K. A., Straud, C. L., Mintz, J., Litz, B. T., & Peterson, A. L. (2022). Effect of Written Exposure Therapy vs Cognitive Processing Therapy on Increasing Treatment Efficiency Among Military Service Members With Posttraumatic Stress Disorder: A Randomized Noninferiority Trial. *JAMA network open*, *5*(1), e2140911-e2140911. https://doi.org/https://dx.doi.org/10.1001/jamanetworkopen.2021.40911	No dichotomous clinician-rated or self-reported response outcome related to change in PTSD symptoms
Sloan, D. M., Thompson-Hollands, J., Hayes, A. M., Lee, D. J., Alpert, E., & Marx, B. P. (2022). Sudden Gains in Two Trauma-Focused Treatments for Posttraumatic Stress Disorder. *Behavior therapy*, *53*(2), 255-266. https://doi.org/https://dx.doi.org/10.1016/j.beth.2021.08.003	No dichotomous clinician-rated or self-reported response outcome related to change in PTSD symptoms
Splaine, C., Smith, D. L., & Held, P. (2023). The role of time since trauma on treatment outcomes of veterans in two intensive posttraumatic stress disorder treatment programs. *Journal of Traumatic Stress*, *36*(1), 83-93. https://doi.org/https://dx.doi.org/10.1002/jts.22881	Not a randomized controlled trial
Stecker, T., McHugo, G., Xie, H., Whyman, K., & Jones, M. (2014). RCT of a brief phone-based CBT intervention to improve PTSD treatment utilization by returning service members. *Psychiatr Serv*, *65*(10), 1232-1237. https://doi.org/10.1176/appi.ps.201300433	No clinician-based PTSD-diagnosis based on a structured interview at inclusion
Steuwe, C., Berg, M., Beblo, T., & Driessen, M. (2021). Narrative Exposure Therapy in Patients With Posttraumatic Stress Disorder and Borderline Personality Disorder in a Naturalistic Residential Setting: A Randomized Controlled Trial. *Frontiers in Psychiatry*, *12*, 765348-765348. https://doi.org/https://doi.org/10.3389/fpsyt.2021.765348	No dichotomous clinician-rated or self-reported response outcome related to change in PTSD symptoms
Stirman, S. W., Cohen, Z. D., Lunney, C. A., DeRubeis, R. J., Wiley, J. F., & Schnurr, P. P. (2021). A personalized index to inform selection of a trauma-focused or non-trauma-focused treatment for PTSD. *Behaviour Research and Therapy*, *142*. https://doi.org/10.1016/j.brat.2021.103872	No dichotomous clinician-rated or self-reported response outcome related to change in PTSD symptoms
Sullan, M. J., Crocker, L. D., Thomas, K. R., Orff, H. J., Davey, D. K., Jurick, S. M., Twamley, E. W., Norman, S. B., Schiehser, D. M., Aupperle, R., & Jak, A. J. (2021). Baseline sleep quality moderates symptom improvement in veterans with comorbid PTSD and TBI receiving trauma-focused treatment. *Behaviour Research and Therapy*, *143*, 103892-103892. https://doi.org/https://dx.doi.org/10.1016/j.brat.2021.103892	Secondary analyses
Surís, A., Link-Malcolm, J., Chard, K., Ahn, C., & North, C. (2013). A randomized clinical trial of cognitive processing therapy for veterans with PTSD related to military sexual trauma. *J Trauma Stress*, *26*(1), 28-37. https://doi.org/10.1002/jts.21765	No dichotomous clinician-rated or self-reported response outcome related to change in PTSD symptoms
Susanty, E., Sijbrandij, M., Srisayekti, W., Suparman, Y., & Huizink, A. C. (2022). The Effectiveness of Eye Movement Desensitization for Post-traumatic Stress Disorder in Indonesia: A Randomized Controlled Trial. *Front Psychol*, *13*, 845520. https://doi.org/10.3389/fpsyg.2022.845520	No dichotomous clinician-rated or self-reported response outcome related to change in PTSD symptoms
Tarrier, N., Pilgrim, H., Sommerfield, C., Faragher, B., Reynolds, M., Graham, E., & Barrowclough, C. (1999). A randomized trial of cognitive therapy and imaginal exposure in the treatment of chronic posttraumatic stress disorder. *J Consult Clin Psychol*, *67*(1), 13-18. https://doi.org/10.1037//0022-006x.67.1.13	Did not apply DSM-IV, DSM-5, or ICD-10 criteria for PTSD
Thorisdottir, A. S., & Asmundson, G. (2022). Internet-delivered cognitive processing therapy for individuals with a history of bullying victimization: a randomized controlled trial. *Cognitive behaviour therapy*, *51*(2), 143-169. https://doi.org/https://dx.doi.org/10.1080/16506073.2021.1938663	No full PTSD-diagnosis required for inclusion

Citation	Reason for exclusion
Thorp, S. R., Glassman, L. H., Wells, S. Y., Walter, K. H., Gebhardt, H., Twamley, E., Golshan, S., Pittman, J., Penski, K., Allard, C., Morland, L. A., & Wetherell, J. (2019). A randomized controlled trial of prolonged exposure therapy versus relaxation training for older veterans with military-related PTSD. *J Anxiety Disord*, *64*, 45-54. https://doi.org/10.1016/j.janxdis.2019.02.003	No dichotomous clinician-rated or self-reported response outcome related to change in PTSD symptoms
van Denderen, M., de Keijser, J., Stewart, R., & Boelen, P. A. (2018). Treating complicated grief and posttraumatic stress in homicidally bereaved individuals: A randomized controlled trial. *Clin Psychol Psychother*, *26*(25), 497-508. https://doi.org/10.1002/cpp.2183	No clinician-based PTSD-diagnosis based on a structured interview at inclusion
Vera, M., Oben, A., Juarbe, D., Hernandez, N., Kichic, R., & Hembree, E. A. (2022). A randomized clinical trial of prolonged exposure and applied relaxation for the treatment of Latinos with posttraumatic stress disorder. *Journal of Traumatic Stress, 35*(2), 593-604. https://doi.org/https://dx.doi.org/10.1002/jts.22773	No dichotomous clinician-rated or self-reported response outcome related to change in PTSD symptoms
Walters, E. M., Jenkins, M. M., Nappi, C. M., Clark, J., Lies, J., Norman, S. B., & Drummond, S. P. A. (2020). The impact of prolonged exposure on sleep and enhancing treatment outcomes with evidence-based sleep interventions: A pilot study. *Psychol Trauma*, *12*(2), 175-185. https://doi.org/10.1037/tra0000478	No dichotomous clinician-rated or self-reported response outcome related to change in PTSD symptoms
Watkins, L. L., LoSavio, S. T., Calhoun, P., Resick, P. A., Sherwood, A., Coffman, C. J., Kirby, A. C., Beaver, T. A., Dennis, M. F., & Beckham, J. C. (2023). Effect of cognitive processing therapy on markers of cardiovascular risk in posttraumatic stress disorder patients: A randomized clinical trial. *Journal of Psychosomatic Research*, *170*, 111351. https://doi.org/https://dx.doi.org/10.1016/j.jpsychores.2023.111351	No dichotomous clinician-rated or self-reported response outcome related to change in PTSD symptoms
Wells, S. Y., Walter, K. H., Dedert, E. A., Strasshofer, D. R., Schnitzer, J. S., Thorp, S. R., Morland, L. A., & Glassman, L. H. (2022). Do older veterans experience change in posttraumatic cognitions following treatment for posttraumatic stress disorder? *Psychological trauma : theory, research, practice and policy*, *14*(4), 605-614. https://doi.org/https://dx.doi.org/10.1037/tra0001119	Secondary analyses
Wheaton, M. G., Choo, T.-H., & Markowitz, J. C. (2023). Changes in avoidance and distress related to trauma reminders in PTSD psychotherapy. *Journal of Behavior Therapy and Experimental Psychiatry*, *78*, 101805-101805. https://doi.org/https://dx.doi.org/10.1016/j.jbtep.2022.101805	Secondary analyses
Yurtsever, A., Konuk, E., Akyüz, T., Zat, Z., Tükel, F., Çetinkaya, M., Savran, C., & Shapiro, E. (2018). An Eye Movement Desensitization and Reprocessing (EMDR) Group Intervention for Syrian Refugees With Post-traumatic Stress Symptoms: Results of a Randomized Controlled Trial. *Front Psychol*, *9*, 493. https://doi.org/10.3389/fpsyg.2018.00493	No clinician-based PTSD-diagnosis based on a structured interview at inclusion
Zaccari, B., Loftis, J., Haywood, T., Hubbard, K., Clark, J., & Kelly, U. (2022). Synchronous telehealth yoga and cognitive processing group therapies for women veterans with posttraumatic stress disorder: a multisite randomized controlled trial adapted for COVID-19. *Telemedicine Journal and e-Health*. https://doi.org/https://doi.org/10.1089/tmj.2021.0612	Not a randomized controlled trial
Zang, Y., Hunt, N., & Cox, T. (2013). A randomised controlled pilot study: the effectiveness of narrative exposure therapy with adult survivors of the Sichuan earthquake. *BMC Psychiatry*, *13*, 41. https://doi.org/10.1186/1471-244x-13-41	No clinician-based PTSD-diagnosis based on a structured interview at inclusion
Zang, Y., Hunt, N., & Cox, T. (2014). Adapting narrative exposure therapy for Chinese earthquake survivors: a pilot randomised controlled feasibility study. *BMC Psychiatry*, *14*, 262. https://doi.org/10.1186/s12888-014-0262-3	No clinician-based PTSD-diagnosis based on a structured interview at inclusion
Zemestani, M., Mohammed, A. F., Ismail, A. A., & Vujanovic, A. A. (2022). A Pilot Randomized Clinical Trial of a Novel, Culturally Adapted, Trauma-Focused Cognitive-Behavioral Intervention for War-Related PTSD in Iraqi Women. *Behavior therapy*, *53*(4), 656-672. https://doi.org/https://dx.doi.org/10.1016/j.beth.2022.01.009	No dichotomous clinician-rated or self-reported response outcome related to change in PTSD symptoms
Zhao, J., Chen, D.-Y., Li, X.-B., Xi, Y.-J., Verma, S., Zhou, F.-C., & Wang, C.-Y. (2023). EMDR versus waiting list in individuals at clinical high risk for psychosis with post-traumatic stress symptoms: A randomized controlled trial. *Schizophrenia research*, *256*, 1-7. https://doi.org/https://dx.doi.org/10.1016/j.schres.2023.04.003	No full PTSD-diagnosis required for inclusion
Ziemba, S. J., Bradley, N. S., Landry, L. A., Roth, C. H., Porter, L. S., & Cuyler, R. N. (2014). Posttraumatic stress disorder treatment for Operation Enduring Freedom/Operation Iraqi Freedom combat veterans through a civilian community-based telemedicine network. *Telemed J E Health*, *20*(5), 446-450. https://doi.org/10.1089/tmj.2013.0312	Data do not allow conclusions on the number of non-responders per experimental and control group

PTSD = posttraumatic stress disorder; DSM-IV = Diagnostic and Statistical Manual of Mental Disorders, fourth edition; DSM-5 = Diagnostic and Statistical Manual of Mental Disorders, fifth edition; ICD-10 = International Classification of Diseases, 10th revision.

G. Full Table of included studies and variables

Table G1 Overview of Included Studies

Study<br>Treatment arm(s)	N total	Non-response Int<br>n / N (%)	Non-response CG<br>n / N (%)	Operationalization of non-response	Non-response assessment tool	Control group	Country of implementation
Acarturk et al. (2016)	98		46/49 (93.88)			Waiting list	Turkey
EMDR		19/49 (38.78)		Retaining diagnosis	M.I.N.I. PLUS		
Adenauer et al. (2011)	19		8/8 (100.00)			Waiting list	Germany
NET		6/11 (54.55)		Retaining diagnosis	CAPS		
Allen et al. (2022)	30		14/17 (82.35)			Waiting list	Australia
CBT		5/13 (38.46)		Retaining diagnosis	PCL-C		
Back et al. (2019)	42		8/13 (61.54)			Active control	USA
PE		5/29 (17.24)		Retaining diagnosis	CAPS		
Beck et al. (2009)	33		11/16 (68.75)			Waiting list	USA
CBT		2/17 (11.76)		Retaining diagnosis	CAPS		
Belleville et al. (2018)	31		4/15 (26.67)			Active control	Canada
CBT		2/16 (12.50)		Retaining diagnosis	CAPS		
Bisson et al. (2022)	160		14/77 (18.18)			Active control	England
CBT		12/83 (14.46)		Retaining diagnosis	CAPS-5		
Blanchard et al. (2003)	42		16/21 (76.19)			Waiting list	USA
CBT		5/21 (23.81)		Retaining diagnosis	CAPS		
Bohus et al. (2013)	74		37/38 (97.37)			TAU	Germany
CBT		22/36 (61.11)		Symptom reduction not achieved	CAPS		
Brady et al. (2021)	19		-/7 (-)		CAPS-5	Waiting list	England
NET		6/12 (50.00)		Symptom reduction not achieved			
Bryant et al. (2003)	45		9/15 (60.00)			Active control	Australia
PE		5/15 (33.33)		Retaining diagnosis	CAPS		
PE + CT		2/15 (13.33)		Retaining diagnosis	CAPS		
Bryant et al. (2008)	90						Australia
CBT		5/24 (20.83)		Retaining diagnosis	CAPS		
CBT		10/21 (47.62)		Retaining diagnosis	CAPS		
CBT		12/22 (54.55)		Retaining diagnosis	CAPS		
CBT		12/23 (52.17)		Retaining diagnosis	CAPS		
Bryant et al. (2011)	28		8/12 (66.67)			TAU	Thailand
CBT		4/16 (25.00)		Cut-off score not achieved	CAPS		
Bryant et al. (2013)	70						Australia
CBT		9/34 (26.47)		Retaining diagnosis	CAPS		
CBT		7/36 (19.44)		Retaining diagnosis	CAPS		
Bryant et al. (2019)	84		25/28 (89.29)			Waiting list	Australia
CBT		11/27 (40.74)		Retaining diagnosis	CAPS		
CBT		7/29 (24.14)		Retaining diagnosis	CAPS		
Butollo et al. (2016)	141		35/74 (47.30)			Active control	Germany
CPT		26/67 (38.81)		Retaining diagnosis	PDS		
Castillo et al. (2016)	28		-/14 (-)			Waiting list	USA
CBT		7/14 (50.00)		Retaining diagnosis	CAPS		
Chard (2005)	55		20/27 (74.07)			Waiting list	USA
CPT		2/28 (7.14)		Retaining diagnosis	CAPS		
Cloitre et al. (2002)	46		18/24 (75.00)			Waiting list	USA
PE		5/22 (22.73)		Retaining diagnosis	CAPS		

Study<br>Treatment arm(s)	N total	Non-response Int<br>n / N (%)	Non-response CG<br>n / N (%)	Operationalization of non-response	Non-response assessment tool	Control group	Country of implementation
Cloitre et al. (2010)	104		20/38 (52.63)			Active control	USA
PE		22/33 (66.67)		Retaining diagnosis	CAPS		
PE		13/33 (39.39)		Retaining diagnosis	CAPS		
Dell et al. (2022)	105						Australia
PE		23/51 (45.90)		Retaining diagnosis	CAPS-5		
PE		29/54 (53.30)		Retaining diagnosis	CAPS-5		
Dunne et al. (2012)	26		12/13 (92.31)			Waiting list	Australia
CBT		5/13 (38.46)		Retaining diagnosis	SCID		
Ehlers et al. (2003)	55		19/27 (70.37)			Waiting list	England
CT		6/28 (21.43)		Retaining diagnosis	CAPS		
Ehlers et al. (2005)	28		14/14 (100.00)			Waiting list	England
CT		4/14 (28.60)		Retaining diagnosis	CAPS		
Ehlers et al. (2014)	91		28/30 (93.33)			Waiting list	England
CT		7/31 (22.58)		Retaining diagnosis	CAPS		
CT		8/30 (26.67)		Retaining diagnosis	CAPS		
Ehlers et al. (2023)	185		29/93 (31.00)			Active control	England
CT		15/92 (16.00)		Retaining diagnosis	CAPS-5		
Falsetti et al. (2008)	47		17/23 (73.91)			Waiting list	USA
CBT		3/24 (12.50)		Retaining diagnosis	CAPS		
Fecteau & Nicki (1999)	20		10/10 (100.00)			Waiting list	Canada
CBT		5/10 (50.00)		Retaining diagnosis	CAPS		
Feske (2008)	21		9/12 (75.00)			TAU	USA
PE		3/9 (33.33)		Significant change not achieved	PDS		
Foa et al. (2018)	259		32/40 (80.00)			Waiting list	USA
PE		56/109 (51.38)		Retaining diagnosis	PSS-I		
PE		60/110 (54.55)		Retaining diagnosis	PSS-I		
Forbes et al. (2012)	47		20/23 (86.96)			TAU	Australia
CPT		15/24 (62.50)		Retaining diagnosis	CAPS		
Ford et al. (2018)	16		5/11 (45.45)			Active control	USA
PE		2/5 (40.00)		Retaining diagnosis	CAPS		
Franklin et al. (2017)	13		5/7 (71.43)			TAU	USA
PE		0/3 (0.00)		Retaining diagnosis	CAPS		
PE		2/3 (66.67)		Retaining diagnosis	CAPS		
Hensel-Dittmann et al. (2011)	23		10/10 (100.00)			Active control	Germany
NET		11/13 (84.62)		Retaining diagnosis	CAPS		
Hinton et al. (2011)	24		8/12 (66.67)			Active control	USA
CBT		0/12 (0.00)		Symptom reduction not achieved	PCL		
Högberg et al. (2007)	21		8/9 (88.89)			Waiting list	Norway
EMDR		4/12 (33.33)		Retaining diagnosis	SCID		
Hollifield et al. (2007)	42		17/21 (80.95)			Waiting list	USA
CBT		12/21 (57.14)		Cut-off score not achieved	PSS-SR		
Karatzias et al. (2011)	46		14/23 (60.87)			Active control	Scotland
EMDR		13/23 (56.52)		Cut-off score not achieved	CAPS		
Kubany et al. (2004)	86		-/40 (-)			Waiting list	USA
CT		4/46 (8.70)		Retaining diagnosis	CAPS		
Langkaas et al. (2017)	65						Norway
CBT		11/34 (32.35)		Significant change not achieved	PSS-I		
PE		31/10 (32.26)		Significant change not achieved	PSS-I		
Lely et al. (2019)	26		10/12 (83.33)			Active control	Netherlands
NET		12/14 (85.71)		Retaining diagnosis	CAPS		

Study<br>Treatment arm(s)	N total	Non-response Int<br>n / N (%)	Non-response CG<br>n / N (%)	Operationalization of non-response	Non-response assessment tool	Control group	Country of implementation
Lindauer et al. (2005)	24		9/12 (75.00)			Waiting list	Netherlands
BEP		2/12 (16.67)		Retaining diagnosis	SI-PTSD		
Markowitz et al. (2015)	70		20/32 (62.50)			Active control	USA
PE		20/38 (52.63)		Symptom reduction not achieved	CAPS		
Maxwell et al. (2016)	16		1/8 (12.50)			Active control	USA
CPT		1/8 (12.50)		Symptom reduction not achieved	MPSS-SR		
McDonagh et al. (2005)	37		16/20 (80.00)			Waiting list	USA
CBT		9/17 (52.94)		Retaining diagnosis	CAPS		
McGovern et al. (2011)	23		5/10 (50.00)			Active control	USA
CBT		4/13 (30.77)		Retaining diagnosis	CAPS		
McLay et al. (2017)	85		29/42 (69.05)			Active control	USA
PE		27/43 (62.79)		Symptom reduction not achieved	CAPS		
Monson et al. (2006)	63		32/33 (96.97)			Waiting list	USA
CPT		18/30 (60.00)		Retaining diagnosis	CAPS		
Morland et al. (2019)	175						USA
PE		26/58 (44.83)		Retaining diagnosis	CAPS		
PE		26/58 (44.83)		Retaining diagnosis	CAPS		
PE		22/59 (37.29)		Retaining diagnosis	CAPS		
Mueser et al. (2008)	58		21/27 (77.78)			TAU	USA
CBT		21/31 (67.74)		Retaining diagnosis	CAPS		
Mueser et al. (2015)	161		55/75 (73.33)			Active control	USA
CBT		55/86 (63.95)		Retaining diagnosis	CAPS		
Nidich et al. (2018)	134		45/66 (68.18)			Active control	USA
PE		40/68 (58.82)		Symptom reduction not achieved	CAPS		
Nijdam et al. (2012)	93						Netherlands
BEP		6/42 (14.29)		Retaining diagnosis	SI-PTSD		
EMDR		4/51 (7.84)		Retaining diagnosis	SI-PTSD		
Peck et al. (2023)	30		4/10 (40.00)			TAU	USA
PE		6/10 (60.00)		Retaining diagnosis	CAPS-5		
PE		6/10 (60.00)		Retaining diagnosis	CAPS-5		
Peterson et al. (2022)	120						USA
CPT		18/44 (42.00)		Retaining diagnosis	CAPS-5		
CPT		12/32 (38.00)		Retaining diagnosis	CAPS-5		
CPT		23/44 (52.00)		Retaining diagnosis	CAPS-5		
Peterson et al. (2023)	234						USA
PE		44/117 (38.00)		Retaining diagnosis	CAPS-5		
PE		61/117 (52.00)		Retaining diagnosis	CAPS-5		
Popiel et al. (2015)	140		17/30 (56.67)			Active control	Poland
PE		38/110 (34.55)		Retaining diagnosis	SCID		
Rauch et al. (2015)	26		6/15 (40.00)			Active control	USA
PE		1/11 (9.09)		Symptom reduction not achieved	CAPS		
Ready et al. (2018)	81		23/40 (57.50)			Active control	USA
CBT		23/41 (56.10)		Significant change not achieved	CAPS		
Reger et al. (2016)	79		37/47 (78.72)			Waiting list	USA
PE		11/32 (34.38)		Significant change not achieved	CAPS		
Resick et al. (2002)	121		-/40 (-)			Waiting list	USA
CPT		8/41 (19.51)		Retaining diagnosis	CAPS		
PE		7/40 (17.50)		Retaining diagnosis	CAPS		

Study<br>Treatment arm(s)	N total	Non-response Int<br>n / N (%)	Non-response CG<br>n / N (%)	Operationalization of non-response	Non-response assessment tool	Control group	Country of implementation
Resick et al. (2008)	86		11/30 (36.67)			Active control	USA
CPT		8/27 (29.63)		Retaining diagnosis	CAPS		
CT		6/29 (20.69)		Retaining diagnosis	CAPS	CT	
Resick et al. (2015)	108		34/52 (65.38)			Active control	USA
CPT		29/56 (51.79)		Symptom reduction not achieved	PCL		
Resick et al. (2017)	165						USA
CPT		43/83 (51.81)		Retaining diagnosis	PSS-I		
CPT		50/82 (60.98)		Retaining diagnosis	PSS-I		
Rothbaum et al. (2005)	60		18/20 (90.00)			Waiting list	USA
EMDR		5/20 (25.00)		Retaining diagnosis	CAPS		
PE		1/20 (5.00)		Retaining diagnosis	CAPS		
Sack et al. (2016)	92		9/45 (20.00)			Active control	Germany
EMDR		9/47 (19.15)		Retaining diagnosis	SCID		
Schacht et al. (2017)	50						USA
PE		14/27 (51.85)		Symptom reduction not achieved	CAPS		
PE		5/23 (21.74)		Symptom reduction not achieved	CAPS		
Schnurr et al. (2003)	325		102/163 (62.58)			Active control	USA
CBT		99/162 (61.11)		Symptom reduction not achieved	CAPS		
Schnurr et al. (2007)	194		89/111 (80.18)			Active control	USA
PE		44/83 (53.01)		Retaining diagnosis	CAPS		
Schnurr et al. (2022)	916						USA
PE		271/455 (59.60)		Retaining diagnosis	CAPS-5		
CPT		331/461 (71.80)		Retaining diagnosis	CAPS-5		
Sloan, Marx, et al. (2018)	114		44/62 (70.97)			Active control	USA
CPT		26/52(50.00)		Retaining diagnosis	CAPS		
Sloan, Unger, et al. (2018)	198		77/100 (77.00)			Active control	USA
CBT		69/98 (70.41)		Retaining diagnosis	CAPS		
Stenmark et al. (2013)	54		14/21 (66.67)			TAU	Norway
NET		19/33 (57.58)		Retaining diagnosis	CAPS		
Taylor et al. (2003)	45		9/15 (60.00)			Active control	Canada
EMDR		6/15 (40.00)		Retaining diagnosis	CAPS		
PE		2/15 (13.33)		Retaining diagnosis	CAPS		
Taylor et al. (2023)	93						USA
CPT		15/31 (48.40)		Significant change not achieved	PCL		
CPT		15/31 (48.40)		Significant change not achieved	PCL		
CPT		18/31 (58.10)		Significant change not achieved	PCL		
Ter Heide et al. (2016)	62		20/29 (68.97)			Active control	Netherlands
EMDR		21/33 (63.64)		Retaining diagnosis	CAPS		
Thompson-Hollands et al. (2023)	257		69/129 (53.50)			Active control	USA
PE		50/128 (39.10)		Significant change not achieved	PCL		
Trottier et al. (2022)			17/18 (94.40)			Active control	Canada
CBT		7/16 (43.70)		Retaining diagnosis	CAPS-5		
Van den Berg et al. (2015)	155		26/47 (55.32)			Waiting list	Netherlands
EMDR		11/55 (20.00)		Retaining diagnosis	CAPS		
PE		17/53 (32.08)		Retaining diagnosis	CAPS		
Van der Kolk et al. (2007)	50		9/26 (34.62)			Waiting list	USA
EMDR		3/24 (12.50)		Retaining diagnosis	CAPS		
Van Vliet et al. (2021)	121						Netherlands
EMDR		21/64 (33.30)		Retaining diagnosis	CAPS-5		
EMDR		17/57 (31.10)		Retaining diagnosis	CAPS-5		

**Study**<br>**Treatment arm(s)**	**N total**	**Non-response Int**<br>**n / N (%)**	**Non-response CG**<br>**n / N (%)**	**Operationalization of non-response**	**Non-response assessment tool**	**Control group**	**Country of implementation**
Vera et al. (2011)	12		7/7 (100.00)			TAU	Puerto Rico
PE		3/5 (60.00)		Retaining diagnosis	CAPS		
Wells et al. (2015)	20		-/10 (-)			Waiting list	England
PE		3/10 (30.00)		Retaining diagnosis	SCID		
Yehuda et al. (2014)	37		10/12 (83.33)			Waiting list	USA
PE		14/25 (56.00)		Retaining diagnosis	CAPS		
Yuen et al. (2015)	52						USA
PE		9/29 (31.03)		Retaining diagnosis	CAPS		
PE		6/23 (26.09)		Retaining diagnosis	CAPS		
Zaccari et al. (2022)	19		5/9 (55.60)			Active Control	USA
CPT		8/10 (80.0)		Retaining diagnosis	CAPS		

(table continues with further variables)

(table continued with further variables

Study<br>Treatment arm(s)	Overall bias	Type of Analysis	Population	M age	% female	% in committed relationship	% employed	% with college level education	M (z)<br>PTSD severity
Acarturk et al. (2016)	Some concerns	ITT							
EMDR			Refugees	33.3	79.2	71.1	-	4.3	-
Adenauer et al. (2011)	High	PP							
NET			Refugees	30.3	43.8	-	-	-	88.00 (1.75) [1]
Allen et al. (2022)	High	ITT							
CBT			Civil	-	-	-	-	-	59.29 (0.03) [3]
Back et al. (2019)	High	PP							
PE			Veterans & Military	39.7	7.4	25.9	41.5	-	77.40 (0.43) [1]
Beck et al. (2009)	High	PP							
CBT			Civil	-	-	-	-	-	57.30 (-2.07) [1]
Belleville et al. (2018)	Some concerns	PP							
CBT			Civil	31.5	90.0	20.0	85.0	75.0	-
Bisson et al. (2022)	Low	ITT							
CBT			Civil	37.6	63.6	-	-	37.4	35.60 (-1.52) [4]
Blanchard et al. (2003)	High	PP							
CBT			Civil	-	-	-	-	-	-
Bohus et al. (2013)	Some concerns	ITT							
CBT			Civil	35.1	100.0	-	-	-	87.92 (1.74) [1]
Brady et al. (2021)	High	PP							
NET			Civil	26.73	73.3	-	-	-	42.0 (0.99) [4]
Bryant et al. (2003)	Some concerns	PP							
PE			Civil	37.1	-	-	-	-	67.47 (-0.80) [1]
PE + CT			Civil	32.4	-	-	-	-	68.73 (-0.65) [1]
Bryant et al. (2008)	High	PP							
CBT			Civil	33.7	-	-	89.3	-	71.35 (-0.32) [1]
CBT			Civil	35.9	-	-	83.9	-	76.06 (0.95) [1]
CBT			Civil	40.9	-	-	85.7	-	76.79 (NaN) [1]
CBT			Civil	39.1	-	-	77.4	-	73.29 (NaN) [1]
Bryant et al. (2011)	Some concerns	PP							
CBT			Civil	42.3	100.0	13.00	93.0	-	26.80 (-0.21) [2]
Bryant et al. (2013)	High	ITT							
CBT			Civil	41.2	50.0	-	76.0	-	67.69 (-0.78) [1]
CBT			Civil	37.9	58.0	-	77.0	-	73.75 (0.44) [1]
Bryant et al. (2019)	Low	ITT							
CBT			Civil	44.7	12.1	75.8	-	-	80.40 (0.81) [1]
CBT			Civil	42.8	27.3	66.7	-	-	70.50 (-0.27) [1]
Butollo et al. (2016)	High	ITT							
CPT			Civil	33.7	67.2	-	-	-	-
Castillo et al. (2016)	Some concerns	ITT							
CBT			Veterans	36.7	100.0	-	-	-	70.60 (-0.41) [1]
Chard (2005)	Some concerns	PP							
CPT			Civil	-	100.0	-	-	-	65.46 (-1.05) [1]
Cloitre et al. (2002)	Some concerns	PP							
PE			Civil	-	100.0	-	-	-	69.00 (-0.61) [1]
Cloitre et al. (2010)	High	ITT							
PE			Civil	-	100.0	30.0	-	-	64.50 (-1.74) [1]
PE			Civil	-	100.0	39.0	-	-	63.08 (-1.88) [1]

Study<br>Treatment arm(s)	Overall bias	Type of Analysis	Population	M age	% female	% in committed relationship	% employed	% with college level education	M (z)<br>PTSD severity
Dell et al. (2022)	High	ITT							
PE			Veterans & Military	46.7	12.7	-	33.8	27.1	-
PE			Veterans & Military	44.3	11.1	-	33.3	21.0	-
Dunne et al. (2012)	High	ITT							
CBT			Civil	-	-	-	-	-	-
Ehlers et al. (2003)	Some concerns	PP							
CT			Civil	-	-	-	-	-	-
Ehlers et al. (2005)	High	ITT							
CT			Civil	35.4	57.0	64.0	64.0	21.0	-
Ehlers et al. (2014)	High	ITT							
CT			Civil	41.5	58.1	54.8	58.1	25.8	70.60 (-0.41) [1]
CT			Civil	39.7	60.0	60.0	46.7	20.0	78.72 (1.52) [1]
Ehlers et al. (2023)	Low	ITT							
CT			Civil	36.3	74.0	52.0	84.0	40.0	40.20 (0.28) [4]
Falsetti et al. (2008)	High	PP							
CBT			Civil	-	-	-	-	-	-
Fecteau & Nicki (1999)	High	ITT							
CBT			Civil	-	-	-	-	-	70.90 (-0.38) [1]
Feske (2008)	High	PP							
PE			Civil	-	100.0	-	-	-	-
Foa et al. (2018)	Some concerns	ITT							
PE			Veterans & Military	32.9	9.2	74.3	-	65.1	25.31 (-0.51) [2]
PE			Veterans & Military	32.7	14.5	69.1	-	76.3	25.20 (-0.49) [2]
Forbes et al. (2012)	High	PP							
CPT			Veterans & Military	53.1	7.0	62.0	38.0	-	75.53 (0.20) [1]
Ford et al. (2018)	High	PP							
PE			Veterans & Military	-	0.0	-	-	-	72.43 (-0.19) [1]
Franklin et al. (2017)	High	PP							
PE			Veterans & Military	-	-	-	-	-	74.30 (0.05) [1]
PE			Veterans & Military	-	-	-	-	-	69.70 (-0.44) [1]
Hensel-Dittmann et al. (2011)	Some concerns	ITT							
NET			Refugees	-	-	-	-	-	96.47 (2.81) [1]
Hinton et al. (2011)	High	PP							
CBT			Civil	47.6	100.0	-	-	-	69.80 (1.20) [3]
Högberg et al. (2007)	High	PP							
EMDR			Civil	43.0	23.1	61.5	-	-	-
Hollifield et al. (2007)	High	PP							
CBT			Civil	40.9	78.6	25.0	-	71.4	-
Karatzias et al. (2011)	Some concerns	ITT							
EMDR			Civil	41.5	60.9	43.5	65.2	45.5	70.70 (-0.40) [1]
Kubany et al. (2004)	High	PP							
CT			Civil	-	100.0	-	-	-	74.40 (0.06) [1]
Langkaas et al. (2017)	Low	ITT							
CBT			Civil	-	-	-	-	-	33.20 (1.15) [2]
PE			Civil	-	-	-	-	-	34.90 (1.46) [2]
Lely et al. (2019)	Some concerns	PP							
NET			Civil	62.7	27.8	61.1	11.8	-	71.25 (-0.33) [1]
Lindauer et al. (2005)	High	ITT							
BEP			Civil	37.6	41.7	58.3	-	-	-

Study<br>Treatment arm(s)	Overall bias	Type of Analysis	Population	M age	% female	% in committed relationship	% employed	% with college level education	M (z)<br>PTSD severity
Markowitz et al. (2015)	Some concerns	ITT							
PE			Civil	41.8	55.0	13.0	61.0	-	72.10 (-0.23) [1]
Maxwell et al. (2016)	Some concerns	PP							
CPT			Civil	-	-	0.0	-	-	-
McDonagh et al. (2005)	High	PP							
CBT			Civil	39.8	100.0	59.0	76.0	-	69.90 (-0.50) [1]
McGovern et al. (2011)	Some concerns	ITT							
CBT			Civil	39.1	50.0	-	-	-	75.75 (0.22) [1]
McLay et al. (2017)	Some concerns	ITT							
PE			Veterans & Military	32.0	0.0	68.4	89.5	68.4	74.50 (0.07) [1]
Monson et al. (2006)	Low	ITT							
CPT			Veterans & Military	54.9	6.7	70.0	-	-	76.73 (0.35) [1]
Morland et al. (2019)	Some concerns	ITT							
PE			Veterans & Military	46.5	28.1	73.6	-	-	41.80 (0.91) [4]
PE			Veterans & Military	47.3	24.1	67.3	-	-	41.50 (0.64) [4]
PE			Veterans & Military	46.5	22.0	70.2	-	-	40.60 (0.71) [4]
Mueser et al. (2008)	Some concerns	ITT							
CBT			Civil	45.1	75.9	-	5.6	-	74.46 (0.07) [1]
Mueser et al. (2015)	Low	ITT							
CBT			Civil	43.0	70.2	-	-	-	86.06 (1.51) [1]
Nidich et al. (2018)	Low	ITT							
PE			Veterans & Military	48.5	18.0	52.0	-	-	80.60 (0.83) [1]
Nijdam et al. (2012)	High	ITT							
BEP			Civil	37.3	61.4	-	-	25.7	-
EMDR			Civil	38.3	51.4	-	-	34.0	-
Peck et al. (2023)	Some concerns	ITT							
PE			Civil	33.8	60.0	-	20.0	-	41.40 (0.75) [4]
PE			Civil	35.9	60.0	-	20.0	-	44.10(1.60) [4]
Peterson et al. (2022)	Some concerns	ITT							
CPT			Veterans & Military	38.5	5.0	75.0	-	30.0	35.6 (-1.52) [4]
CPT			Veterans & Military	41.9	12.0	81.0	-	40.0	37.60 (-0.81) [4]
CPT			Veterans & Military	41.4	18.0	77.0	-	47.0	37.30 (-0.71) [4]
Peterson et al. (2023)	High	ITT							
PE			Veterans & Military	39.0	24.0	61.0	-	36.0	37.56 (-0.74) [4]
PE			Veterans & Military	39.4	20.0	68.0	-	60.0	37.56 (-0.82) [4]
Popiel et al. (2015)	High	ITT							
PE			Civil	39.9	-	53.4	-	-	-
Rauch et al. (2015)	High	PP							
PE			Veterans & Military	-	-	-	-	-	79.20 (0.66) [1]
Ready et al. (2018)	Some concerns	ITT							
CBT			Veterans & Military	-	-	-	-	-	82.43 (1.06) [1]
Reger et al. (2016)	High	PP							
PE			Veterans & Military	30.9	5.6	72.2	100.0	70.4	78.28 (0.54) [1]
Resick et al. (2002)	High	PP							
CPT			Civil	-	100.0	-	-	-	74.76 (0.11) [1]
PE			Civil	-	100.0	-	-	-	76.60 (1.06) [1]

Study<br>Treatment arm(s)	Overall bias	Type of Analysis	Population	M age	% female	% in committed relationship	% employed	% with college level education	M (z)<br>PTSD severity
Resick et al. (2008)	Some concerns	PP							
CPT			Civil	-	-	-	-	-	70.19 (-0.47) [1]
CT			Civil	-	-	-	-	-	73.87 (0.47) [1]
Resick et al. (2015)	Some concerns	ITT							
CPT			Veterans & Military	31.8	7.0	82.0	100.0	69.0	59.30 (0.03) [3]
Resick et al. (2017)	Some concerns	ITT							
CPT			Veterans & Military	32.6	-	68.1	100.0	80.7	24.20 (-0.74) [2]
CPT			Veterans & Military	33.8	-	67.7	100.0	67.6	24.40 (-0.66) [2]
Rothbaum et al. (2005)	High	PP							
EMDR			Civil	-	100.0	-	-	-	-
PE			Civil	-	100.0	-	-	-	-
Sack et al. (2016)	Some concerns	PP							
EMDR			Civil	39.3	68.1	31.9	70.2	80.8	58.60 (-1.91) [1]
Schacht et al. (2017)	Some concerns	PP							
PE			Civil	36.0	77.0	-	-	-	72.77 (-0.14) [1]
PE			Civil	39.0	82.0	-	-	-	72.29 (0.12) [1]
Schnurr et al. (2003)	Some concerns	ITT							
CBT			Veterans & Military	50.6	0.0	51.5	46.9	-	80.41 (0.81) [1]
Schnurr et al. (2007)	Low	PP							
PE			Veterans & Military	44.6	100.0	31.9	-	-	77.60 (0.46) [1]
Schnurr et al. (2022)	Some concerns	ITT							
PE			Veterans & Military	45.5	20.7	54.1	40.4	47.5	39.90 (0.19)[4]
CPT			Veterans & Military	44.9	20.0	51.4	42.9	41.7	40.30 (0.16) [4]
Sloan, Marx, et al. (2018)	Some concerns	ITT							
CPT			Mixed	42.8	47.6	-	-	25.4	37.10 (-0.94) [4]
Sloan, Unger, et al. (2018)	Low	ITT							
CBT			Veterans & Military	54.4	0.0	75.1	86.	22.5	39.84 (-0.14) [4]
Stenmark et al. (2013)	Low	PP							
NET			Refugees	34.5	33.0	-	-	18.0	83.70 (1.22) [1]
Taylor et al. (2003)	High	PP							
EMDR			Civil	-	-	-	-	-	-
PE			Civil	-	-	-	-	-	-
Taylor et al. (2023)	Some concerns	ITT							
CPT			Veterans & Military	36.1	35.0	67.7	90.3	32.2	47.80 (-1.25) [3]
CPT			Veterans & Military	36.2	16.0	74.1	69.8	16.1	53.00 (NaN) [3]
CPT			Veterans & Military	36.3	29.0	67.7	93.6	22.6	53.70 (NaN) [3]
Ter Heide et al. (2016)	High	ITT							
EMDR			Refugees	43.1	16.7	-	-	-	74.70 (0.10) [1]
Thompson-Hollands et al. (2023)	Low	PP							
PE			Veterans & Military	-	100.0	-	-	-	-
Trottier et al. (2022)	High	ITT							
CBT			Civil	28.5	94.7	15.8	52.6	42.1	43.47 (1.56) [4]
Van den Berg et al. (2015)	High	ITT							
EMDR			Civil	40.4	54.6	21.8	7.3	7.3	72.10 (0.08) [1]
PE			Civil	42.6	56.6	20.8	15.1	13.2	69.60 (-0.54) [1]
Van der Kolk et al. (2007)	Some concerns	PP							
EMDR			Civil	38.7	75.9	-	-	51.7	69.40 (-0.56) [1]
Van Vliet et al. (2021)	High	ITT							
EMDR			Civil	-	35.5	-	20.2	6.9	39.34 (-0.06) [4]
EMDR			Civil	-	33.1	-	19.3	6.0	37.61 (-0.81) [4]

Study<br>Treatment arm(s)	Overall bias	Type of Analysis	Population	M age	% female	% in committed relationship	% employed	% with college level education	M (z)<br>PTSD severity
Vera et al. (2011)	High	PP							
PE			Civil	-	-	-	-	-	53.20 (-2.58) [1]
Wells et al. (2015)	High	PP							
PE			Civil	40.5	36.4	72.7	54.6	-	-
Yehuda et al. (2014)	High	PP							
PE			Veterans & Military	-	-	-	-	-	-
Yuen et al. (2015)	High	ITT							
PE			Veterans & Military	-	-	-	-	-	68.42 (-0.69) [1]
PE			Veterans & Military	-	-	-	-	-	65.27 (-1.41) [1]
Zaccari et al. (2022)	High	ITT							
CPT			Veterans & Military	44.2	100.0	37.5	-	-	79.4 (0.68) [4]

(table continues with further variables)

(table continued with further variables

Study<br>Treatment arm(s)	% comorbid depression	M (z) depression score	M (z) anxiety score	Modification of treatment	Symptoms targeted	Treatment manualisation	Treatment format
Acarturk et al. (2016)							
EMDR	-	29.85 (0.67) [5]	2.65 (0.71) [10]	Other modification	PTSD	Manualised	Individual
Adenauer et al. (2011)							
NET	68.8	27.30 (0.41) [6]	-	None	PTSD	Manualised	Individual
Allen et al. (2022)							
CBT	-	15.91 (0.06) [7]	11.95 (-0.23) [15]	None	PTSD	Manualised	
Back et al. (2019)							
PE	38.9	29.20 (0.54) [5]	-	Other modification	PTSD + SUD	Manualised	Individual
Beck et al. (2009)							
CBT	-	22.40 (-0.84) [5]	22.20 (-0.61) [12]	None	PTSD	Manualised	Group
Belleville et al. (2018)							
CBT	15.0	-	-	None	PTSD	Manualised	Individual
Bisson et al. (2022)							
CBT	-	15.10 (-0.36) [7]	13.40 (1.23) [15]	None	PTSD	Manualised	Individual
Blanchard et al. (2003)							
CBT	-	-	-	None	PTSD	Manualised	Individual
Bohus et al. (2013)							
CBT	-	38.00 (2.33) [5]	-	Module therapy	PTSD + BPD	Manualised	Individual
Brady et al. (2021)							
NET	-	18.00(1.14) [7]	11.00 (-1.18) [15]	None	PTSD	Manualised	Individual
Bryant et al. (2003)							
PE	-	19.93 (-1.34) [5]	55.80 (0.66) [11]	None	PTSD	Manualised	Individual
PE + CT	-	19.33 (-1.84) [5]	53.47 (0.12) [11]	None	PTSD	Manualised	Individual
Bryant et al. (2008)							
CBT	-	21.79 (-0.96) [5]	56.93 (0.95) [11]	Other modification	PTSD	Manualised	Individual
CBT	-	24.23 (-0.68) [5]	59.32 (1.69) [11]	Other modification	PTSD	Manualised	Individual
CBT	-	25.38 (-1.02) [5]	58.25 (NaN) [11]	Other modification	PTSD	Manualised	Individual
CBT	-	24.03 (NaN) [5]	59.10 (NaN) [11]	Other modification	PTSD	Manualised	Individual
Bryant et al. (2011)							
CBT	-	22.30 (-0.86) [5]	-	Other modification	PTSD	Manualised	Individual
Bryant et al. (2013)							
CBT	60.0	28.10 (0.31) [5]	27.67 (0.14) [12]	Module therapy	PTSD	Manualised	Individual
CBT	67.0	26.06 (-0.25) [5]	27.94 (0.31) [12]	Module therapy	PTSD	Manualised	Individual
Bryant et al. (2019)							
CBT	66.7	33.00 (1.31) [5]	-	None	PTSD	Manualised	Individual
CBT	45.5	28.80 (0.41) [5]	-	Dose modification	PTSD	Manualised	Individual
Butollo et al. (2016)							
CPT	52.2	-	-	None	PTSD	Manualised	Individual
Castillo et al. (2016)							
CBT	68.2	-	-	Other modification	PTSD	Manualised	Group
Chard (2005)							
CPT	-	24.43 (-0.43) [5]	-	Other modification	PTSD	Manualised	Combined
Cloitre et al. (2002)							
PE	-	25.00 (-0.31) [5]	57.00 (0.96) [11]	Module therapy	PTSD	Manualised	Individual
Cloitre et al. (2010)							
PE	-	22.10 (-0.90) [5]	50.20 (-0.74) [11]	Module therapy	PTSD	Manualised	Individual
PE	-	18.80 (-1.97) [5]	50.40 (-0.71) [11]	Module therapy	PTSD	Manualised	Individual

Study<br>Treatment arm(s)	% comorbid depression	M (z) depression score	M (z) anxiety score	Modification of treatment	Symptoms targeted	Treatment manualisation	Treatment format
Dell et al. (2022)							
PE	80.3	-	-	None	PTSD	Manualised	Individual
PE	82.0	-	-	Dose modification	PTSD	Manualised	Individual
Dunne et al. (2012)							
CBT	61.5	11.39 (-0.71) [9]	7.54 (-0.71) [9]	None	PTSD	Manualised	Individual
Ehlers et al. (2003)							
CT	-	18.80 (-1.57) [5]	21.60 (-0.69) [12]	None	PTSD	Manualised	Individual
Ehlers et al. (2005)							
CT	50.0	23.70 (-0.58) [5]	24.10 (-0.35) [12]	None	PTSD	Manualised	Individual
Ehlers et al. (2014)							
CT	22.6	21.90 (-0.94) [5]	28.42 (0.25) [12]	None	PTSD	Manualised	Individual
CT	40.0	23.93 (-0.75) [5]	26.23 (-0.13) [12]	Dose modification	PTSD	Manualised	Individual
Ehlers et al. (2023)							
CT	62.0	12.93 (-1.48) [7]	12.36 (0.18) [15]	Other modification	PTSD	Manualised	Individual
Falsetti et al. (2008)							
CBT	-	20.40 (-1.25) [4]	-	Other modification	PTSD + panic attacks	Manualised	Group
Fecteau & Nicki (1999)							
CBT	-	26.30 (-0.05) [5]	30.60 (0.55) [12]	None	PTSD	Manualised	Individual
Feske (2008)							
PE	-	27.22 (0.14) [5]	29.22 (0.36) [12]	None	PTSD	Manualised	Individual
Foa et al. (2018)							
PE	-	29.21 (0.54) [5]	-	None	PTSD	Manualised	Individual
PE	-	29.12 (0.48) [5]	-	Dose modification	PTSD	Manualised	Individual
Forbes et al. (2012)							
CPT	-	26.33 (-0.04) [5]	55.97 (0.71) [11]	None	PTSD	Manualised	Individual
Ford et al. (2018)							
PE	50.0	-	20.71 (NaN) [14]	None	PTSD + anger problems	Manualised	Individual
Franklin et al. (2017)							
PE	-	34.00 (1.52) [5]	27.00 (0.05) [12]	None	PTSD	Manualised	Individual
PE	-	35.30 (1.94) [5]	32.40 (1.47) [12]	None	PTSD	Manualised	Individual
Hensel-Dittmann et al. (2011)							
NET	86.7	29.64 (0.88) [6]	-	None	PTSD	Manualised	Individual
Hinton et al. (2011)							
CBT	-	-	2.50 (NaN) [13]	Cultural adaption	PTSD	Manualised	Group
Högberg et al. (2007)							
EMDR	-	29.50 (0.86) [6]	16.70 (-1.36) [12]	None	PTSD	Manualised	Individual
Hollifield et al. (2007)							
CBT	-	2.63 (NaN) [10]	2.40 (-0.71) [10]	None	PTSD	Manualised	Group
Karatzias et al. (2011)							
EMDR	-	11.30 (-1.08) [8]	15.60 (1.15) [8]	None	PTSD	Manualised	Individual
Kubany et al. (2004)							
CT	71.7	26.90 (0.07) [5]	-	Other modification	PTSD	Manualised	Individual
Langkaas et al. (2017)							
CBT	-	23.60 (-0.83) [5]	-	Other modification	PTSD	Manualised	Individual
PE	-	25.60 (-0.19) [5]	-	None	PTSD	Manualised	Individual
Lely et al. (2019)							
NET	61.1	-	-	None	PTSD	Manualised	Individual
Lindauer et al. (2005)							
BEP	25.0	11.80 (0.20) [8]	13.1 (-0.55) [8]	None	PTSD	Manualised	Individual

Study<br>Treatment arm(s)	% comorbid depression	M (z) depression score	M (z) anxiety score	Modification of treatment	Symptoms targeted	Treatment manualisation	Treatment format
Markowitz et al. (2015)							
PE	53.0	20.20 (-1.02) [6]	-	None	PTSD	Manualised	Individual
Maxwell et al. (2016)							
CPT	-	-	-	None	PTSD	Manualised	Individual
McDonagh et al. (2005)							
CBT	-	18.90 (-1.55) [5]	53.50 (0.09) [11]	None	PTSD	Manualised	Individual
McGovern et al. (2011)							
CBT	-	21.10 (-1.04) [5]	-	Other modification	PTSD + SUD	Manualised	Individual
McLay et al. (2017)							
PE	-	-	-	None	PTSD	Manualised	Individual
Monson et al. (2006)							
CPT	53.3	25.39 (-0.23) [5]	54.38 (0.31) [11]	None	PTSD	Manualised	Individual
Morland et al. (2019)							
PE	-	31.70 (1.04) [5]	-	Other modification	PTSD	Manualised	Individual
PE	-	30.70 (0.86) [5]	-	Other modification	PTSD	Manualised	Individual
PE	-	29.70 (0.04) [5]	-	Other modification	PTSD	Manualised	Individual
Mueser et al. (2008)							
CBT	55.6	31.48 (1.01) [5]	48.29 (2.98) [12]	None	PTSD	Manualised	Individual
Mueser et al. (2015)							
CBT	-	30.54 (0.81) [5]	29.20 (0.35) [12]	None	PTSD	Manualised	Individual
Nidich et al. (2018)							
PE	50.0	17.00 (0.63) [7]	-	None	PTSD	Manualised	Individual
Nijdam et al. (2012)							
BEP	67.1	12.07 (0.88) [8]	13.01 (-0.61) [8]	None	PTSD	Manualised	Individual
EMDR	52.9	10.93 (NaN) [8]	12.38 (NaN) [8]	None	PTSD	Manualised	Individual
Peck et al. (2023)							
PE	-	29.70 (0.64) [5]	21.50 (-0.70) [12]	None	PTSD + other disorders	Manualised	Individual
PE	-	32.80 (1.36) [5]	25.30 (-0.37) [12]	Other modification	PTSD + other disorders	Manualised	Individual
Peterson et al. (2022)							
CPT	-	35.20 (1.76) [5]	-	None	PTSD	Manualised	Individual
CPT	-	32.40 (1.26) [5]	-	None	PTSD	Manualised	Individual
CPT	-	33.50 (0.97) [5]	-	None	PTSD	Manualised	Individual
Peterson et al. (2023)							
PE	-	-	-	Dose modification	PTSD	Manualised	Individual
PE	-	-	-	Dose modification	PTSD	Manualised	Individual
Popiel et al. (2015)							
PE	-	26.30 (-0.05) [5]	54.70 (0.39) [11]	None	PTSD	Manualised	Individual
Rauch et al. (2015)							
PE	-	-	-	None	PTSD	Manualised	Individual
Ready et al. (2018)							
CBT	-	29.76 (0.66) [5]	-	None	PTSD	Manualised	Group
Reger et al. (2016)							
PE	-	28.02 (0.30) [5]	22.11 (-0.62) [12]	None	PTSD	Manualised	Individual
Resick et al. (2002)							
CPT	43.5	23.70 (-0.58) [5]	-	None	PTSD	Manualised	Individual
PE	47.5	24.03 (-0.73) [5]	-	None	PTSD	Manualised	Individual
Resick et al. (2008)							
CPT	-	27.51 (0.20) [5]	50.67 (-0.62) [11]	None	PTSD	Manualised	Individual
CT	-	25.72 (-0.33) [5]	50.85 (-0.59) [11]	None	PTSD	Manualised	Individual

Study<br>Treatment arm(s)	% comorbid depression	M (z) depression score	M (z) anxiety score	Modification of treatment	Symptoms targeted	Treatment manualisation	Treatment format
Resick et al. (2015)							
CPT	-	27.90 (0.28) [5]	-	None	PTSD	Manualised	Group
Resick et al. (2017)							
CPT	-	29.20 (0.54) [5]	-	Cultural adaption	PTSD	Manualised	Individual
CPT	-	29.50 (0.57) [5]	-	Cultural adaption	PTSD	Manualised	Group
Rothbaum et al. (2005)							
EMDR	-	16.70 (-0.27) [5]	43.33 (-0.52) [11]	None	PTSD	Manualised	Individual
PE	-	25.95 (-2.00) [5]	51.10 (-2.45) [11]	None	PTSD	Manualised	Individual
Sack et al. (2016)							
EMDR	44.7	24.00 (-0.51) [5]	-	None	PTSD	Manualised	Individual
Schacht et al. (2017)							
PE	47.0	-	-	None	PTSD	Manualised	Individual
PE	50.0	-	-	Cultural adaption	PTSD	Manualised	Individual
Schnurr et al. (2003)							
CBT	58.6	-	-	None	PTSD	Manualised	Group
Schnurr et al. (2007)							
PE	61.7	25.30 (-0.25) [5]	52.10 (-0.26) [11]	None	PTSD	Manualised	Individual
Schnurr et al. (2022)							
PE	67.9	30.30 (0.69) [5]	-	None	PTSD	Manualised	Individual
CPT	72.0	30.0 (0.77) [5]	-	None	PTSD	Manualised	Individual
Sloan, Marx, et al. (2018)							
CPT	-	-	-	None	PTSD	Manualised	Individual
Sloan, Unger, et al. (2018)							
CBT	55.1	23.85 (-0.55) [5]	18.22 (-1.15) [12]	None	PTSD	Manualised	Group
Stenmark et al. (2013)							
NET	41.7	19.70 (-1.13) [6]	-	None	PTSD	Manualised	-
Taylor et al. (2003)							
EMDR	-	23.20 (-0.17) [5]	-	None	PTSD	Manualised	Individual
PE	-	26.40 (-0.68) [5]	-	None	PTSD	Manualised	Individual
Taylor et al. (2023)							
CPT	-	-	-	None	PTSD	Manualised	Individual
CPT	-	-	-	Additional symptoms targeted	PTSD + specific problems	Manualised	Individual
CPT	-	-	-	Additional symptoms targeted	PTSD + specific problems	Manualised	Individual
Ter Heide et al. (2016)							
EMDR	77.8	-	-	None	PTSD	Manualised	Individual
Thompson-Hollands et al. (2023)							
PE	-	-	-	None	PTSD	Manualised	Individual
Trottier et al. (2022)							
CBT	-	27.53 (0.71) [9]	21.26 (0.71) [9]	Additional symptoms targeted	PTSD + other disorders	Manualised	Individual
Van den Berg et al. (2015)							
EMDR	-	28.20 (0.26) [5]	-	None	PTSD	Manualised	Individual
PE	-	30.90 (0.89) [5]	-	None	PTSD	Manualised	Individual
Van der Kolk et al. (2007)							
EMDR	-	16.20 (-2.10) [5]	-	None	PTSD	Manualised	Individual
Van Vliet et al. (2021)							
EMDR	-	-	-	None	PTSD	Manualised	Individual
EMDR	-	-	-	Module Therapy	PTSD	Manualised	Individual
Vera et al. (2011)							
PE	-	-	-	Cultural adaption	PTSD	Manualised	Individual

Study<br>Treatment arm(s)	% comorbid depression	M (z) depression score	M (z) anxiety score	Modification of treatment	Symptoms targeted	Treatment manualisation	Treatment format
Wells et al. (2015)							
PE	36.4	32.50 (1.21) [5]	31.50 (0.67) [12]	None	PTSD	Manualised	Individual
Yehuda et al. (2014)							
PE	-	-	-	None	PTSD	Manualised	Individual
Yuen et al. (2015)							
PE	-	29.56 (0.61) [5]	27.68 (0.15) [12]	None	PTSD	Manualised	Individual
PE	-	26.94 (-0.04) [5]	21.75 (-1.28) [12]	None	PTSD	Manualised	Individual
Zaccari et al. (2022)							
CPT	-	34.90 (1.70) [5]	-	None	PTSD	Manualised	Group

(table continues with further variables)

(table continued with further variables)

**Study** **Treatment arm(s)**	**Time limitation**	**M nr. of Sessions**	**Duration of sessions (min.)**	**Duration of treatment (weeks)**	**Homework**	**Treatment setting**	**Experience level of therapist**
Acarturk et al. (2016)							
EMDR	-	4.2	-	-	No homework	In person	Trainees
Adenauer et al. (2011)							
NET	Low (≤ 12 sessions)	12.0	108.0	-	No homework	In person	Trainees
Allen et al. (2022)							
CBT	Low (≤ 12 sessions)	6.0	-	10.0	Homework	Telehealth	-
Back et al. (2019)							
PE	Low (≤ 12 sessions)	8.8	90.0	-	No homework	In person	Trainees
Beck et al. (2009)							
CBT	High (> 12 sessions)	14.0	120.0	30.0	Homework	In person	Experienced
Belleville et al. (2018)							
CBT	High (> 12 sessions)	15.0	75.0	15.0	No homework	In person	Trainees
Bisson et al. (2022)							
CBT	Low (≤ 12 sessions)	9.0	85.0	12.0	Homework	In person	Experienced
Blanchard et al. (2003)							
CBT	Low (≤ 12 sessions)	10.0	-	10.0	Homework	In person	Trainees
Bohus et al. (2013)							
CBT	High (> 12 sessions)	25.0	45.0	12.5	Homework	In person	Trainees
Brady et al. (2021)							
NET	High (> 12 sessions)	17.0	105.0	32.0	No homework	In person	Mixed
Bryant et al. (2003)							
PE	Low (≤ 12 sessions)	8.0	90.0	8.0	Homework	In person	Trainees
PE + CT	Low (≤ 12 sessions)	8.0	90.0	8.0	Homework	In person	Trainees
Bryant et al. (2008)							
CBT	Low (≤ 12 sessions)	8.0	100.0	8.0	Homework	In person	Trainees
CBT	Low (≤ 12 sessions)	8.0	100.0	8.0	Homework	In person	Trainees
CBT	Low (≤ 12 sessions)	8.0	100.0	8.0	Homework	In person	Trainees
CBT	Low (≤ 12 sessions)	8.0	100.0	8.0	Homework	In person	Trainees
Bryant et al. (2011)							
CBT	Low (≤ 12 sessions)	6.6	60.0	8.0	No homework	In person	Trainees
Bryant et al. (2013)							
CBT	Low (≤ 12 sessions)	7.2	90.0	12.0	Homework	In person	Trainees
CBT	Low (≤ 12 sessions)	9.1	90.0	12.0	Homework	In person	Trainees
Bryant et al. (2019)							
CBT	High (> 12 sessions)	9.4	90.0	12.0	No homework	In person	Trainees
CBT	High (> 12 sessions)	9.6	60.0	12.0	No homework	In person	Trainees
Butollo et al. (2016)							
CPT	High (> 12 sessions)	15.0	-	-	Homework	In person	Mixed
Castillo et al. (2016)							
CBT	High (> 12 sessions)	16.0	90.0	16.0	Homework	In person	Trainees
Chard (2005)							
CPT	High (> 12 sessions)	27.0	78.9	17.0	Homework	In person	Trainees
Cloitre et al. (2002)							
PE	High (> 12 sessions)	16.0	75.0	12.0	Homework	In person	Trainees
Cloitre et al. (2010)							
PE	High (> 12 sessions)	16.0	-	16.0	Homework	In person	Non-professionals
PE	High (> 12 sessions)	16.0	-	16.0	Homework	In person	Non-professionals

Study<br>Treatment arm(s)	Time limitation	M nr. of Sessions	Duration of sessions (min.)	Duration of treatment (weeks)	Homework	Treatment setting	Experience level of therapist
Dell et al. (2022)							
PE	Low (≤ 12 sessions)	10.0	90.0	10.0	Homework	Combination	-
PE	Low (≤ 12 sessions)	10.0	90.0	2.0	Homework	Combination	-
Dunne et al. (2012)							
CBT	Low (≤ 12 sessions)	9.8	60.0	10.0	Homework	In person	Experienced
Ehlers et al. (2003)							
CT	Low (≤ 12 sessions)	9.0	90.0	12.0	No homework	In person	-
Ehlers et al. (2005)							
CT	Low (≤ 12 sessions)	10.0	62.5	13.0	No homework	In person	-
Ehlers et al. (2014)							
CT	Low (≤ 12 sessions)	10.1	-	14.0	Homework	In person	Mixed
CT	High (> 12 sessions)	10.1	105.0	1.0	Homework	In person	Mixed
Ehlers et al. (2023)							
CT	-	-	-	12.0	No homework	Telehealth	Trainees
Falsetti et al. (2008)							
CBT	Low (≤ 12 sessions)	12.0	90.0	12.0	Homework	In person	Mixed
Fecteau & Nicki (1999)							
CBT	Low (≤ 12 sessions)	4.0	120.0	4.0	Homework	In person	Trainees
Feske (2008)							
PE	Low (≤ 12 sessions)	10.0	90.0	10.0	Homework	In person	Non-professionals
Foa et al. (2018)							
PE	Low (≤ 12 sessions)	10.0	90.0	8.0	Homework	In person	Non-professionals
PE	Low (≤ 12 sessions)	10.0	90.0	2.0	Homework	In person	Non-professionals
Forbes et al. (2012)							
CPT	Low (≤ 12 sessions)	12.0	62.5	6.0	Homework	In person	Trainees
Ford et al. (2018)							
PE	Low (≤ 12 sessions)	10.0	82.5	10.0	No homework	In person	Trainees
Franklin et al. (2017)							
PE	Low (≤ 12 sessions)	10.0	-	12.0	Homework	Telehealth	Experienced
PE	Low (≤ 12 sessions)	10.0	-	12.0	Homework	Telehealth	Experienced
Hensel-Dittmann et al. (2011)							
NET	Low (≤ 12 sessions)	10.0	90.0	13.0	No homework	In person	-
Hinton et al. (2011)							
CBT	High (> 12 sessions)	14.0	60.0	14.0	Homework	In person	Non-professionals
Högberg et al. (2007)							
EMDR	Low (≤ 12 sessions)	5.0	90.0	8.0	No homework	In person	Experienced
Hollifield et al. (2007)							
CBT	Low (≤ 12 sessions)	12.0	120.0	12.0	Homework	In person	-
Karatzias et al. (2011)							
EMDR	Low (≤ 12 sessions)	3.7	60.0	-	No homework	In person	Experienced
Kubany et al. (2004)							
CT	High (> 12 sessions)	9.5	90.0	-	Homework	In person	Non-professionals
Langkaas et al. (2017)							
CBT	Low (≤ 12 sessions)	10.0	105.0	10.0	Homework	In person	Trainees
PE	Low (≤ 12 sessions)	10.0	105.0	10.0	Homework	In person	Trainees
Lely et al. (2019)							
NET	High (> 12 sessions)	9.5	90.0	19.0	No homework	In person	Mixed
Lindauer et al. (2005)							
BEP	High (> 12 sessions)	16.0	52.5	16.0	Homework	In person	Experienced

Study<br>Treatment arm(s)	Time limitation	M nr. of Sessions	Duration of sessions (min.)	Duration of treatment (weeks)	Homework	Treatment setting	Experience level of therapist
Markowitz et al. (2015)							
PE	Low (≤ 12 sessions)	8.3	90.0	14.0	Homework	In person	Mixed
Maxwell et al. (2016)							
CPT	Low (≤ 12 sessions)	10.4	90.0	6.0	Homework	In person	-
McDonagh et al. (2005)							
CBT	High (> 12 sessions)	14.0	105.0	17.5	Homework	In person	Trainees
McGovern et al. (2011)							
CBT	High (> 12 sessions)	13.0	47.5	13.0	Homework	In person	Mixed
McLay et al. (2017)							
PE	Low (≤ 12 sessions)	10.0	90.0	9.0	Homework	In person	Non-professionals
Monson et al. (2006)							
CPT	Low (≤ 12 sessions)	12.0	-	6.0	Homework	In person	Trainees
Morland et al. (2019)							
PE	High (> 12 sessions)	9.8	90.0	9.8	Homework		-
PE	High (> 12 sessions)	8.3	90.0	8.3	Homework	Telehealth	-
PE	High (> 12 sessions)	7.0	90.0	7.0	Homework	Telehealth	-
Mueser et al. (2008)							
CBT	High (> 12 sessions)	14.0	-	-	Homework	In person	Trainees
Mueser et al. (2015)							
CBT	High (> 12 sessions)	14.0	-	-	Homework	In person	Trainees
Nidich et al. (2018)							
PE	Low (≤ 12 sessions)	12.0	90.0	12.0	Homework	In person	Trainees
Nijdam et al. (2012)							
BEP	High (> 12 sessions)	14.7	52.5	-	No homework	In person	Mixed
EMDR	Low (≤ 12 sessions)	6.5	90.0	-	No homework	In person	Mixed
Peck et al. (2023)							
PE	Low (≤ 12 sessions)	12.0	60.0	12.0	Homework	Combination	Mixed
PE	Low (≤ 12 sessions)	12.0	60.0	12.0	Homework	Combination	Mixed
Peterson et al. (2022)							
CPT	Low (≤ 12 sessions)	12.0	60.0	6.0	No homework	In person	-
CPT	Low (≤ 12 sessions)	12.0	60.0	6.0	No homework	In person	-
CPT	Low (≤ 12 sessions)	12.0	60.0	6.0	No homework	Telehealth	-
Peterson et al. (2023)							
PE	High (> 12 sessions)	15.0	90.0	3.0	No homework	In person	Mixed
PE	High (> 12 sessions)	15.0	90.0	3.0	No homework	In person	Mixed
Popiel et al. (2015)							
PE	Low (≤ 12 sessions)	8.6	90.0	11.0	Homework	In person	Experienced
Rauch et al. (2015)							
PE	Low (≤ 12 sessions)	11.0	80.0	-	Homework	In person	-
Ready et al. (2018)							
CBT	High (> 12 sessions)	32.0	180.0	16.0	Homework	In person	-
Reger et al. (2016)							
PE	Low (≤ 12 sessions)	7.5	105.0	-	Homework	In person	Trainees
Resick et al. (2002)							
CPT	Low (≤ 12 sessions)	12.0	87.5	6.0	Homework	In person	Trainees
PE	Low (≤ 12 sessions)	12.0	65.0	6.0	Homework	In person	Trainees
Resick et al. (2008)							
CPT	Low (≤ 12 sessions)	12.0	60.0	6.0	Homework	In person	Experienced
CT	Low (≤ 12 sessions)	12.0	60.0	6.0	Homework	In person	Experienced

Study<br>Treatment arm(s)	Time limitation	M nr. of Sessions	Duration of sessions (min.)	Duration of treatment (weeks)	Homework	Treatment setting	Experience level of therapist
Resick et al. (2015)							
CPT	Low (≤ 12 sessions)	12.0	90.0	6.0	No homework	In person	-
Resick et al. (2017)							
CPT	Low (≤ 12 sessions)	12.0	60.0	6.0	No homework	In person	-
CPT	Low (≤ 12 sessions)	12.0	90.0	6.0	No homework	In person	-
Rothbaum et al. (2005)							
EMDR	Low (≤ 12 sessions)	9.0	90.0	4.5	No Homework	In person	Trainees
PE	Low (≤ 12 sessions)	9.0	90.0	4.5	Homework	In person	Trainees
Sack et al. (2016)							
EMDR	Low (≤ 12 sessions)	4.2	-	4.2	No Homework	In person	Experienced
Schacht et al. (2017)							
PE	Low (≤ 12 sessions)	1.8	60.0	12.0	Homework	In person	-
PE	Low (≤ 12 sessions)	7.1	60.0	12.0	Homework	In person	-
Schnurr et al. (2003)							
CBT	High (> 12 sessions)	21.8	92.0	30.0	Homework	In person	Trainees
Schnurr et al. (2007)							
PE	Low (≤ 12 sessions)	8.0	90.0	10.0	Homework	In person	Trainees
Schnurr et al. (2022)							
PE	High (> 12 sessions)	8.2	90.0	8.0	No Homework	In person	Mixed
CPT	High (> 12 sessions)	9.1	60.0	9.0	No Homework	In person	Mixed
Sloan, Marx, et al. (2018)							
CPT	Low (≤ 12 sessions)	12.0	60.0	12.0	Homework	In person	Trainees
Sloan, Unger, et al. (2018)							
CBT	High (> 12 sessions)	14.0	120.0	16.0	Homework	In person	Non-professionals
Stenmark et al. (2013)							
NET	Low (≤ 12 sessions)	10.0	90.0	10.0	Homework	In person	Non-professionals
Taylor et al. (2003)							
EMDR	Low (≤ 12 sessions)	8.0	90.0	-	Homework	In person	Experienced
PE	Low (≤ 12 sessions)	8.0	90.0	-	Homework	In person	Experienced
Taylor et al. (2023)							
CPT	High (> 12 sessions)	18.0	60.0	12.0	No Homework	In person	-
CPT	High (> 12 sessions)	18.0	60.0	12.0	No Homework	In person	-
CPT	High (> 12 sessions)	18.0	60.0	12.0	No Homework	In person	-
Ter Heide et al. (2016)							
EMDR	Low (≤ 12 sessions)	9.0	80.0	-	No Homework	In person	Mixed
Thompson-Hollands et al. (2023)							
PE	Low (≤ 12 sessions)	7.62	90.0	10.0	Homework	In person	Trainees
Trottier et al. (2022)							
CBT	High (> 12 sessions)	16.0	77.5	14.0	No Homework	In person	Trainees
Van den Berg et al. (2015)							
EMDR	Low (≤ 12 sessions)	7.8	90.0	10.0	No Homework	In person	Trainees
PE	Low (≤ 12 sessions)	7.1	90.0	10.0			Trainees
Van der Kolk et al. (2007)							
EMDR	Low (≤ 12 sessions)	8.0	90.0	8.0	No Homework	In person	Experienced
Van Vliet et al. (2021)							
EMDR	High (> 12 sessions)	17.0	90.0	8.0	No Homework	In person	Trainees
EMDR	High (> 12 sessions)	25.0	90.0	12.0	No Homework	In person	Trainees
Vera et al. (2011)							
PE	High (> 12 sessions)	15.0	105.0	15.0	Homework	In person	Experienced

Study<br>Treatment arm(s)	Time limitation	M nr. of Sessions	Duration of sessions (min.)	Duration of treatment (weeks)	Homework	Treatment setting	Experience level of therapist
Wells et al. (2015)							
PE	Low (≤ 12 sessions)	8.0	60.0	8.0	Homework	In person	Trainees
Yehuda et al. (2014)							
PE	Low (≤ 12 sessions)	12.0	90.0	12.0	Homework	In person	Mixed
Yuen et al. (2015)							
PE	Low (≤ 12 sessions)	10.3	90.0	10.0	Homework	In person	Trainees
PE	Low (≤ 12 sessions)	10.3	90.0	10.0	Homework	Telehealth	Trainees
Zaccari et al. (2022)							
CPT	Low (≤ 12 sessions)	12.0	90.0	12.0	No Homework	In person	-

Total N = sum of participants in control and treatment conditions; n = number of non-responders; N = total number of participants in group; Int = intervention group; CG = control group; CBT = cognitive behavioral therapy; nr. = number; min = minutes; CPT = cognitive processing therapy; CT = cognitive therapy; PE = prolonged exposure therapy; BEP = brief eclectic therapy; EMDR = eye movement desensitization and reprocessing; NET = narrative exposure therapy; M.I.N.I. PLUS = Mini-International Neuropsychiatric Interview Plus; CAPS = Clinician-Administered PTSD Scale; Veterans & Military = Veterans & Military Personnel; PDS = Posttraumatic Diagnostic Scale; SCID = Structured Clinical Interview for DSM; PSS-I = PTSD Symptom Scale-Interview; PCL = PTSD Checklist; PSS-SR = Posttraumatic Symptom Scale-Self Report; SI-PTSD = Structured Interview for Posttraumatic Stress Disorder; MPSS-SR = Modified PTSD Symptom Scale Self-Report; TAU = treatment as usual; ITT = intention-to-treat; PP = per-protocol; NaN = not a number; PTSD = posttraumatic stress disorder; SUD = substance use disorder; BPD = borderline personality disorder.

[1] Assessment tool: Clinician-Administered PTSD Scale (CAPS).
[2] Assessment tool: PTSD Symptom Scale - Interview (PSS-I).
[3] Assessment tool: PTSD Checklist (PCL).
[4] Assessment tool: Clinician-Administered PTSD Scale (CAPS-5).
[5] Assessment tool: Beck Depression Inventory (BDI).
[6] Assessment tool: Hamilton Depression Scale (HDRS / HRSD / HAM-D).
[7] Assessment tool: Patient Health Questionnaire-(PHQ)-9.
[8] Assessment tool: Hospital Anxiety and Depression Scale (HADS).
[9] Assessment tool: Depression Anxiety and Stress Scale (DASS).
[10] Assessment tool: Hopkins Symptom Checklist-25 (HSCL-25).
[11] Assessment tool: State-Trait Anxiety Inventory (STAI).
[12] Assessment tool: Beck Anxiety Inventory (BAI).
[13] Assessment tool: Anxiety subscale of the symptom checklist-90-R (SCL).
[14] Assessment tool: State Trait Anger Expression Trait Scale (STAX-trait)
[15] Assessment tool: Generalized Anxiety Disorder Questionnaire (GAD-7)

H. Forest Plot of *OR*

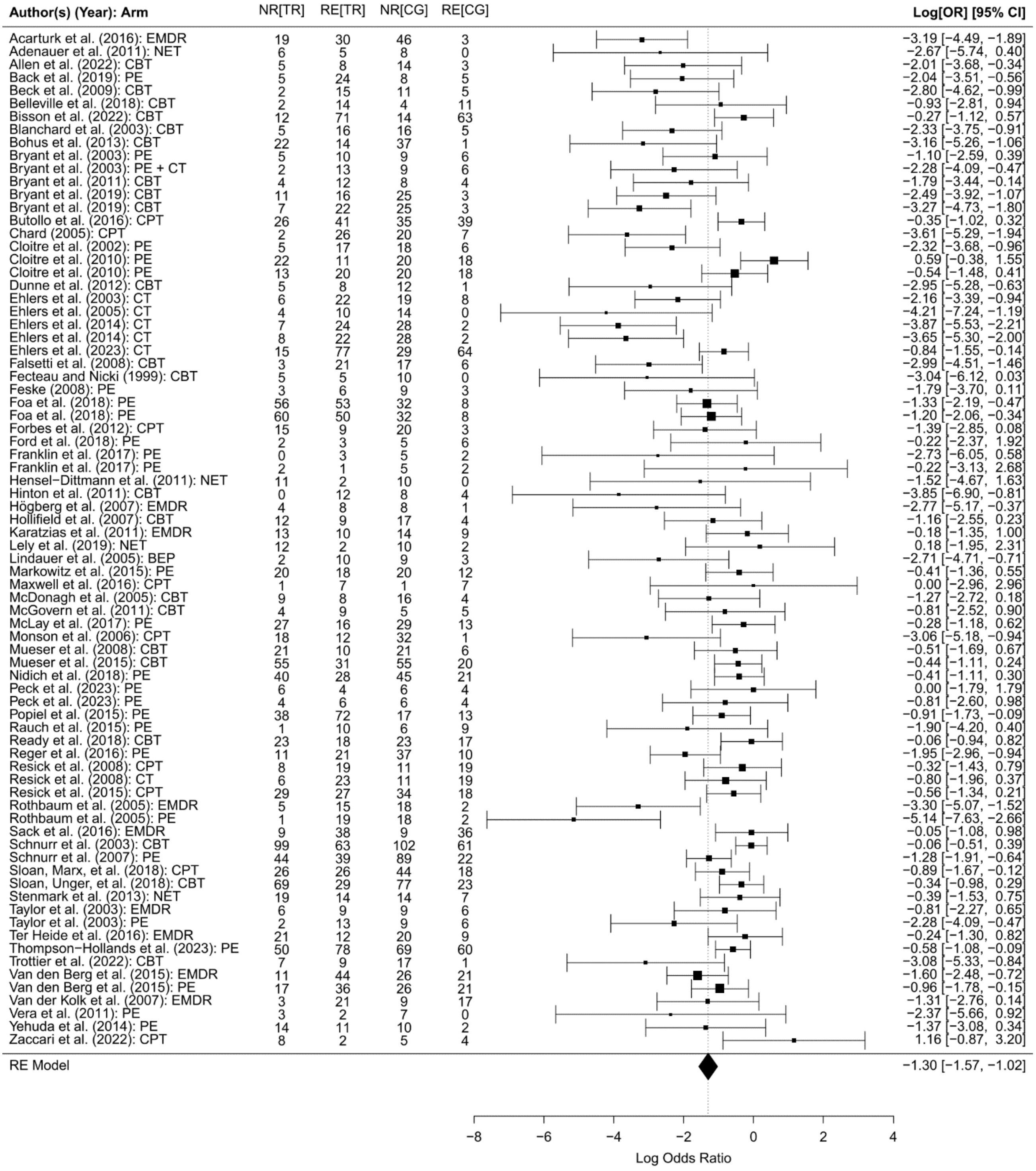

Figure H1 Forest Plot of log OR

NR[TR] = number of non-responders treatment group; RE[TR] = number of responders treatment group; NR[CG] = number of non-responders control group; RE[CG] = number of responders control group; CI = confidence interval; Log *OR* = log transformed Odds Ratio; CBT = cognitive behavioral therapy; CPT = cognitive processing therapy; CT = cognitive therapy; PE = prolonged exposure therapy; BEP = brief eclectic therapy; EMDR = eye movement desensitization and reprocessing; NET = narrative exposure therapy.

I. GRADE: Summary of Findings

Summary of findings 1. Prevalence and predictors of non-response to first-line guideline-recommended psychological treatments for PTSD

Outcomes	№ of participants (studies)	Certainty of the evidence (GRADE)	Results on non-response
**Non-response rate**<br>Follow-up: post-treatment conducted between 0 days and 6 weeks after end of intervention	7894<br>(86 RCTs)	⊕⊕◯◯<br>Low[a,b,c]	• The weighted average non-response rate across all studies in active treatment conditions was 39.23%, 95% CI [35.08%, 43.53%]<br>• Subgroup analyses and meta-regression revealed type of analysis, population, type of intervention, treatment format, year of publication, age, sex, PTSD symptom severity, comorbid depression, and baseline depression score as significant predictors
**Odds ratio**<br>Follow-up: post-treatment conducted between 0 days and 6 weeks after end of intervention	5231<br>(67 RCTs)	⊕⊕◯◯<br>Low[a,c,d]	The pooled *OR* was 0.22, 95% CI [0.17, 0.26], indicating that non-response was less frequent in the treatment condition compared to the control condition.

CI: confidence interval; ***OR*:** odds ratio.

GRADE Working Group grades of evidence
High certainty: we are very confident that the true effect lies close to that of the estimate of the effect.
Moderate certainty: we are moderately confident in the effect estimate: the true effect is likely to be close to the estimate of the effect, but there is a possibility that it is substantially different.
Low certainty: our confidence in the effect estimate is limited: the true effect may be substantially different from the estimate of the effect.
Very low certainty: we have very little confidence in the effect estimate: the true effect is likely to be substantially different from the estimate of effect.

Explanations

a. Downgrade one level for risk of bias because the proportion of information from studies at high risk of bias is sufficient to affect the interpretation of the results.
b. Downgraded one level for inconsistency because of substantial to considerable heterogeneity (I^2 = 83.12%). Not downgraded two levels for inconsistency because extensive predictor analyses were performed to explain heterogeneity.
c. Assessment of publication bias, large effect, plausible confounding criteria not applicable. Explanations: primary outcome is not effect size; primary outcome does not include comparison; analysis of secondary outcome (non-response rate) from included studies, therefore no risk of publication bias.
e. Downgraded one level for inconsistency because of substantial heterogeneity (I^2 = 69.80%).

J. Risk of Bias Assessment

Table J1. Rating of Risk of Bias Domains and Overall Risk of Bias in Percentages

	Low risk	Some concerns	High risk
Number of studies (*n* = 86)			
Bias arising from the randomization process (%)	44.19	45.35	10.47
Bias due to deviations from intended interventions (%)	76.74	3.49	19.77
Bias due to missing outcome data (%)	65.12	11.63	23.26
Bias in measurement of the outcome (%)	59.30	23.26	17.44
Bias in selection of the reported result (%)	86.05	13.95	-
Overall risk of bias (%)	12.79	37.21	50.0

Data from O'Neil ME, Cheney T, Yu Y, et al. *Pharmacologic and Nonpharmacologic Treatments for Posttraumatic Stress Disorder: 2023 Update of the Evidence Base for the PTSD Trials Standardized Data Repository.* Agency for Healthcare Research and Quality (US).; September 2023. https://doi.org/10.23970/AHRQEPCPTSD2023 and from an additional rating for studies not included in the PTSD Repository.

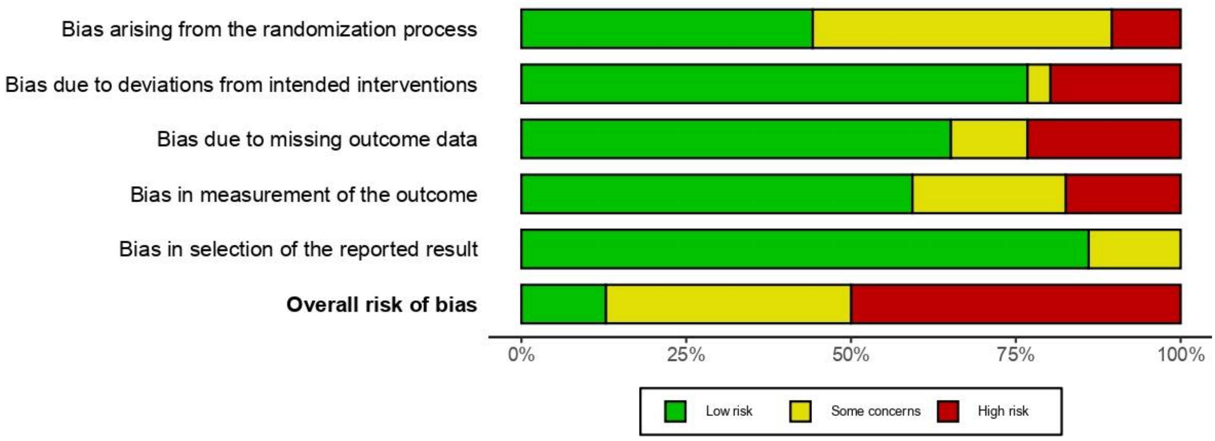

Figure J1 Rating of Risk of Bias Domains and Overall Risk of Bias in Percentages Weighted by Sample Size
Data from O'Neil ME, Cheney T, Yu Y, et al. *Pharmacologic and Nonpharmacologic Treatments for Posttraumatic Stress Disorder: 2023 Update of the Evidence Base for the PTSD Trials Standardized Data Repository.* Agency for Healthcare Research and Quality (US).; September 2023. https://doi.org/10.23970/AHRQEPCPTSD2023 from an additional rating for studies not included in the PTSD Repository.

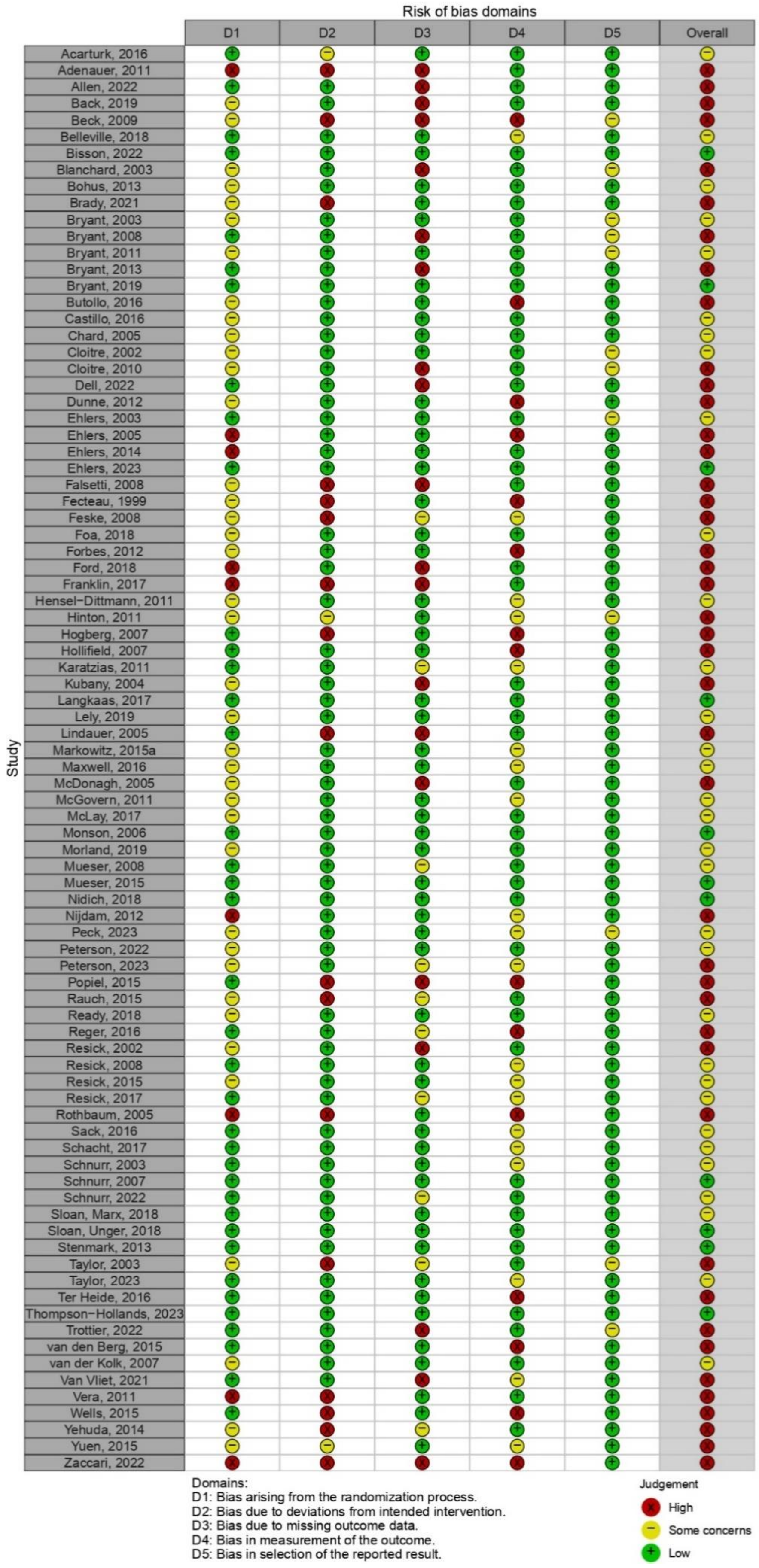

Figure J2 Rating of Risk of Bias Domains and Overall Risk of Bias for Individual Studies

Data from Data from O'Neil ME, Cheney T, Yu Y, et al. *Pharmacologic and Nonpharmacologic Treatments for Posttraumatic Stress Disorder: 2023 Update of the Evidence Base for the PTSD Trials Standardized Data Repository*. Agency for Healthcare Research and Quality (US).; September 2023. https://doi.org/10.23970/AHRQEPCPTSD2023 from an additional rating for studies not included in the PTSD Repository
